# Supplementary material for: Stereoselective Synthesis and Anticancer Activity of 2,6-Disubstituted trans-3-Methylidenetetrahydropyran-4-ones
Source: Materials (Basel). 2022 Apr 21;15(9):3030. doi: 10.3390/ma15093030 (PMC9105583; doi:10.3390/ma15093030)

Stereoselective Synthesis and Anticancer Activity of 2,6-Disubstituted *trans*-3-Methylidenetetrahydropyran-4-ones

Tomasz Bartosik [1,†], Joanna Drogosz-Stachowicz [2,†], Anna Janecka [2], Jacek Kędzia [1], Barbara Pacholczyk-Sienicka [1], Jacek Szymański [3], Katarzyna Gach-Janczak [2] and Tomasz Janecki [1,*]

1 Institute of Organic Chemistry, Lodz University of Technology, Żeromskiego 116, 90-924 Lodz, Poland; tomasz.bartosik@p.lodz.pl (T.B.); jacek.kedzia@p.lodz.pl (J.K.); barbara.pacholczyk@p.lodz.pl (B.P.-S.)
2 Department of Biomolecular Chemistry, Medical University of Łódź, Mazowiecka 6/8, 92-215 Lodz, Poland; joanna.drogosz-stachowicz@stud.umed.lodz.pl (J.D.-S.); anna.janecka@umed.lodz.pl (A.J.); katarzyna.gach-janczak@umed.lodz.pl (K.G.-J.)
3 Central Scientific Laboratory, Medical University of Łódź, Mazowiecka 6/8, 92-215 Lodz, Poland; jacek.szymanski@umed.lodz.pl
* Correspondence: tomasz.janecki@p.lodz.pl; Tel.: +48-426313220
† These authors contributed equally to this work.

Supporting Information

Table of contents:

1.1 Synthesis of diethyl 4-alkyl(aryl)-4-hydroxy-2-oxobutylphosphonates **9a-e** 2

1.2 Synthesis of 6-alkyl(aryl)-3-diethoxyphosphoryldihydropyran-4-ones **11a-e**.................... 3

1.3 Synthesis of 2-alkyl(aryl)-6-alkyl(aryl)-3-diethoxyphosphoryltetrahydropyran-4-ones **12a-j**.................... 3

1.4 Synthesis of 2-isopropyl-6-alkyl(aryl)-3-diethoxyphosphoryltetrahydropyran-4-ones **12k-o**.................... 8

1.5 Synthesis of 2-alkyl(aryl)-5-diethoxyphosphoryltetrahydropyran-4-ones **14a-d**. 12

1.6 NMR Spectra.................... 15

1.1 Synthesis of diethyl 4-alkyl(aryl)-4-hydroxy-2-oxobutylphosphonates 9a-e

In a round-bottomed three – necked flask under argon atmosphere NaH (0.51 g, 17.00 mmol, 80% in mineral oil) was suspended in THF (48 mL). The suspension was stirred and solution of diethyl 2-oxopropylphosphonate **7** (3.00 g, 15,45 mmol) in THF (3 mL) was added dropwise. The reaction mixture was stirred for 30 min, cooled below – 30 ℃ in dry ice – acetone bath and n-butyllithium (6.80 mL of 2.5 M solution in hexane, 17.00 mmol) was added dropwise. Reaction mixture was stirred for 30 minutes at this temperature, cooled to -78 ℃ in dry ice – acetone bath and solution of aldehyde (18.54 mmol) in THF (6 mL) was added dropwise. Reaction mixture was stirred for 1.5 h at this temperature. After this time the reaction was quenched by adding saturated solution of ammonium chloride (100 mL). The water layer was extracted with DCM (3 × 100 mL), the organic layers were combined, washed with brine and dried over $MgSO_4$. The solvents were evaporated under reduced pressure and the resulting crude product was purified by column chromatography (eluent ethyl acetate).

Diethyl (4-hydroxy-2-oxohexyl)phosphonate (**9a**) (2.77 g, 71%) Colorless oil. ^{31}P NMR (101 MHz, Chloroform-*d*) δ 20.03. ^{1}H NMR (700 MHz, Chloroform-*d*) δ 0.88 (td, *J* = 7.4, 1.0 Hz, 3H), 1.27 (t, *J* = 7.1 Hz, 6H), 1.36 – 1.51 (m, 2H), 2.63 (ddd, *J* = 17.0, 9.0, 0.9 Hz, 1H), 2.72 (ddd, *J* = 16.8, 3.0, 1.2 Hz, 1H), 2.99 – 3.14 (m, 2H), 3.25 (s, 1H), 3.86 – 3.96 (m, 1H), 4.02 – 4.13 (m, 4H). ^{13}C NMR (176 MHz, Chloroform-*d*) δ 9.80 , 16.29 (d, *J* = 6.3 Hz, 2×C), 29.56 , 43.10 (d, *J* = 126.8 Hz), 50.65 (d, *J* = 2.9 Hz), 62.66 (d, *J* = 5.4 Hz), 62.71 (d, *J* = 5.6 Hz), 68.96 (d, *J* = 5.1 Hz), 202.86 (d, *J* = 6.0 Hz). ESI-MS $[M+Na]^+$ = 275.1. Anal. Calcd for $C_{10}H_{21}O_5P$: C, 47.62; H, 8.39. Found: C, 47.51; H, 8.37.

Diethyl (4-hydroxy-5-methyl-2-oxohexyl)phosphonate (**9b**) (3.00 g, 73%) Colorless oil. ^{31}P NMR (101 MHz, Chloroform-*d*) δ 19.97. ^{1}H NMR (700 MHz, Chloroform-*d*) δ 0.92 (d, *J* = 6.8 Hz, 3H), 0.94 (d, *J* = 6.7 Hz, 3H), 1.34 (td, *J* = 7.1, 1.2 Hz, 6H), 1.70 (pd, *J* = 6.8, 5.6 Hz, 1H), 2.71 (dd, *J* = 17.0, 9.6 Hz, 1H), 2.78 (dd, *J* = 17.0, 2.4 Hz, 1H), 2.99 (d, *J* = 4.3 Hz, 1H), 3.06 – 3.20 (m, 2H), 3.84 (dtd, *J* = 9.5, 5.9, 4.7, 2.7 Hz, 1H), 4.10 – 4.23 (m, 4H). ^{13}C NMR (176 MHz, Chloroform-*d*) δ 16.34 (d, *J* = 6.2 Hz, 2×C), 17.72, 18.40, 33.33, 43.22 (d, *J* = 126.8 Hz), 48.20, 62.76 (d, *J* = 6.4 Hz, 2×C), 72.33, 203.27 (d, *J* = 6.1 Hz). ESI-MS $[M+Na]^+$ = 289.1. Anal. Calcd for $C_{11}H_{23}O_5P$: C, 49.62; H, 8.71. Found: C, 49.83; H, 8.71.

Diethyl (4-hydroxy-2-oxo-4-phenylbutyl)phosphonate (**9c**) (3.53 g, 76%) Pale yellow oil .^{31}P NMR (101 MHz, Chloroform-*d*) δ 19.79. ^{1}H NMR (700 MHz, Chloroform-*d*) δ 1.28 – 1.33 (m, 3H), 1.28 – 1.33 (m, 3H), 2.94 (dd, *J* = 17.1, 3.2 Hz, 1H), 3.04 (dd, *J* = 17.1, 9.4 Hz, 1H), 3.05 – 3.12 (m, 1H), 3.08 – 3.16 (m, 1H), 3.62 (s, 1H), 4.05 – 4.15 (m, 4H), 5.15 (dd, *J* = 9.4, 3.1 Hz, 1H), 7.23 – 7.26 (m, 1H), 7.29 – 7.36 (m, 4H). ^{13}C NMR (176 MHz, Chloroform-*d*) δ 16.37 (d, *J* = 6.1 Hz, 2×C), 43.22 (d, *J* = 126.8 Hz), 52.95, 62.83 (d, *J* = 6.6 Hz), 62.84 (d, *J* = 6.3 Hz), 69.93, 125.73 (2×C), 127.70, 128.57 (2×C), 142.96, 202.03 (d, *J* = 6.0 Hz). ESI-MS $[M+Na]^+$ = 323.0. Anal. Calcd for $C_{14}H_{21}O_5P$: C, 56.00; H, 7.05. Found: C, 55.93; H, 7.04.

Diethyl (4-hydroxy-4-(4-methoxyphenyl)-2-oxobutyl)phosphonate (**9d**) (4.29 g, 84%) Pale yellow oil. ^{31}P NMR (101 MHz, Chloroform-*d*) δ 19.80. ^{1}H NMR (700 MHz, Chloroform-*d*) δ 1.30 (td, *J* = 7.0, 0.4 Hz, 3H), 1.31 (td, *J* = 7.0, 0.4 Hz, 3H), 2.92 (dd, *J* = 16.9, 3.3 Hz, 1H), 3.03 (dd, *J* = 16.9, 9.3 Hz, 1H), 3.05 – 3.15 (m, 2H), 3.53 (d, *J* = 3.6 Hz, 1H), 3.77 (s, 3H), 4.04 – 4.16 (m, 4H), 5.10 (ddd, *J* = 9.3, 3.4, 3.4 Hz, 1H), 6.85 (d, *J* = 8.7 Hz, 2H), 7.27 (d, *J* = 8.9 Hz, 2H). ^{13}C NMR (176 MHz, Chloroform-*d*) δ 16.35 (d, *J* = 6.2 Hz, 2×C), 43.18 (d, *J* = 126.8

Hz), 52.91, 55.33 (d, J = 2.6 Hz), 62.77 (d, J = 6.4 Hz, 2×C), 69.55 (d, J = 2.3 Hz), 113.92 (2×C), 126.99 (2×C), 135.16, 159.15, 202.04 (d, J = 6.0 Hz). ESI-MS $[M+Na]^+$ = 353.0. Anal. Calcd for $C_{15}H_{23}O_6P$: C, 54.54; H, 7.02. Found: C, 54.70; H, 7.03.

Diethyl (4-hydroxy-2-oxo-4-ferrocenylbutyl)phosphonate (**9e**) (5.05 g, 80%) Red oil. ^{31}P NMR (284 MHz, Chloroform-*d*) δ 19.61. ^{1}H NMR (700 MHz, Chloroform-*d*) δ 1.34 (td, J = 7.1, 1.0 Hz, 6H), 2.84 (d, J = 3.9 Hz, 1H), 3.01 (dd, J = 16.7, 3.9 Hz, 1H), 3.05 (dd, J = 16.7, 8.7 Hz, 1H), 3.10 – 3.19 (m, 2H), 4.06 – 4.26 (m, 13H), 4.83 – 4.93 (m, 1H). ^{13}C NMR (176 MHz, Chloroform-*d*) δ 16.41 (d, J = 6.1 Hz), 16.42 (d, J = 6.2 Hz), 43.28 (d, J = 126.6 Hz), 52.01 , 62.78 (d, J = 6.5 Hz), 62.80 (d, J = 6.4 Hz), 66.02, 66.24, 66.53, 68.08, 68.14, 68.63 (5×C), 91.60, 201.55 (d, J = 6.2 Hz). ESI-MS $[M+Na]^+$ = 431.1. Anal. Calcd for $C_{18}H_{25}FeO_5P$: C, 52.96; H, 6.17. Found: C, 52.99; H, 6.19.

1.2 Synthesis of 6-alkyl(aryl)-3-diethoxyphosphoryldihydropyran-4-ones 11a-e.

The diethyl 4-alkyl(aryl)-4-hydroxy-2-oxobutylphosphonates (**9a-d**) (10.00 mmol) was dissolved in dry dichloromethane (100 mL). Next, dimethyl formamide dimethyl acetal (DMF – DMA) (30.00 mmol, 4.24 mL) was added dropwise and stirred for 2 hours. After this time to reaction mixture boron trifluoride etherate ($BF_3 \cdot Et_2O$) (25.00 mmol, 3.10 mL) was added dropwise. In the case of synthesis of 6-alkyl(aryl)-3-diethoxyphosphoryldihydropyran-4-ones **11e**, the reaction was carried without boron trifluoride etherate and reduced amount of dimethyl formamide dimethyl acetal (DMF – DMA) (25.00 mmol, 3.53 mL). Reaction was controlled by ^{31}P NMR. After completion of the reaction, to the reaction mixture was added saturated solution of sodium hydrocarbonate (100 mL). The water layer was extracted with ethyl acetate (3 × 100 mL), the organic layers were combined, washed with brine and dried over $MgSO_4$. The solvents were evaporated under reduced pressure. Obtained crude product was used on next step without further purification.

1.3 Synthesis of 2-alkyl(aryl)-6-alkyl(aryl)-3-diethoxyphosphoryltetrahydropyran-4-ones 12a-j.

To a suspension of copper iodide (I) (0.80 mmol, 305 mg) in THF (10.70 mL) was added dropwise *n*-BuLi 2,5 M in hexane solution (3,00 mmol, 1.25 mL) at 0°C. The solution was stirred at this temperature for 20 minutes. After this time the mixture was cooled to -78 °C, chlorotrimethylsilane (5.00 mmol, 0.65 mL) was added dropwise and stirred for 10 minutes. Next, to obtained mixture was added a solution of 6-alkyl(aryl)-3-diethoxyphosphoryl-dihydropyran-4-one **11a-e** (1.00 mmol) in dry THF (7,00 mL) and stirred at -78 °C for 4 hours. After this time the reaction was quenched with saturated aqueous NH_4Cl (7.50 mL) and stirred for 20 minutes. Next, the mixture was diluted with saturated aqueous NH_4Cl (25,00 mL) and extracted with ethyl acetate (5 x 30 mL). The combined organic layers was washed with H_2O (50,00 mL) and brine (50,00 mL), then dried over $MgSO_4$ and concentrated under reduced pressure. Obtained crude product was purified by column chromatography using as eluent dichloromethane : acetone (20:1) mixture.

The following abbreviations are used to simplify the analysis of the spectra:
trans = r-2-trans-3-trans-6
enol = trans-enol
cis = r-2-cis-3-trans-6

2-Butyl-3-diethoxyphosphoryl-6-ethyltetrahydropyran-4-one (**12a**) (208 mg, 65%) Colourless oil. ^{31}P NMR (284 MHz, Chloroform-*d*) δ 20.09 (cis), 20.09 (trans), 25.17 (enol). ^{1}H NMR (700 MHz, Chloroform-*d*) δ 0.79 – 1.02 (m, 6H trans + 6H cis + 6H enol), 1.19 – 1.73 (m, 14H trans + 14H cis + 14H enol), 2.11 (dddd, *J* = 17.6, 9.9, 2.2, 1.3 Hz, 1H, enol), 2.15 (ddd, *J* = 17.6, 4.2, 1.2 Hz, 1H, enol), 2.23 (dd, *J* = 14.0, 8.9 Hz, 1H, cis), 2.34 (ddt, *J* = 14.2, 2.8, 1.2 Hz, 1H, trans), 2.53 (dd, *J* = 6.6, 3.6 Hz, 1H, cis), 2.57 (dt, *J* = 14.0, 4.0 Hz, 1H, cis), 2.67 (dd, *J* = 14.2, 11.0 Hz, 1H, trans), 2.82 (dt, *J* = 23.8, 1.6 Hz, 1H, trans), 3.30 (ddd, *J* = 21.3, 5.3, 1.0 Hz, 1H, cis), 3.74 (dddd, *J* = 10.7, 7.7, 4.8, 3.2 Hz, 1H, trans), 3.97 – 4.25 (m, 4H trans + 4H cis + 4H enol), 4.60 (dddd, *J* = 14.7, 10.1, 4.9, 1.7 Hz, 1H, trans), 10.97 (d, *J* = 1.1 Hz, 1H, enol). ^{13}C NMR (176 MHz, Chloroform-*d*) δ 9.79 (trans), 9.86 (cis), 10.12 (enol), 14.08 (trans), 14.11 (cis), 14.14 (enol), 16.28 (d, *J* = 7.2 Hz, enol), 16.33 (d, *J* = 7.1 Hz, enol), 16.41 (d, *J* = 6.0 Hz, trans), 16.53 (d, *J* = 6.3 Hz, trans), 22.26 (cis), 22.31 (trans), 22.46 (enol), 27.46 (cis), 27.54 (trans), 28.16 (enol), 28.91 (enol), 28.95 (cis), 29.44 (trans), 33.06 (enol), 33.14 (d, *J* = 13.9 Hz, trans), 46.31 (trans), 56.01 (d, *J* = 140.2 Hz, cis), 56.42 (d, *J* = 125.9 Hz, trans), 61.83 (d, *J* = 4.9 Hz, enol), 62.18 (d, *J* = 4.8 Hz, enol), 62.77 (d, *J* = 6.5 Hz, trans), 63.12 (d, *J* = 6.4 Hz, trans), 67.73 (enol), 71.48 (trans), 71.85 (d, *J* = 14.1 Hz, enol), 72.18 (cis), 73.80 (d, *J* = 4.5 Hz, trans), 92.73 (d, *J* = 177.0 Hz, enol), 202.76 (d, *J* = 3.8 Hz, trans). EI-MS $[M]^+$ = 343.1. Anal. Calcd for $C_{15}H_{29}O_5P$: C, 56.24; H, 9.12. Found: C, 56.08; H, 9.14.

Due to overlap of signals, the signals of cis and enol forms in the ^{1}H NMR spectra have not been assigned.

3-Diethoxyphosphoryl-6-ethyl-2-phenyltetrahydropyran-4-one (**12b**) (286 mg, 84%) Colourless oil.^{31}P NMR (284 MHz, Chloroform-*d*) δ 18.14 (cis), 20.21 (trans), 23.00 (enol). ^{1}H NMR (700 MHz, Chloroform-*d*) δ 0.69 (t, *J* = 7.4 Hz, 3H, enol), 0.78 – 0.83 (m, 3H trans + 3H enol), 1.24 – 1.31 (m, 6H trans + 3H enol), 1.32 – 1.38 (m, 1H, enol), 1.38 – 1.46 (m, 1H trans + 1H enol), 1.53 – 1.61 (m, 1H, trans), 2.15 (ddt, *J* = 17.9, 10.0, 1.5 Hz, 1H, enol), 2.22 (ddd, *J* = 17.9, 4.4, 1.6 Hz, 1H, enol), 2.28 (ddt, *J* = 14.7, 2.8, 1.3 Hz, 1H, trans), 2.68 (dd, *J* = 14.7, 11.1 Hz, 1H, trans), 3.36 – 3.43 (m, 1H, trans), 3.48 – 3.52 (m, 1H, enol), 3.55 (dd, *J* = 25.3, 1.5 Hz, 1H, trans), 3.58 – 3.65 (m, 1H, enol), 3.69 – 3.76 (m, 1H, enol), 3.95 – 4.03 (m, 2H, enol), 4.05 – 4.15 (m, 2H, trans), 4.15 – 4.22 (m, 2H, trans), 5.25 (d, *J* = 6.7 Hz, 1H, enol), 5.74 (d, *J* = 15.6 Hz, 1H, trans), 7.19 – 7.36 (m, 9H, 5H trans + 5H enol), 11.10 (d, *J* = 1.1 Hz, 1H, enol). ^{13}C NMR (176 MHz, Chloroform-*d*) δ 9.41 (enol), 9.54 (trans), 15.69 (d, *J* = 7.1 Hz, enol), 16.27 (d, *J* = 6.5 Hz, enol), 16.33 (d, *J* = 6.2 Hz, trans), 16.50 (d, *J* = 6.3 Hz, trans), 28.54 (enol), 29.07 (trans), 34.25 (d, *J* = 12.0 Hz, enol), 46.42 (trans), 53.61 (d, *J* = 126.5 Hz, trans), 62.04 (d, *J* = 6.0 Hz, enol), 62.11 (d, *J* = 4.8 Hz, enol), 62.88 (d, *J* = 6.5 Hz, trans), 63.34 (d, *J* = 6.5 Hz, trans), 67.83 (enol), 71.74 (trans), 74.01 (d, *J* = 13.9 Hz, enol), 75.03 (d, *J* = 4.2 Hz, trans), 90.46 (d, *J* = 181.1 Hz, enol), 127.75 (trans, 2×C), 127.82 (enol, 2×C), 128.09 (enol), 128.36 (trans), 128.73 (trans, 2×C), 129.42 (enol, 2×C), 138.94 (d, *J* = 14.6 Hz, trans), 140.07 (enol), 168.94 (d, *J* = 4.3 Hz, enol), 202.64 (d, *J* = 4.3 Hz, trans). EI-MS $[M]^+$ = 363.1. Anal. Calcd for $C_{17}H_{25}O_5P$: C, 59.99; H, 7.40. Found: C, 59.87; H, 7.42.

Due to overlap of signals, the signals of cis and enol forms in the ^{1}H NMR and ^{13}C NMR spectra have not been assigned.

2-Butyl-3-diethoxyphosphoryl-6-isopropyltetrahydropyran-4-one (**12c**) (254 mg, 76%) Colourless oil. ^{31}P NMR (284 MHz, Chloroform-*d*) δ 20.14 (trans), 20.18 (cis), 25.18 (enol). ^{1}H NMR (700 MHz, Chloroform-*d*) δ 0.73 – 1.71 (m, 21H trans + 22H cis + 22H enol), 1.77 (h, *J* = 6.7 Hz, 1H, trans), 2.14 (dd, *J* = 6.5, 1.8 Hz, 2H, enol), 2.28 (dd, *J* = 14.1, 10.0 Hz, 1H, cis), 2.34 (ddd, *J* = 14.0, 3.1, 1.5 Hz, 1H, trans), 2.48 (dt, *J* = 14.0, 4.0 Hz, 1H, cis), 2.70 (dd, *J*

= 14.0, 11.2 Hz, 1H, trans), 2.80 (dt, J = 23.9, 1.6 Hz, 1H, trans), 3.33 (dd, J = 21.1, 5.5 Hz, 1H, cis), 3.38 – 3.42 (m, 1H, enol), 3.50 (ddd, J = 11.3, 6.5, 3.1 Hz, 1H, trans), 3.57 (ddd, J = 10.2, 6.9, 3.5 Hz, 1H, cis), 3.96 – 4.25 (m, 4H trans + 4H cis + 4H enol), 4.59 (dddd, J = 14.7, 10.2, 4.6, 1.7 Hz, 1H, trans), 10.95 (d, J = 1.2 Hz, 1H, enol). ^{13}C NMR (176 MHz, Chloroform-*d*) δ 14.05 (trans), 14.10 (enol), 16.24 (d, J = 7.1 Hz, enol), 16.30 (d, J = 6.8 Hz, enol), 16.38 (d, J = 6.2 Hz, trans), 16.50 (d, J = 6.2 Hz, trans), 18.09 (trans), 18.29 (cis), 18.31 (enol), 18.32 (cis), 18.37 (trans), 18.96 (enol), 22.16 (cis), 22.27 (trans), 22.41 (enol), 27.21 (cis), 27.52 (trans), 28.18 (enol), 29.03 (cis), 32.73 (d, J = 12.0 Hz, enol), 32.85 (enol), 32.99 (cis), 33.13 (d, J = 13.7 Hz, trans), 33.30 (cis), 33.49 (trans), 44.08 (trans), 45.56 (d, J = 4.8 Hz, cis), 55.35 (d, J = 142.7 Hz, cis), 56.37 (d, J = 125.8 Hz, trans), 61.78 (d, J = 4.7 Hz, enol), 62.14 (d, J = 4.8 Hz, enol), 62.25 (d, J = 6.4 Hz, cis), 62.33 (d, J = 6.5 Hz, cis), 62.76 (d, J = 6.4 Hz, trans), 63.00 (d, J = 6.5 Hz, trans), 71.37 (enol), 71.98 (d, J = 14.1 Hz, enol), 73.70 (d, J = 4.5 Hz, trans), 74.78 (cis), 74.98 (trans), 75.24 (cis), 92.62 (d, J = 176.2 Hz, enol), 168.27 (d, J = 3.9 Hz, enol), 203.21 (d, J = 3.9 Hz, trans). ESI-MS [M+Na]$^+$ = 357.1. Anal. Calcd for $C_{16}H_{31}O_5P$: C, 57.47; H, 9.34. Found: C, 57.71; H, 9.31.

Due to overlap of signals, the signals of cis and enol forms in the ^{1}H NMR and ^{13}C NMR spectra have not been assigned.

3-Diethoxyphosphoryl-6-isopropyl-2-phenyltetrahydropyran-4-one (**12d**) (344 mg, 97%) Colourless oil. ^{31}P NMR (284 MHz, Chloroform-*d*) δ 18.38 (cis), 20.33 (trans), 23.05 (enol). ^{1}H NMR (700 MHz, Chloroform-*d*) δ 0.68 (d, J = 6.6 Hz, 3H, enol), 0.70 (d, J = 6.8 Hz, 3H, enol), 0.76 (d, J = 6.8 Hz, 3H, trans), 0.80 – 0.86 (m, 3H trans + 3H enol), 1.21 – 1.33 (m, 6H trans + 3H enol), 1.48 – 1.57 (m, J = 6.8 Hz, 1H, enol), 1.63 – 1.76 (m, 1H, trans), 2.16 (ddd, J = 17.9, 4.4, 1.6 Hz, 1H, enol), 2.21 (ddt, J = 17.9, 10.2, 1.5 Hz, 1H, enol), 2.28 (ddt, J = 14.5, 2.6, 1.2 Hz, 1H, trans), 2.72 (dd, J = 14.6, 11.4 Hz, 1H, trans), 3.16 (ddd, J = 11.3, 6.4, 2.8 Hz, 1H, trans), 3.20 (ddd, J = 10.2, 7.3, 4.4 Hz, 1H, enol), 3.55 (dd, J = 25.4, 1.4 Hz, 1H, trans), 3.62 – 3.69 (m, 1H, enol), 3.71 – 3.78 (m, 1H, enol), 3.95 – 4.04 (m, 2H, enol), 4.04 – 4.15 (m, 2H, trans), 4.15 – 4.23 (m, 2H, trans), 5.27 (d, J = 6.9 Hz, 1H, enol), 5.75 (d, J = 16.0 Hz, 1H, trans), 7.16 – 7.34 (m, 5H trans + 5H enol), 11.10 (d, J = 1.1 Hz, 1H, enol). ^{13}C NMR (176 MHz, Chloroform-*d*) δ 15.74 (d, J = 7.1 Hz, enol), 16.28 (d, J = 6.8 Hz, enol), 16.35 (d, J = 6.2 Hz, trans), 16.51 (d, J = 6.3 Hz, trans), 17.74 (enol), 17.97 (trans), 18.20 (trans), 18.42 (enol), 32.25 (d, J = 11.8 Hz, enol), 32.79 (enol), 33.15 (trans), 44.26 (trans), 53.47 (d, J = 126.5 Hz, trans), 62.07 (d, J = 5.7 Hz, enol), 62.12 (d, J = 4.6 Hz, enol), 62.94 (d, J = 6.6 Hz, trans), 63.27 (d, J = 6.7 Hz, trans), 71.35 (enol), 74.06 (d, J = 13.8 Hz, enol), 74.96 (d, J = 4.0 Hz, trans), 75.14 (trans), 90.38 (d, J = 181.7 Hz, enol), 127.73 (enol, 2×C), 127.81 (trans, 2×C), 128.04 (enol), 128.35 (trans), 128.70 (trans, 2×C), 129.49 (enol, 2×C), 138.92 (d, J = 14.6 Hz, trans), 139.98 (enol), 169.35 (d, J = 4.5 Hz, enol), 203.13 (d, J = 4.3 Hz, trans). ESI-MS [M+Na]$^+$ = 377.1. Anal. Calcd for $C_{18}H_{27}O_5P$: C, 61.01; H, 7.68. Found: C, 61.13; H, 7.68.

Due to overlap of signals, the signals of cis and enol forms in the ^{1}H NMR and ^{13}C NMR spectra have not been assigned.

2-Butyl-3-diethoxyphosphoryl-6-phenyltetrahydropyran-4-one (**12e**) (324 mg, 88%) Colourless oil. ^{31}P NMR (283 MHz, Chloroform-*d*) δ 19.63 (cis), 19.93 (trans), 24.92 (enol). ^{1}H NMR (700 MHz, Chloroform-*d*) δ 0.77 – 0.96 (m, 3H trans + 3H cis + 3H enol), 1.11 – 2.04 (m, 12H trans + 12H cis + 12H enol), 2.46 – 2.51 (m, 1H, enol), 2.54 (dddd, J = 17.6, 9.8, 2.2, 1.2 Hz, 1H, enol), 2.59 (ddt, J = 14.4, 3.0, 1.3 Hz, 1H, trans), 2.75 (ddt, J = 14.5, 7.7, 1.0 Hz, 1H, cis), 2.93 (dt, J = 23.7, 1.5 Hz, 1H, trans), 3.11 (dd, J = 14.4, 11.2 Hz, 1H, trans), 3.34 (ddd, J = 21.6, 4.9, 1.1 Hz, 1H, cis), 3.95 – 4.26 (m, 4H trans + 4H cis + 4H enol), 4.34 (dt, J = 10.9,

4.4 Hz, 1H, enol), 4.75 (dddd, *J* = 14.9, 9.7, 5.5, 1.4 Hz, 1H, trans), 4.87 (dd, *J* = 11.2, 3.4 Hz, 1H, trans), 5.12 (dd, *J* = 7.7, 4.9 Hz, 1H, cis), 7.27 – 7.44 (m, 5H trans + 5H cis + 5H enol), 11.08 (d, *J* = 1.2 Hz, 1H, enol). ^{13}C NMR (176 MHz, Chloroform-*d*) δ 14.00 (trans), 14.06 (enol), 16.26 (d, *J* = 6.9 Hz, enol), 16.28 (d, *J* = 6.9 Hz, enol), 16.41 (d, *J* = 6.0 Hz, trans), 16.50 (d, *J* = 6.0 Hz, trans), 22.20 (cis), 22.29 (trans), 22.46 (enol), 27.54 (trans), 28.13 (enol), 30.29 (cis), 33.00 (d, *J* = 14.0 Hz, trans), 33.19 (cis), 35.54 (d, *J* = 12.1 Hz, enol), 47.23 (d, *J* = 3.1 Hz, cis), 47.96 (trans), 56.20 (d, *J* = 126.1 Hz, trans), 61.89 (d, *J* = 5.0 Hz, enol), 62.20 (d, *J* = 5.0 Hz, enol), 62.35 (d, *J* = 6.7 Hz, cis), 62.55 (d, *J* = 6.4 Hz, cis), 62.96 (d, *J* = 6.5 Hz, trans), 63.07 (d, *J* = 6.8 Hz, trans), 68.25 (enol), 72.21 (trans), 72.30 (cis), 72.90 (enol), 74.20 (cis), 74.49 (d, *J* = 5.0 Hz, trans), 92.74 (d, *J* = 176.7 Hz, enol), 126.06 (cis, 2×C), 126.09 (trans, 2×C), 126.56 (enol, 2×C), 127.83 (enol), 128.25 (trans), 128.28 (cis), 128.60 (enol, 2×C), 128.74 (trans, 2×C), 140.09 (cis), 140.89 (trans), 141.28 (enol), 167.78 (d, *J* = 3.9 Hz, enol), 201.86 (d, *J* = 3.5 Hz, trans), 202.05 (d, *J* = 5.1 Hz, cis). ESI-MS [M+Na]$^+$ = 391.1. Anal. Calcd for $C_{19}H_{29}O_5P$: C, 61.94; H, 7.93. Found: C, 62.07; H, 7.96.

Due to overlap of signals, the signals of cis and enol forms in the ^{1}H NMR and ^{13}C NMR spectra have not been assigned.

3-diethoxyphosphoryl-2,6-diphenyltetrahydropyran-4-one (**12f**) (346 mg, 89%) Colourless oil.^{31}P NMR (284 MHz, Chloroform-*d*) δ 17.88 (cis), 20.01 (trans), 22.80 (enol). ^{1}H NMR (700 MHz, Chloroform-*d*) δ 0.87 (t, *J* = 7.0 Hz, 3H, enol), 1.32 (t, *J* = 7.1 Hz, 3H, enol), 1.34 – 1.39 (m, 6H, trans), 2.60 – 2.71 (m, 1H trans + 2H enol), 3.18 (dd, *J* = 14.9, 11.4 Hz, 1H, trans), 3.65 – 3.70 (m, 1H, enol), 3.73 (dt, *J* = 25.3, 1.4 Hz, 1H, trans), 3.77 – 3.85 (m, 1H, enol), 3.96 – 4.10 (m, 2H, enol), 4.18 – 4.26 (m, 2H, trans), 4.28 (dq, *J* = 8.1, 7.1 Hz, 2H, trans), 4.61 (dd, *J* = 11.4, 3.2 Hz, 1H, trans), 4.81 (dd, *J* = 9.3, 4.9 Hz, 1H, enol), 5.42 (d, *J* = 6.3 Hz, 1H, enol), 5.97 (dd, *J* = 15.8, 1.3 Hz, 1H, trans), 7.21 – 7.48 (m, 10H trans + 10H enol), 11.28 (d, *J* = 1.2 Hz, 1H, enol). ^{13}C NMR (176 MHz, Chloroform-*d*) δ 15.68 (d, *J* = 7.2 Hz, enol), 16.27 (d, *J* = 6.8 Hz, enol), 16.39 (d, *J* = 6.2 Hz, trans), 16.53 (d, *J* = 6.2 Hz, trans), 35.09 (d, *J* = 12.0 Hz, enol), 47.97 (trans), 53.56 (d, *J* = 126.5 Hz, trans), 62.10 (d, *J* = 5.8 Hz, enol), 62.17 (d, *J* = 5.0 Hz, enol), 63.13 (d, *J* = 6.8 Hz, trans), 63.38 (d, *J* = 6.6 Hz, trans), 68.42 (enol), 72.29 (trans), 74.37 (d, *J* = 13.7 Hz, enol), 75.52 (d, *J* = 4.3 Hz, trans), 90.61 (d, *J* = 180.8 Hz, enol), 126.05 (trans, 2×C), 126.12 (enol, 2×C), 127.83 (enol), 127.86 (trans, 2×C), 128.01 (enol, 2×C), 128.17 (trans), 128.35 (enol), 128.50 (trans), 128.65 (enol, 2×C), 128.67 (trans, 2×C), 128.92 (trans, 2×C), 129.46 (enol, 2×C), 138.39 (d, *J* = 14.6 Hz, trans), 139.83 (enol), 140.64 (trans), 140.97 (enol), 168.54 (d, *J* = 4.1 Hz, enol), 201.96 (d, *J* = 4.2 Hz, trans). ESI-MS [M+Na]$^+$ = 411.1. Anal. Calcd for $C_{21}H_{25}O_5P$: C, 64.94; H, 6.49. Found: C, 64.78; H, 6.47.

Due to overlap of signals, the signals of cis and enol forms in the ^{1}H NMR and ^{13}C NMR spectra have not been assigned.

2-Butyl-3-diethoxyphosphoryl-6-(4-methoxyphenyl)tetrahydropyran-4-one (**12g**) (291 mg, 73%) Colourless oil. ^{31}P NMR (101 MHz, Chloroform-*d*) δ 20.00 (cis), 20.35 (trans), 25.34 (enol). ^{1}H NMR (700 MHz, Chloroform-*d*) δ 0.85 – 0.93 (m, 3H trans + 3H cis + 3H enol), 1.22 – 1.89 (m, 12H trans + 12H cis + 12H enol), 2.43 – 2.48 (m, 1H, enol), 2.51 – 2.59 (m, 2H, trans + enol), 2.77 (dd, *J* = 14.3, 7.2 Hz, 1H, cis), 2.93 (dt, *J* = 23.6, 1.5 Hz, 1H, trans), 3.13 (dd, *J* = 14.3, 11.2 Hz, 1H, trans), 3.31 (ddd, *J* = 21.6, 4.8, 1.1 Hz, 1H, cis), 3.80 (s, 3H, cis), 3.80 (s, 3H, enol), 3.81 (s, 3H, trans), 3.97 – 4.27 (m, 4H trans + 4H cis + 4H enol), 4.30 – 4.34 (m, 1H, enol), 4.72 (dddd, *J* = 14.7, 9.7, 5.5, 1.4 Hz, 1H, trans), 4.82 (dd, *J* = 11.2, 3.4 Hz, 1H, trans), 5.11 (dd, *J* = 7.2, 5.1 Hz, 1H, cis), 6.88 – 6.93 (m, 2H trans + 2H cis + 2H enol), 7.27 – 7.29 (m, 2H, cis), 7.29 – 7.32 (m, 2H, enol), 7.33 – 7.38 (m, 2H, trans), 11.08 (s, 1H, enol). ^{13}C

NMR (176 MHz, Chloroform-*d*) δ 14.07 (trans), 14.14 (enol), 16.31 (d, *J* = 3.6 Hz, enol), 16.35 (d, *J* = 3.0 Hz, enol), 16.48 (d, *J* = 6.2 Hz, trans), 16.59 (d, *J* = 6.0 Hz, trans), 22.36 (trans), 22.53 (enol), 27.63 (trans), 28.20 (enol), 33.05 (d, *J* = 14.2 Hz, trans), 33.26 (enol), 35.52 (d, *J* = 12.5 Hz, enol), 48.05 (trans), 55.45 (enol), 55.47 (trans), 56.27 (d, *J* = 126.4 Hz, trans), 61.94 (d, *J* = 4.8 Hz, enol), 62.25 (d, *J* = 4.8 Hz, enol), 63.01 (d, *J* = 6.4 Hz, trans), 63.11 (d, *J* = 6.7 Hz, trans), 68.00 (enol), 72.01 (trans), 72.27 (d, *J* = 14.1 Hz, enol), 74.46 (d, *J* = 5.0 Hz, trans), 114.06 (enol, 2×C), 114.18 (trans, 2×C), 127.53 (enol, 2×C) 127.59 (trans, 2×C), 133.09 (trans), 133.43 (enol), 159.37 (enol), 159.67 (trans), 202.16 (d, *J* = 3.2 Hz, trans). ESI-MS $[M+Na]^+$ = 421.1. Anal. Calcd for $C_{20}H_{31}O_6P$: C, 60.29; H, 7.84. Found: C, 60.26; H, 7.85
Due to overlap of signals, the signals of cis and enol forms in the ^{1}H NMR and ^{13}C NMR spectra have not been assigned.

3-Diethoxyphosphoryl-6-(4-methoxyphenyl)-2-phenyltetrahydropyran-4-one (**12h**) (339 mg, 81%) Colourless oil. ^{31}P NMR (101 MHz, Chloroform-*d*) δ 18.18 (cis), 20.40 (trans), 23.17 (enol). ^{1}H NMR (700 MHz, Chloroform-*d*) δ 0.87 (t, *J* = 7.0 Hz, 3H, enol), 1.32 (t, *J* = 7.0 Hz, 3H, enol), 1.34 – 1.39 (m, 6H, trans), 2.56 – 2.62 (m, 1H trans + 1H enol), 2.68 (ddt, *J* = 17.9, 9.8, 1.5 Hz, 1H, enol), 3.19 (dd, *J* = 14.9, 11.3 Hz, 1H, trans), 3.64 – 3.68 (m, 1H, enol), 3.71 (dt, *J* = 25.1, 1.3 Hz, 1H, trans), 3.76 (s, 3H, enol), 3.78 (s, 3H, trans), 3.79 – 3.83 (m, 1H, enol), 3.96 – 4.09 (m, 2H, enol), 4.18 – 4.25 (m, 2H, trans), 4.25 – 4.31 (m, 2H, trans), 4.55 (dd, *J* = 11.3, 3.1 Hz, 1H, trans), 4.75 (dd, *J* = 9.7, 4.4 Hz, 1H, enol), 5.37 (d, *J* = 6.3 Hz, 1H, enol), 5.92 (dd, *J* = 15.7, 1.3 Hz, 1H, trans), 6.81 – 6.84 (m, 2H, enol), 6.85 – 6.88 (m, 2H, trans), 7.13 – 7.20 (m, 2H, enol), 7.25 (d, *J* = 8.6 Hz, 2H, trans), 7.28 – 7.47 (m, 5H trans + 5H enol), 11.27 (d, *J* = 1.1 Hz, 1H, enol). ^{13}C NMR (176 MHz, Chloroform-*d*) δ 15.73 (d, *J* = 7.4 Hz, enol), 16.31 (d, *J* = 6.8 Hz, enol), 16.44 (d, *J* = 6.2 Hz, trans), 16.59 (d, *J* = 6.2 Hz, trans), 35.03 (d, *J* = 12.0 Hz, enol), 47.99 (trans), 53.59 (d, *J* = 126.6 Hz, trans), 55.40 (enol), 55.43 (trans), 62.14 (d, *J* = 5.8 Hz, enol), 62.21 (d, *J* = 5.0 Hz, enol), 63.16 (d, *J* = 6.7 Hz, trans), 63.42 (d, *J* = 6.6 Hz, trans), 68.19 (enol), 72.10 (trans), 74.37 (d, *J* = 13.8 Hz, enol), 75.47 (d, *J* = 4.3 Hz, trans), 90.65 (d, *J* = 180.8 Hz, enol), 113.95 (enol, 2×C), 114.11 (trans, 2×C), 127.57 (enol, 2×C), 127.63 (trans, 2×C), 127.89 (trans, 2×C), 128.03 (enol, 2×C), 128.35 (enol), 128.64 (trans), 128.95 (trans, 2×C), 129.49 (enol, 2×C), 132.79 (trans), 133.06 (enol), 138.56 (d, *J* = 14.6 Hz, trans), 139.98 (enol), 159.36 (enol), 159.61 (trans), 168.69 (d, *J* = 4.4 Hz, enol), 202.24 (d, *J* = 4.0 Hz, trans). ESI-MS $[M+Na]^+$ = 441.0. Anal. Calcd for $C_{22}H_{27}O_6P$: C, 63.15; H, 6.50. Found: C, 63.31; H, 6.52.

Due to overlap of signals, the signals of cis and enol forms in the ^{1}H NMR and ^{13}C NMR spectra have not been assigned.

2-Butyl-3-diethoxyphosphoryl-6-ferrocenyltetrahydropyran-4-one (**12i**) (376 mg, 79%) Red oil. ^{31}P NMR (284 MHz, Chloroform-*d*) δ 19.43 (cis), 19.80 (trans), 25.09 (enol). ^{1}H NMR (700 MHz, Chloroform-*d*) δ 0.74 – 0.98 (m, 3H trans + 3H cis + 3H enol), 1.09 – 1.88 (m, 12H trans + 12H cis + 12H enol), 2.53 (dd, *J* = 17.5, 4.4 Hz, 1H, enol), 2.58 (dd, *J* = 17.6, 9.0 Hz, 1H, enol), 2.71 (dd, *J* = 14.3, 3.8 Hz, 1H, trans), 2.79 (dd, *J* = 14.3, 4.3 Hz, 1H, cis), 2.87 (dt, *J* = 24.0, 2.0 Hz, 1H, trans), 3.09 (dd, *J* = 14.3, 10.8 Hz, 1H, trans), 3.88 – 4.31 (m, 13H trans + 13H cis + 13H enol), 4.55 – 4.62 (m, 1H, trans), 4.68 (dt, *J* = 9.2, 4.6 Hz, 1H, cis), 4.71 (dd, *J* = 10.8, 3.9 Hz, 1H, trans), 5.16 (dd, *J* = 6.5, 4.3 Hz, 1H, cis), 11.11 (s, 1H, enol). ^{13}C NMR (176 MHz, Chloroform-*d*) δ 14.00 (cis), 14.05 (trans), 14.14 (enol), 16.24 (d, *J* = 6.8 Hz, 2×C, cis + enol), 16.36 (d, *J* = 6.3 Hz, trans), 16.47 (d, *J* = 6.4 Hz, cis), 16.52 (d, *J* = 6.0 Hz, trans), 22.16 (cis), 22.24 (trans), 22.46 (enol), 27.56 (trans), 27.88 (cis), 28.05 (enol), 31.64 (d, *J* = 2.6 Hz, cis), 33.20 (enol), 33.38 (d, *J* = 12.0 Hz, trans), 34.08 (d, *J* = 12.1 Hz, enol), 45.12 (cis), 46.19 (trans), 56.39 (d, *J* = 125.4 Hz, trans), 57.13 (d, *J* = 132.1 Hz, cis), 61.82 (d, *J* = 4.7 Hz,

enol), 62.13 (d, *J* = 4.6 Hz, enol), 62.25 (d, *J* = 6.6 Hz, cis), 62.53 (d, *J* = 6.6 Hz, cis), 62.73 (d, *J* = 6.5 Hz, trans), 63.08 (d, *J* = 6.5 Hz, trans), 65.43 (enol), 66.01 (trans), 67.38 (cis), 67.66 (trans), 68.03 (enol), 68.09 (enol), 68.13 (cis), 68.20 (trans), 68.42 (enol), 68.49 (cis), 68.58 (trans), 68.71 (enol, 5×C), 68.77 (trans, 5×C), 68.81 (cis, 5×C), 69.13 (cis), 69.33 (trans), 71.54 (d, *J* = 14.1 Hz, enol), 71.63 (cis), 72.03 (d, *J* = 3.9 Hz, cis), 73.47 (d, *J* = 3.7 Hz, trans), 86.68 (cis), 87.56 (enol), 87.63 (trans), 92.69 (d, *J* = 176.1 Hz, enol), 167.90 (d, *J* = 4.2 Hz, enol), 202.37 (d, *J* = 4.3 Hz, trans), 202.56 (d, *J* = 3.8 Hz, cis). ESI-MS $[M+Na]^+$ = 499.1. Anal. Calcd for $C_{23}H_{33}FeO_5P$: C, 58.00; H, 6.98. Found: C, 57.87; H, 7.00.

Due to overlap of signals, the signals of cis and enol forms in the ^{1}H NMR and ^{13}C NMR spectra have not been assigned.

3-diethoxyphosphoryl-6-ferrocenyl-2-phenyltetrahydropyran-4-one (**12j**) (313 mg, 63%) Red oil. ^{31}P NMR (284 MHz, Chloroform-*d*) δ 17.71 (cis), 19.84 (trans), 23.00 (enol). ^{1}H NMR (700 MHz, Chloroform-*d*) δ 0.86 (t, *J* = 7.0 Hz, 3H, enol), 0.97 (t, *J* = 7.1 Hz, 3H, cis), 1.20 (t, *J* = 7.0 Hz, 3H, cis), 1.27 – 1.36 (m, 6H trans + 3H enol), 2.62 (d, *J* = 6.8 Hz, 2H, enol), 2.71 (dd, *J* = 14.8, 3.5 Hz, 1H, trans), 2.97 (d, *J* = 14.4 Hz, 1H, cis), 3.12 (dd, *J* = 14.8, 10.8 Hz, 1H, trans), 3.61 (dd, *J* = 24.5, 1.9 Hz, 1H, trans), 3.62 – 3.68 (m, 1H, enol), 3.78 (dp, *J* = 10.0, 7.0 Hz, 1H, enol), 3.97 (s, 4H, enol), 3.99 – 4.33 (m, 13H trans + 7H enol), 4.46 (dd, *J* = 10.7, 3.6 Hz, 1H, trans), 4.62 (t, *J* = 6.8 Hz, 1H, enol), 5.27 (d, *J* = 5.9 Hz, 1H, enol), 5.80 (dd, *J* = 15.1, 2.3 Hz, 1H, trans), 7.27 – 7.49 (m, 5H trans + 5H enol), 11.28 (d, *J* = 1.2 Hz, 1H, enol). ^{13}C NMR (176 MHz, Chloroform-*d*) δ 15.66 (d, *J* = 7.3 Hz, enol), 16.25 (d, *J* = 6.5 Hz, enol), 16.31 (d, *J* = 6.2 Hz, trans), 16.52 (d, *J* = 6.1 Hz, trans), 34.83 (d, *J* = 11.8 Hz, enol), 47.02 (trans), 53.95 (d, *J* = 126.4 Hz, trans), 61.99 (d, *J* = 5.7 Hz, enol), 62.12 (d, *J* = 4.6 Hz, enol), 62.89 (d, *J* = 6.6 Hz, trans), 63.35 (d, *J* = 6.5 Hz, trans), 65.83 (enol), 65.92 (trans), 66.07 (enol), 67.38 (trans), 67.45 (enol), 67.81 (enol), 68.15 (trans), 68.20 (enol), 68.36 (trans), 68.69 (enol, 5×C), 68.78 (trans, 5×C), 69.49 (trans), 73.88 (d, *J* = 13.7 Hz, enol), 75.03 (d, *J* = 3.4 Hz, trans), 87.57 (trans), 90.67 (d, *J* = 180.0 Hz, enol), 127.87 (trans, 2×C), 127.93 (enol, 2×C), 128.26 (enol), 128.59 (trans), 128.79 (trans, 2×C), 129.29 (enol, 2×C), 138.86 (d, *J* = 13.2 Hz, trans), 140.03 (enol), 168.66 (d, *J* = 4.4 Hz, enol), 202.33 (d, *J* = 4.6 Hz, trans). ESI-MS $[M+Na]^+$ = 519.0. Anal. Calcd for $C_{25}H_{29}FeO_5P$: C, 60.50; H, 5.89. Found: C, 60.57; H, 5.90.

Due to overlap of signals, the signals of cis and enol forms in the ^{1}H NMR and ^{13}C NMR spectra have not been assigned.

1.4 Synthesis of 2-isopropyl-6-alkyl(aryl)-3-diethoxyphosphoryltetrahydropyran-4-ones 12k-o.

The solution of 6-alkyl(aryl)-3-diethoxyphosphoryldihydropyran-4-one **11a-e** (1.0 mmol) in dry THF (10 mL) was cooled to 0°C in ice – water bath and isopropylmagnesium chloride (3.0 mmol in THF) was added dropwise in argon atmosphere. After 2 hours at this temperature, saturated solution of ammonium chloride (15 mL) was added. The water layer was washed with DCM (3 x15 mL). Combined organic extracts were washed with brine (20 mL) and dried over $MgSO_4$. The solvents were evaporated under reduced pressure and the resulting crude product was purified by column chromatography (eluent DCM : Acetone 20:1).

The following abbreviations are used to simplify the analysis of the spectra:
trans 1 = r-2-trans-3-trans-6
enol 1 = trans-enol
cis 1 = r-2-cis-3-trans-6

trans 2 = r-2-trans-3-cis-6
enol 2 = cis-enol
cis 2 = r-2-cis-3-cis-6

3-Diethoxyphosphoryl-6-ethyl-2-isopropyltetrahydropyran-4-one (**12k**) (193 mg, 63%) Colourless oil. ^{31}P NMR (284 MHz, Chloroform-*d*) δ 18.73 (cis 2), 19.09 (cis 1), 20.52 (trans 1), 20.64 (trans 2), 25.35 (enol 1), 25.54 (enol 2). ^{1}H NMR (700 MHz, Chloroform-*d*) δ 0.86 – 1.03 (m, 9H trans 1 + 9H trans 2 + 9H cis 2), 1.25 – 1.33 (m, 6H trans 1 + 6H trans 2 + 6H cis 2), 1.41 – 1.94 (m, 3H trans 1 + 3H trans 2 + 3H cis 2), 2.03 (ddd, *J* = 16.8, 3.2, 1.6 Hz, 1H, enol 2), 2.05 – 2.12 (m, 1H, enol 2), 2.17 (ddt, *J* = 16.6, 9.9, 1.6 Hz, 1H, trans 2), 2.29 – 2.41 (m, 2H, trans 1 + cis 2), 2.64 (dd, *J* = 16.6, 4.7 Hz, 1H, trans 2), 2.66 – 2.69 (m, 1H, trans 1), 2.98 (ddd, *J* = 24.3, 7.2, 1.7 Hz, 1H, trans 2), 3.05 (ddd, *J* = 39.1, 10.3, 3.0 Hz, 1H, cis 2), 3.05 – 3.13 (m, 1H, trans 1), 3.16 (ddd, *J* = 21.0, 2.9, 1.6 Hz, 1H, cis 2), 3.33 (tdd, *J* = 10.4, 5.2, 3.2 Hz, 1H, enol 2), 3.38 – 3.43 (m, 1H, cis 2), 3.65 – 3.71 (m, 1H, cis 2), 3.85 (ddt, *J* = 9.8, 7.6, 4.8 Hz, 1H, trans 2), 4.00 (ddd, *J* = 11.7, 7.3, 3.2 Hz, 1H, trans 1), 4.02 – 4.20 (m, 5H trans 1 + 5H trans 2 + 4H cis 2), 11.13 (d, *J* = 1.1 Hz, 1H, enol 2), 11.16 (d, *J* = 1.2 Hz, 1H, enol 1). ^{13}C NMR (176 MHz, Chloroform-*d*) δ 9.56 (trans 2), 9.80 (trans 1), 9.99 (cis 2), 15.37 (trans 2), 16.28 (d, *J* = 6.4 Hz, cis 2), 16.36 (d, *J* = 6.1 Hz, trans 2), 16.42 (d, *J* = 6.3 Hz, trans 2), 16.48 (d, *J* = 6.3 Hz, trans 1), 18.26 (trans 1), 18.83 (cis 2), 19.40 (trans 2), 19.58 (cis 2), 20.35 (trans 1), 29.44 (cis 2), 29.45 (d, *J* = 13.3 Hz, trans 1), 29.57 (d, *J* = 3.5 Hz, trans 2), 31.48 (d, *J* = 3.9 Hz, cis 2), 33.10 (d, *J* = 3.4 Hz, trans 2), 35.00 (d, *J* = 12.3 Hz, enol 2), 46.22 (cis 2), 46.30 (trans 2), 46.70 (trans 1), 53.68 (d, *J* = 127.2 Hz, trans 2), 53.70 (d, *J* = 126.5 Hz, trans 1), 55.42 (d, *J* = 127.3 Hz, cis 2), 62.12 (d, *J* = 6.5 Hz, trans 1), 62.23 (d, *J* = 6.9 Hz, trans 2), 62.50 (d, *J* = 6.5 Hz, cis 2), 62.67 (d, *J* = 6.9 Hz, trans 1), 63.04 (d, *J* = 6.4 Hz, cis 2), 63.11 (d, *J* = 6.9 Hz, trans 2), 71.91 (trans 1), 76.00 (trans 2), 79.53 (cis 2), 79.89 (d, *J* = 4.7 Hz, trans 1), 80.27 (trans 2), 85.13 (d, *J* = 7.0 Hz, cis 2), 91.54 (d, *J* = 176.2 Hz, enol 2), 169.75 (d, *J* = 3.9 Hz, enol 2), 202.71 (d, *J* = 3.8 Hz, trans 1), 202.97 (d, *J* = 2.5 Hz, cis 2), 203.69 (d, *J* = 5.9 Hz, trans 2). ESI-MS $[M+Na]^+$ = 329.1. Anal. Calcd for $C_{14}H_{27}O_5P$: C, 54.89; H, 8.88. Found: C, 54.99; H, 8.85.

Due to overlap of signals, the signals of trans, cis and enol forms in the ^{1}H NMR and ^{13}C NMR spectra have not been assigned.

3-Diethoxyphosphoryl-2,6-diisopropyltetrahydropyran-4-one (**12l**) (218 mg, 68%) Colourless oil. ^{31}P NMR (284 MHz, Chloroform-*d*) δ 18.85 (cis 2), 19.20 (cis 1), 20.60 (trans 1), 20.61 (trans 2), 25.39 (enol 1), 25.56 (enol 2). ^{1}H NMR (700 MHz, Chloroform-*d*) δ 0.85 (d, *J* = 6.8 Hz, 3H, trans 2), 0.87 (d, *J* = 6.8 Hz, 3H, trans 2), 0.88 (d, *J* = 6.5 Hz, 3H, trans 1), 0.90 (d, *J* = 6.9 Hz, 3H, trans 1), 0.92 (d, *J* = 6.6 Hz, 3H, trans 2), 0.94 (d, *J* = 6.6 Hz, 3H, trans 1), 0.97 (d, *J* = 6.7 Hz, 3H, trans 1), 0.99 (d, *J* = 6.9 Hz, 3H, trans 2), 1.25 – 1.33 (m, 6H trans 1 + 6H trans 2+ 6H cis 2), 1.67 (dp, *J* = 13.5, 6.7 Hz, 1H, trans 1), 1.74 – 1.85 (m, 2H, trans 1 + trans 2), 1.90 (ddq, *J* = 10.1, 6.8, 3.4 Hz, 1H, trans 2), 2.24 (ddt, *J* = 16.5, 9.9, 1.6 Hz, 1H, trans 2), 2.32 (ddd, *J* = 14.3, 3.0, 1.6 Hz, 1H, trans 1), 2.62 (dd, *J* = 16.5, 4.8 Hz, 1H, trans 2), 2.67 (dd, *J* = 14.4, 11.6 Hz, 1H, cis 2), 2.71 (dd, *J* = 14.1, 11.2 Hz, 1H, trans 1), 2.97 (ddd, *J* = 24.3, 7.3, 1.8 Hz, 1H, trans 2), 3.04 (ddd, *J* = 39.4, 10.3, 2.9 Hz, 1H, cis 2), 3.08 (dt, *J* = 24.6, 1.4 Hz, 1H, trans 2), 3.48 (ddd, *J* = 11.2, 6.4, 3.0 Hz, 1H, trans 1), 3.62 (ddd, *J* = 9.9, 6.8, 4.8 Hz, 1H, trans 2), 3.97 (ddd, *J* = 11.7, 7.3, 3.2 Hz, 1H, trans 2), 4.09 (ddd, *J* = 15.3, 6.6, 1.5 Hz, 1H, trans 1), 4.12 – 4.20 (m, 4H trans 1 + 4H trans 2+ 4H cis 2), 11.14 (d, *J* = 1.2 Hz, 1H, enol 2), 11.16 (d, *J* = 1.2 Hz, 1H, enol 1). ^{13}C NMR (176 MHz, Chloroform-*d*) δ 15.34 (trans 2), 16.29 (d, *J* = 6.4 Hz, cis 2), 16.36 (d, *J* = 6.4 Hz, trans 1), 16.37 (d, *J* = 6.0 Hz, trans 2), 16.42 (d, *J* = 6.0 Hz, trans 2), 16.49 (d, *J* = 6.3 Hz, trans 1), 17.84 (trans 2), 18.02 (trans 2), 18.07 (trans 1), 18.34

(trans 1), 18.40 (cis 2), 18.41 (trans 2), 18.44 (cis 2), 18.85 (cis 2), 19.55 (trans 1), 19.65 (trans 1), 20.39 (cis 2), 29.38 (d, J = 13.4 Hz, trans 1), 31.57 (d, J = 3.9 Hz, cis 2), 33.20 (d, J = 3.5 Hz, trans 2), 33.71 (trans 1), 33.74 (trans 2), 33.80 (cis 2), 43.96 (trans 1), 44.27 (trans 2), 44.58 (cis 2), 53.67 (d, J = 126.5 Hz, trans 1), 53.74 (d, J = 127.2 Hz, trans 2), 55.42 (d, J = 127.3 Hz, cis 2), 62.15 (d, J = 6.5 Hz, cis 2), 62.24 (d, J = 6.9 Hz, trans 2), 62.44 (d, J = 6.8 Hz, cis 2), 62.71 (d, J = 6.8 Hz, trans 1), 62.96 (d, J = 6.5 Hz, trans 1), 63.08 (d, J = 6.9 Hz, trans 2), 75.42 (trans 1), 79.60 (trans 2), 79.92 (d, J = 5.0 Hz, trans 1), 80.27 (trans 2), 83.24 (cis 2), 85.14 (d, J = 7.0 Hz, cis 2), 203.23 (d, J = 3.7 Hz, trans 1), 203.44 (d, J = 2.1 Hz, cis 2), 204.00 (d, J = 6.2 Hz, trans 2). ESI-MS $[M+Na]^+$ = 343.1. Anal. Calcd for $C_{15}H_{29}O_5P$: C, 56.24; H, 9.12. Found: C, 56.17; H, 9.16.

Due to overlap of signals, the signals of trans, cis and enol forms in the ^{1}H NMR and ^{13}C NMR spectra have not been assigned.

3-Diethoxyphosphoryl-2-isopropyl-6-phenyltetrahydropyran-4-one (**12m**) (308 mg, 87%) Colourless oil. ^{31}P NMR (284 MHz, Chloroform-*d*) δ 18.64 (cis 2), 18.93 (cis 1), 20.41 (trans 1), 20.48 (trans 2), 25.08 (enol 1), 25.13 (enol 2). ^{1}H NMR (700 MHz, Chloroform-*d*) δ 0.77 (d, J = 6.9 Hz, 3H, trans 2), 0.87 (d, J = 6.6 Hz, 3H, trans 2), 0.89 (d, J = 7.0 Hz, 3H, cis 2), 0.93 (d, J = 6.5 Hz, 3H, trans 1), 0.97 (d, J = 7.0 Hz, 3H, cis 2), 0.98 (d, J = 6.7 Hz, 3H, trans 1), 1.26 – 1.36 (m, 6H trans 1 + 6H trans 2 + 6H cis 2), 1.97 (dp, J = 10.4, 6.5 Hz, 1H, trans 1), 2.02 (pd, J = 6.8, 3.1 Hz, 1H, cis 2), 2.16 (heptd, J = 6.9, 2.8 Hz, 1H, trans 2), 2.48 (ddt, J = 16.8, 10.4, 1.6 Hz, 1H, trans 2), 2.56 (ddt, J = 14.3, 2.8, 1.2 Hz, 1H, trans 1), 2.58 – 2.62 (m, 1H, cis 2), 2.94 (dd, J = 16.7, 4.7 Hz, 1H, trans 2), 3.01 (ddd, J = 14.1, 11.6, 0.6 Hz, 1H, cis 2), 3.08 (ddd, J = 24.4, 7.1, 1.6 Hz, 1H, trans 2), 3.12 (dd, J = 14.3, 11.3 Hz, 1H, trans 1), 3.19 (dt, J = 24.4, 1.3 Hz, 1H, trans 1), 3.32 – 3.38 (m, 2H, trans 2 + cis 2), 4.03 – 4.20 (m, 4H trans 1 + 4H trans 2 + 4H cis 2), 4.22 (ddd, J = 15.1, 10.4, 1.4 Hz, 1H, trans 1), 4.53 (dd, J = 8.2, 5.7 Hz, 1H, enol 1), 4.58 (dd, J = 11.6, 3.0 Hz, 1H, cis 2), 4.80 (dd, J = 11.3, 3.2 Hz, 1H, trans 1), 5.06 (dd, J = 10.4, 4.7 Hz, 1H, trans 2), 7.33 (m, 5H trans 1 + 5H trans 2 + 5H cis 2), 11.22 (s, 1H, enol 1), 11.30 (s, 1H, enol 2). ^{13}C NMR (176 MHz, Chloroform-*d*) δ 15.43 (cis 2), 16.13 (trans 2), 16.34 (d, J = 5.9 Hz, trans 1), 16.42 (d, J = 6.0 Hz, trans 1), 18.40 (trans 1), 18.78 (cis 2), 19.37 (trans 2), 19.45 (trans 1), 20.43 (cis 2), 29.38 (d, J = 13.5 Hz, trans 1), 31.43 (d, J = 3.8 Hz, cis 2), 31.66 (trans 2), 33.18 (d, J = 3.7 Hz, cis 2), 34.60 (trans 1), 47.81 (trans 1), 48.27 (trans 2), 48.40 (cis 2), 53.34 (d, J = 127.8 Hz, trans 2), 53.93 (d, J = 126.3 Hz, trans 1) , 55.25 (d, J = 127.8 Hz, cis 2), 62.28 (d, J = 7.2 Hz, trans 2), 62.49 (d, J = 6.5 Hz, cis 2), 62.85 (d, J = 6.6 Hz, trans 1), 62.98 (d, J = 6.6 Hz, trans 1), 63.22 (d, J = 6.9 Hz, trans 2), 71.34 (trans 2), 72.60 (trans 1), 75.82 (cis 2), 79.50 (cis 2), 80.26 (trans 2), 80.43 (d, J = 5.2 Hz, trans 1), 85.22 (d, J = 7.1 Hz, cis 2), 125.33 (trans 2, 2×C), 125.66 (cis 2, 2×C), 126.04 (trans 1, 2×C), 127.72 (trans 2), 127.93 (cis 2), 128.18 (trans 1), 128.50 (trans 2 + cis 2, 4×C), 128.65 (trans 1, 2×C), 140.74 (trans 2), 140.87 (cis 2), 141.53 (trans 2), 201.80 (d, J = 3.4 Hz, trans 1), 202.01 (d, J = 2.2 Hz, cis 2), 202.62 (d, J = 6.1 Hz, trans 2). ESI-MS $[M+Na]^+$ = 377.1. Anal. Calcd for $C_{18}H_{27}O_5P$: C, 61.01; H, 7.68. Found: C, 61.23; H, 7.71.

Due to overlap of signals, the signals of trans, cis and enol forms in the ^{1}H NMR and ^{13}C NMR spectra have not been assigned.

3-Diethoxyphosphoryl-2-isopropyl-6-(4-methoxyphenyl)tetrahydropyran-4-one (**12n**) (258 mg, 67%) Colourless oil. ^{31}P NMR (284 MHz, Chloroform-*d*) δ 18.75 (cis 2), 19.01 (cis 1), 20.49 (trans 1), 20.54 (trans 2), 25.16 (enol 2), 25.23 (enol 1). ^{1}H NMR (700 MHz, Chloroform-*d*) δ 0.95 (d, J = 6.5 Hz, 3H, trans 1), 0.97 (d, J = 6.8 Hz, 3H, cis 2), 0.98 (d, J = 6.6 Hz, 3H, cis 2), 1.00 (d, J = 6.5 Hz, 3H, trans 1), 1.05 (d, J = 6.0 Hz, 3H, trans 2), 1.06 (d, J = 5.8 Hz,

3H, trans 2), 1.29 – 1.38 (m, 6H trans 1 + 6H trans 2 + 6H cis 2), 1.98 (dp, *J* = 10.4, 6.5 Hz, 1H, trans 1), 2.02 – 2.06 (m, 1H, trans 2), 2.08 (dtd, *J* = 14.3, 7.2, 2.3 Hz, 1H, cis 2), 2.49 – 2.53 (m, 1H, cis 2), 2.54 (ddt, *J* = 14.3, 2.8, 1.2 Hz, 1H, trans 1), 2.57 – 2.61 (m, 1H, trans 2), 2.93 (dd, *J* = 16.7, 4.6 Hz, 1H, trans 2), 3.06 (ddd, *J* = 14.0, 11.6, 0.6 Hz, 1H, cis 2), 3.11 (ddd, *J* = 24.4, 7.1, 1.6 Hz, 1H, trans 2), 3.16 (dd, *J* = 14.3, 11.3 Hz, 1H, trans 1), 3.21 (dt, *J* = 24.3, 1.4 Hz, 1H, trans 1), 3.79 (s, 3H, trans 2), 3.80 (s, 3H, trans 1), 3.80 (s, 3H, cis 2), 4.14 (ddd, *J* = 16.5, 7.2, 1.5 Hz, 1H, trans 1), 4.17 – 4.26 (m, 4H trans 1 + 4H trans 2 + 4H cis 2) 4.50 (dd, *J* = 10.5, 3.3 Hz, 1H, enol 2), 4.54 (dd, *J* = 11.5, 2.9 Hz, 1H, cis 2), 4.77 (dd, *J* = 11.3, 3.2 Hz, 1H, trans 1), 5.02 (dd, *J* = 10.4, 4.7 Hz, 1H, trans 2), 6.88 – 6.93 (m, 2H trans 1 + 2H trans 2 + 2H cis 2), 7.22 – 7.25 (m, 2H, cis 2), 7.27 – 7.29 (m, 1H, trans 2), 7.31 – 7.34 (m, 1H, trans 2), 7.35 – 7.38 (m, 2H, trans 1), 11.24 (d, *J* = 1.1 Hz, 1H, enol 2), 11.32 (d, *J* = 1.1 Hz, 1H, enol 1). ^{13}C NMR (176 MHz, Chloroform-*d*) δ 15.43 (trans 2), 16.36 (d, *J* = 6.3 Hz, trans 1), 16.46 (d, *J* = 6.2 Hz, trans 1), 18.45 (trans 1), 18.79 (cis 2), 19.37 (trans 2), 19.47 (trans 1), 20.46 (cis 2), 29.39 (d, *J* = 13.8 Hz, trans 1), 31.45 (d, *J* = 3.7 Hz, cis 2), 33.12 (d, *J* = 3.7 Hz, trans 2), 47.85 (trans 1), 48.33 (trans 2), 48.39 (cis 2), 52.98 (d, *J* = 128.2 Hz, trans 2), 53.47 (cis 2), 53.59 (d, *J* = 127.1 Hz, trans 1), 54.91 (d, *J* = 127.4 Hz, cis 2), 55.30 (trans 2), 55.32 (trans 1), 62.29 (d, *J* = 6.8 Hz, trans 2), 62.54 (d, *J* = 6.6 Hz, cis 2), 62.88 (d, *J* = 6.8 Hz, trans 1), 63.01 (d, *J* = 6.7 Hz, trans 1), 63.24 (d, *J* = 6.9 Hz, trans 2), 72.37 (trans 1), 75.68 (cis 2), 79.33 (cis 2), 80.27 (trans 2), 80.37 (d, *J* = 5.2 Hz, trans 1), 85.20 (d, *J* = 7.0 Hz, cis 2), 113.91 (cis 2, 2×C), 113.93 (trans 2, 2×C), 114.06 (trans 1, 2×C), 126.78 (trans 2, 2×C), 127.12 (cis 2, 2×C), 127.50 (trans 1, 2×C), 132.91 (trans 1), 133.06 (cis 2), 133.69 (trans 2), 159.22 (trans 2), 159.38 (cis 2), 159.58 (trans 1), 202.05 (d, *J* = 3.4 Hz, trans 1), 202.25 (d, *J* = 2.2 Hz, cis 2), 202.92 (d, *J* = 5.9 Hz, trans 2). ESI-MS [M+Na]$^+$ = 407.1. Anal. Calcd for $C_{19}H_{29}O_6P$: C, 59.37; H, 7.60. Found: C, 59.23; H, 7.62.

Due to overlap of signals, the signals of trans, cis and enol forms in the ^{1}H NMR and ^{13}C NMR spectra have not been assigned.

3-Diethoxyphosphoryl-2-isopropyl-6-ferrocenyltetrahydropyran-4-one (**12o**) (273 mg, 59%) Red oil. ^{31}P NMR (284 MHz, Chloroform-*d*) δ 19.04 (cis 2), 20.28 (trans 1), 25.34 (enol 1). ^{1}H NMR (700 MHz, Chloroform-*d*) δ 0.92 (d, *J* = 6.6 Hz, 3H, trans 1), 1.03 (d, *J* = 6.5 Hz, 3H, trans 1), 1.30 – 1.38 (m, 6H, trans 1), 1.87 – 2.04 (m, 1H, trans 1), 2.75 (dd, *J* = 14.3, 3.4 Hz, 1H, trans 1), 3.14 (dd, *J* = 14.2, 10.9 Hz, 1H, trans 1), 3.17 (dd, *J* = 24.5, 1.6 Hz, 1H, trans 1), 3.34 (dd, *J* = 14.5, 8.3 Hz, 1H, cis 2), 4.02 – 4.21 (m, 13H, trans 1), 4.20 – 4.27 (m, 1H, trans 1), 4.69 (dd, *J* = 10.9, 3.5 Hz, 1H, trans 1), 11.34 (d, *J* = 1.2 Hz, 1H, enol 1). ^{13}C NMR (176 MHz, Chloroform-*d*) δ 16.38 (d, *J* = 6.3 Hz, cis 2), 16.45 (d, *J* = 6.2 Hz, trans 1), 16.54 (d, *J* = 6.7 Hz, cis 2), 16.60 (d, *J* = 5.9 Hz, trans 1), 18.13 (trans 1), 18.61 (cis 2), 19.71 (trans 1), 20.38 (cis 2), 29.71 (d, *J* = 12.3 Hz, trans 1), 30.88 (d, *J* = 3.7 Hz, cis 2), 43.18 (cis 2), 46.02 (trans 1), 53.81 (d, *J* = 126.0 Hz, trans 1), 55.79 (d, *J* = 126.5 Hz, cis 2), 62.27 (d, *J* = 6.6 Hz, cis 2), 62.73 (d, *J* = 6.7 Hz, cis 2), 62.83 (d, *J* = 6.6 Hz, trans 1), 63.21 (d, *J* = 6.4 Hz, trans 1), 66.05 (trans 1), 67.93 (trans 1), 68.15 (cis 2), 68.34 (trans 1), 68.71 (trans 1), 68.85 (trans 1, 4×C), 68.89 (cis 2, 4×C), 69.07 (cis 2), 69.34 (cis 2), 69.56 (cis 2), 69.60 (trans 1), 73.24 (cis 2), 76.66 (d, *J* = 7.0 Hz, cis 2), 79.56 (d, *J* = 4.3 Hz, trans 1), 86.02 (cis 2), 87.55 (trans 1), 202.67 (d, *J* = 4.3 Hz, trans 1), 203.10 (d, *J* = 2.3 Hz, cis 2). ESI-MS [M+Na]$^+$ = 485.1. Anal. Calcd for $C_{22}H_{31}FeO_5P$: C, 57.16; H, 6.76. Found: C, 57.11; H, 6.77.

Due to overlap of signals, the signals of trans, cis and enol forms in the ^{1}H NMR and ^{13}C NMR spectra have not been assigned.

1.5 Synthesis of 2-alkyl(aryl)-5-diethoxyphosphoryltetrahydropyran-4-ones 14a-d.

The solution of 6-alkyl(aryl)-3-diethoxyphosphoryldihydropyran-4-one **11a-d** (1.0 mmol) in dry THF (10 mL) was cooled to -78°C in dry ice - acetone bath and L-selectride® (1.1 mmol in THF) was added dropwise in argon atmosphere. After 1 hour at this temperature, the reaction was carried out at 0 °C for 1 hour. Reaction was completed by using saturated solution of ammonium chloride (15 mL). The water layer was washed with DCM (3 x15 mL). Combined organic extracts were washed with brine (20 mL) and dried over $MgSO_4$. The solvents were evaporated under reduced pressure and the resulting crude product was purified by column chromatography (eluent DCM : Acetone 20:1).

The following abbreviations are used to simplify the analysis of the spectra:
trans = r-2-trans-3-trans-6
enol = trans-enol
cis = r-2-cis-3-trans-6

5-Diethoxyphosphoryl-2-ethyltetrahydropyran-4-one (**14a**) (188 mg, 71%) Colourless oil.^{31}P NMR (284 MHz, Chloroform-*d*) δ 19.95 (cis), 20.51 (trans), 23.47 (enol). ^{1}H NMR (700 MHz, Chloroform-*d*) δ 0.91 – 0.99 (m, 3H trans + 3H cis + 3H enol), 1.28 – 1.36 (m, 6H trans + 6H cis + 6H enol), 1.49 – 1.72 (m, 2H trans + 2H cis + 2H enol), 2.17 (ddt, *J* = 6.4, 2.2, 1.2 Hz, 2H, enol), 2.25 (ddt, *J* = 14.5, 10.7, 0.9 Hz, 1H, cis), 2.40 (ddt, *J* = 14.7, 2.7, 1.4 Hz, 1H, trans), 2.51 (ddd, *J* = 14.6, 4.4, 3.0 Hz, 1H, cis), 2.67 (dd, *J* = 14.7, 11.4 Hz, 1H, trans), 2.91 (ddd, *J* = 23.9, 4.3, 1.3 Hz, 1H, trans), 3.14 (dddd, *J* = 20.8, 11.0, 6.6, 1.0 Hz, 1H, cis), 3.44 – 3.48 (m, 1H, enol), 3.50 (dddd, *J* = 11.7, 6.9, 5.1, 2.6 Hz, 1H, trans), 3.60 (dddd, *J* = 10.3, 7.0, 5.2, 2.9 Hz, 1H, cis), 3.83 (ddd, *J* = 34.7, 12.0, 4.3 Hz, 1H, trans), 3.81 – 3.88 (m, 1H, cis), 3.98 – 4.25 (m, 4H trans + 4H cis + 4H enol), 4.45 (ddd, *J* = 11.5, 6.6, 3.0 Hz, 1H, cis), 4.65 (ddd, *J* = 15.5, 12.0, 1.0 Hz, 1H, trans), 10.66 (d, *J* = 1.1 Hz, 1H, enol). ^{13}C NMR (176 MHz, Chloroform-*d*) δ 9.51 (cis), 9.55 (trans), 9.71 (enol), 16.32 (d, *J* = 6.6 Hz, enol), 16.36 (d, *J* = 6.4 Hz, enol), 16.41 (d, *J* = 6.2 Hz, trans), 16.51 (d, *J* = 5.9 Hz, 2×C, trans + cis), 16.56 (d, *J* = 6.2 Hz, cis), 28.76 (enol), 29.13 (cis), 29.28 (trans), 34.09 (d, *J* = 12.1 Hz, enol), 46.77 (trans), 47.89 (d, *J* = 4.5 Hz, cis), 51.00 (d, *J* = 141.4 Hz, cis), 51.86 (d, *J* = 127.2 Hz, trans), 62.00 (d, *J* = 5.0 Hz, enol), 62.18 (d, *J* = 4.6 Hz, enol), 62.31 (d, *J* = 6.5 Hz, cis), 62.66 (d, *J* = 6.3 Hz, cis), 62.85 (d, *J* = 6.7 Hz, trans), 63.04 (d, *J* = 6.4 Hz, trans), 63.65 (d, *J* = 14.3 Hz, enol), 66.72 (d, *J* = 7.6 Hz, trans), 67.08 (cis), 75.82 (enol), 79.50 (cis), 79.75 (trans), 88.69 (d, *J* = 179.9 Hz, enol), 167.24 (d, *J* = 3.7 Hz, enol), 202.02 (d, *J* = 3.4 Hz, trans), 202.07 (d, *J* = 7.4 Hz, cis). ESI-MS $[M+Na]^+$ = 287.1. Anal. Calcd for $C_{11}H_{21}O_5P$: C, 50.00; H, 8.01. Found: C, 50.21; H, 7.99.dd

Due to overlap of signals, the signals of cis and enol forms in the ^{1}H NMR and ^{13}C NMR spectra have not been assigned.

5-Diethoxyphosphoryl-2-isopropyltetrahydropyran-4-one (**14b**) (212 mg, 76%) Colourless oil.^{31}P NMR (284 MHz, Chloroform-*d*) δ 19.92 (cis), 20.61 (trans), 23.47 (enol). ^{1}H NMR (700 MHz, Chloroform-*d*) δ 0.87 (d, *J* = 6.8 Hz, 3H, cis), 0.88 (d, *J* = 6.6 Hz, 3H, enol), 0.89 (d, *J* = 6.9 Hz, 3H, trans), 0.91 (d, *J* = 6.8 Hz, 3H, cis), 0.93 (d, *J* = 6.8 Hz, 3H, trans), 0.93 (d, *J* = 6.7 Hz, 3H, enol), 1.23 – 1.34 (m, 6H trans + 6H cis + 6H enol), 1.66 – 1.81 (m, 1H trans + 1H cis + 1H enol), 2.11 (ddd, *J* = 17.4, 3.6, 2.1 Hz, 1H, enol), 2.18 (dddd, *J* = 16.5, 10.8, 2.9, 1.5 Hz, 1H, enol), 2.25 (ddd, *J* = 14.4, 11.0, 0.9 Hz, 1H, cis), 2.35 (ddd, *J* = 14.5, 2.5, 1.3 Hz, 1H, trans), 2.45 (ddd, *J* = 14.4, 4.4, 2.9 Hz, 1H, cis), 2.66 (dd, *J* = 14.6, 11.6 Hz, 1H, trans), 2.87 (dddd, *J* = 24.0, 4.3, 1.6, 1.0 Hz, 1H, trans), 3.10 (dddd, *J* = 20.7, 11.2, 6.8, 0.9 Hz, 1H, cis), 3.18 (ddd, *J* = 10.6, 6.9, 3.6 Hz, 1H, enol), 3.27 (ddd, *J* = 11.6, 5.9, 2.5 Hz, 1H, trans), 3.36 (ddd, *J* = 11.0,

6.1, 2.9 Hz, 1H, cis), 3.77 (ddd, *J* = 35.0, 12.0, 4.3 Hz, 1H, trans), 3.74 – 3.80 (m, 1H, cis), 3.95 – 4.21 (m, 4H trans + 4H cis + 4H enol), 4.42 (ddd, *J* = 11.5, 6.8, 2.3 Hz, 1H, cis), 4.62 (ddd, *J* = 15.8, 11.9, 0.9 Hz, 1H, trans), 10.62 (d, *J* = 1.1 Hz, 1H, enol). ^{13}C NMR (176 MHz, Chloroform-*d*) δ 16.24 (d, *J* = 6.8 Hz, enol), 16.27 (d, *J* = 5.7 Hz, enol), 16.33 (d, *J* = 5.9 Hz, trans), 16.43 (d, *J* = 6.1 Hz, 2×C, trans + cis), 16.45 (d, *J* = 6.0 Hz, cis), 17.83 (cis), 17.85 (trans), 17.87 (enol), 17.97 (cis), 18.00 (trans), 18.48 (enol), 31.77 (d, *J* = 12.1 Hz, enol), 32.93 (enol), 33.11 (cis), 33.16 (trans), 44.22 (trans), 45.39 (d, *J* = 4.6 Hz, cis), 50.92 (d, *J* = 141.4 Hz, cis), 51.76 (d, *J* = 127.1 Hz, trans), 61.89 (d, *J* = 5.0 Hz, enol), 62.06 (d, *J* = 4.6 Hz, enol), 62.20 (d, *J* = 6.5 Hz, cis), 62.51 (d, *J* = 6.3 Hz, cis), 62.79 (d, *J* = 6.7 Hz, trans), 62.85 (d, *J* = 6.4 Hz, trans), 63.83 (d, *J* = 14.0 Hz, enol), 66.69 (d, *J* = 7.5 Hz, trans), 67.15 (cis), 79.52 (enol), 82.99 (cis), 83.22 (trans), 88.61 (d, *J* = 179.8 Hz, enol), 167.45 (enol), 202.35 (d, *J* = 3.7 Hz, trans), 202.42 (d, *J* = 7.1 Hz, cis). ESI-MS $[M+Na]^+$ = 301.2. Anal. Calcd for $C_{12}H_{23}O_5P$: C, 51.79; H, 8.33. Found: C, 51.59; H, 8.35.

Due to overlap of signals, the signals of cis and enol forms in the ^{1}H NMR and ^{13}C NMR spectra have not been assigned.

5-Diethoxyphosphoryl-2-phenyltetrahydropyran-4-one (**14c**) (231 mg, 74%) Colourless oil.^{31}P NMR (284 MHz, Chloroform-*d*) δ 19.74 (cis), 20.27 (trans), 23.14 (enol). ^{1}H NMR (700 MHz, Chloroform-*d*) δ 1.28 – 1.38 (m, 6H trans + 6H cis + 6H enol), 2.44 (ddd, *J* = 17.5, 3.7, 1.9 Hz, 1H, enol), 2.51 – 2.58 (m, 1H, enol), 2.59 – 2.65 (m, 1H trans + 1H cis), 2.76 (ddd, *J* = 14.7, 4.4, 3.3 Hz, 1H, cis), 3.00 (ddd, *J* = 23.9, 4.3, 1.2 Hz, 1H, trans), 3.06 (dd, *J* = 14.9, 11.5 Hz, 1H, trans), 3.26 (dddd, *J* = 20.9, 10.8, 6.6, 1.0 Hz, 1H, cis), 4.00 (ddd, *J* = 34.7, 12.1, 4.2 Hz, 1H, trans), 3.98 – 4.05 (m, 1H, cis), 4.05 – 4.28 (m, 4H trans + 4H cis), 4.32 (ddd, *J* = 13.8, 5.6, 1.5 Hz, 1H, enol), 4.55 (ddd, *J* = 11.6, 6.6, 3.9 Hz, 1H, cis), 4.62 (dd, *J* = 10.5, 3.8 Hz, 1H, enol), 4.62 (dd, *J* = 11.6, 2.9 Hz, 1H, trans), 4.74 (dd, *J* = 10.8, 3.4 Hz, 1H, enol), 4.77 (ddd, *J* = 15.5, 12.2, 1.0 Hz, 1H, trans), 7.27 – 7.40 (m, 5H trans + 5H cis + 5H enol), 10.74 (d, *J* = 1.0 Hz, 1H, enol). ^{13}C NMR (176 MHz, Chloroform-*d*) δ 16.25 (d, *J* = 6.5 Hz, enol), 16.31 (d, *J* = 6.1 Hz, enol), 16.37 (d, *J* = 6.3 Hz, trans), 16.46 (d, *J* = 6.0 Hz, cis), 16.47 (d, *J* = 6.2 Hz, trans), 16.49 (d, *J* = 6.1 Hz, cis), 35.65 (d, *J* = 12.2 Hz, enol), 48.73 (trans), 49.54 (d, *J* = 4.5 Hz, cis), 50.85 (d, *J* = 141.5 Hz, cis), 51.67 (d, *J* = 127.6 Hz, trans), 62.04 (d, *J* = 5.0 Hz, enol), 62.18 (d, *J* = 4.5 Hz, enol), 62.31 (d, *J* = 6.6 Hz, cis), 62.70 (d, *J* = 6.4 Hz, cis), 62.97 (d, *J* = 6.3 Hz, trans), 62.98 (d, *J* = 6.5 Hz, trans), 63.85 (d, *J* = 14.1 Hz, enol), 66.87 (d, *J* = 7.6 Hz, trans), 67.03 (cis), 75.99 (enol), 79.64 (cis), 80.14 (trans), 88.66 (d, *J* = 179.7 Hz, enol), 125.74 (cis, 2×C), 125.80 (trans, 2×C), 125.86 (enol, 2×C), 128.03 (enol), 128.30 (trans), 128.36 (cis), 128.61 (enol, 2×C), 128.71 (trans, 2×C), 128.76 (cis, 2×C), 140.03 (cis), 140.40 (trans), 140.90 (enol), 166.78 (d, *J* = 3.9 Hz, enol), 201.06 (d, *J* = 3.5 Hz, trans), 201.20 (d, *J* = 7.0 Hz, cis). ESI-MS $[M+Na]^+$ = 335.1. Anal. Calcd for $C_{15}H_{21}O_5P$: C, 57.69; H, 6.78. Found: C, 57.62; H, 6.78.

Due to overlap of signals, the signals of cis and enol forms in the ^{1}H NMR and ^{13}C NMR spectra have not been assigned.

5-Diethoxyphosphoryl-2-(4-methoxyphenyl)tetrahydropyran-4-one (**14d**) (213 mg, 62%) Colourless oil.^{31}P NMR (101 MHz, Chloroform-*d*) δ 20.19 (cis), 20.65 (trans), 23.55 (enol). ^{1}H NMR (700 MHz, Chloroform-*d*) δ 1.25 – 1.35 (m, 6H trans + 6H cis + 6H enol), 2.37 (ddd, *J* = 17.5, 3.7, 1.9 Hz, 1H, enol), 2.55 (dd, *J* = 14.9, 3.3 Hz, 1H, trans), 2.59 (dd, *J* = 14.7, 10.6 Hz, 1H, cis), 2.70 (dd, *J* = 14.7, 3.8 Hz, 1H, cis), 2.96 (dd, *J* = 23.8, 4.1 Hz, 1H, trans), 3.04 (dd, *J* = 14.9, 11.5 Hz, 1H, trans), 3.22 (ddd, *J* = 20.8, 10.6, 6.5 Hz, 1H, cis), 3.70 – 3.79 (m, 3H trans + 3H cis + 3H enol), 3.95 (ddd, *J* = 34.8, 12.3, 4.2 Hz, 1H, trans), 3.94 – 4.00 (m, 1H,

cis), 4.12 – 4.23 (m, 4H trans + 4H cis + 4H enol), 4.24 – 4.28 (m, 1H, enol), 4.48 (ddd, *J* = 11.2, 6.6, 4.3 Hz, 1H, cis), 4.54 (dd, *J* = 11.4, 2.9 Hz, 1H, trans), 4.70 (dd, *J* = 15.1, 11.8 Hz, 1H, trans), 6.82 – 6.87 (m, 2H trans + 2H cis + 2H enol), 7.20 – 7.22 (m, 2H, cis), 7.24 (d, *J* = 8.5 Hz, 2H, enol), 7.27 (d, *J* = 8.6 Hz, 2H, trans), 10.71 (s, 1H, enol). ^{13}C NMR (176 MHz, Chloroform-*d*) δ 16.11 (d, *J* = 6.6 Hz, enol), 16.17 (d, *J* = 5.7 Hz, enol), 16.24 (d, *J* = 6.1 Hz, trans), 16.31 (d, *J* = 5.7 Hz, cis), 16.35 (d, *J* = 6.3 Hz, 2×C, trans + cis), 35.41 (d, *J* = 12.3 Hz, enol), 48.55 (trans), 49.26 (d, *J* = 4.5 Hz, cis), 50.70 (d, *J* = 141.0 Hz, cis), 51.53 (d, *J* = 127.3 Hz, trans), 55.18 (3×C, trans + cis + enol) 61.89 (d, *J* = 4.8 Hz, enol), 62.02 (d, *J* = 4.7 Hz, enol), 62.16 (d, *J* = 6.6 Hz, cis), 62.54 (d, *J* = 6.3 Hz, cis), 62.81 (d, *J* = 6.4 Hz, trans), 62.83 (d, *J* = 6.8 Hz, trans), 63.65 (d, *J* = 14.1 Hz, enol), 66.63 (d, *J* = 7.5 Hz, trans), 66.70 (cis), 75.50 (enol), 79.20 (cis), 79.71 (trans), 88.50 (d, *J* = 179.6 Hz, enol), 113.85 (enol, 2×C), 113.94 (trans, 2×C), 113.98 (cis, 2×C), 127.11 (enol, 2×C), 127.14 (trans, 2×C), 127.15 (cis, 2×C), 132.04 (cis), 132.47 (trans), 132.91 (enol), 159.29 (enol), 159.47 (trans), 159.50 (cis), 166.77 (d, *J* = 3.7 Hz, enol), 201.07 (d, *J* = 3.3 Hz, trans), 201.24 (d, *J* = 6.8 Hz, cis). ESI-MS $[M+Na]^+$ = 365.0. Anal. Calcd for $C_{16}H_{23}O_6P$: C, 56.14; H, 6.77. Found: C, 56.29; H, 6.80.

Due to overlap of signals, the signals of cis and enol forms in the ^{1}H NMR and ^{13}C NMR spectra have not been assigned.

1.6 NMR Spectra

Diethyl (4-hydroxy-2-oxohexyl)phosphonate (**9a**)

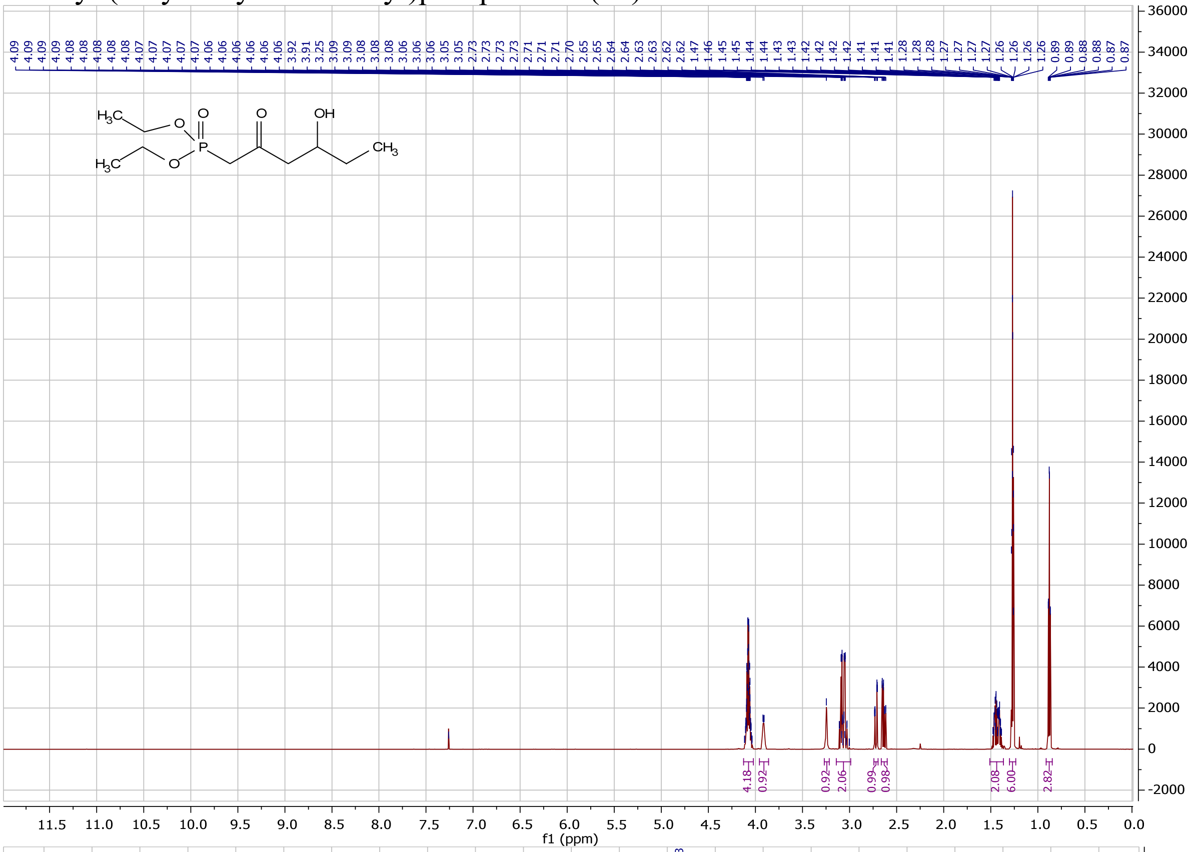

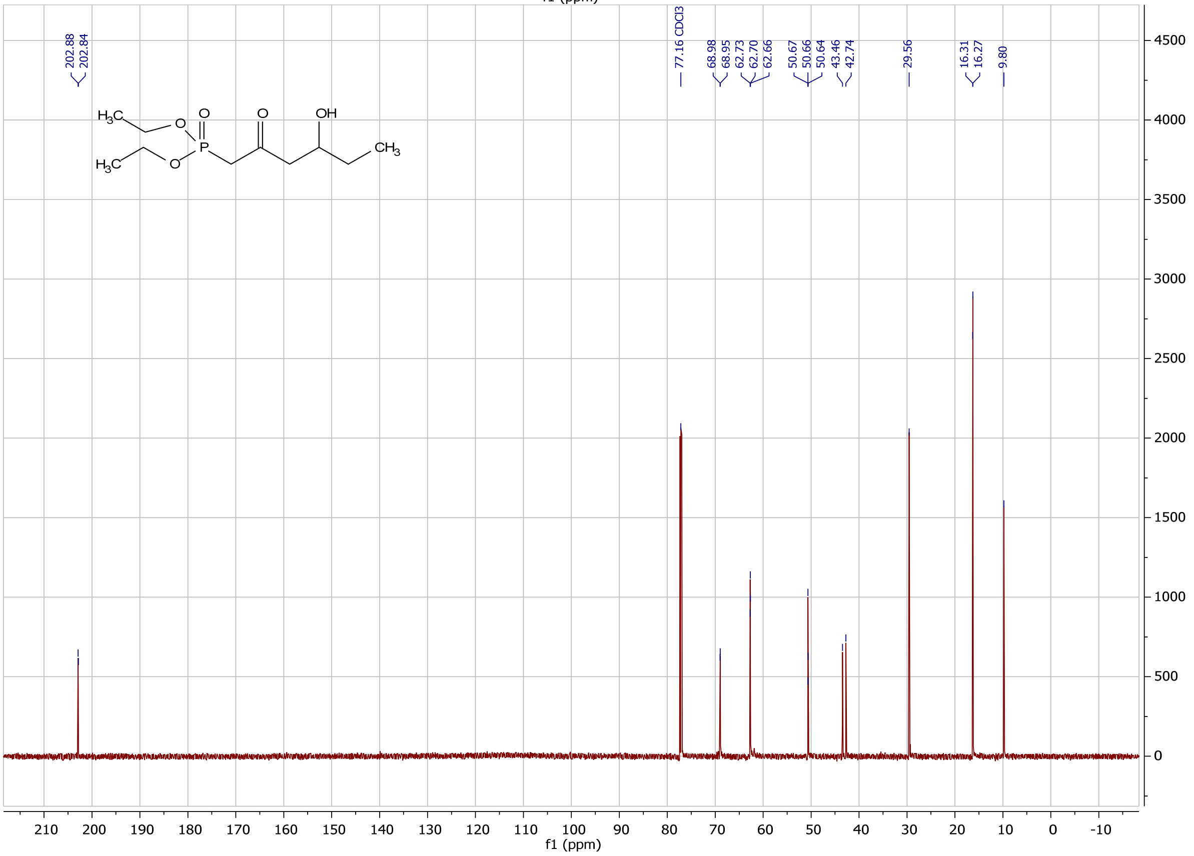

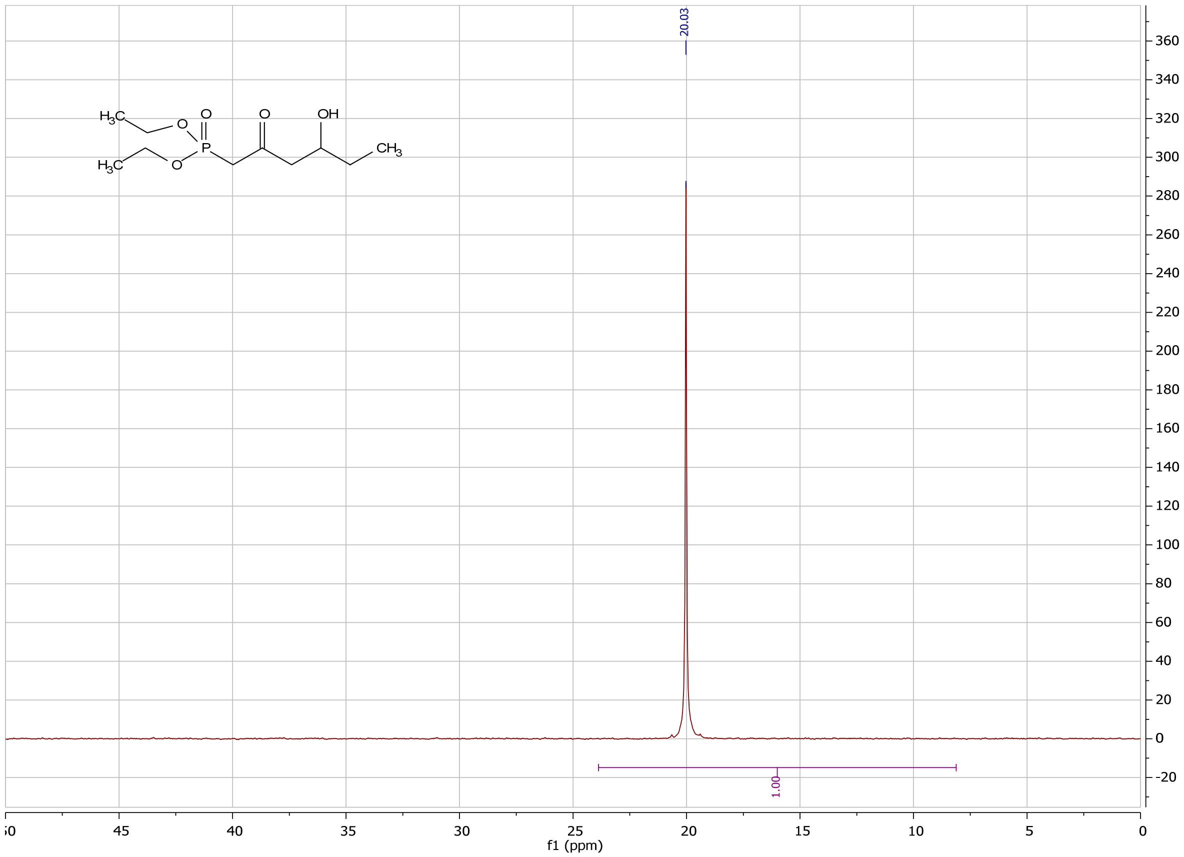

Diethyl (4-hydroxy-5-methyl-2-oxohexyl)phosphonate (**9b**)

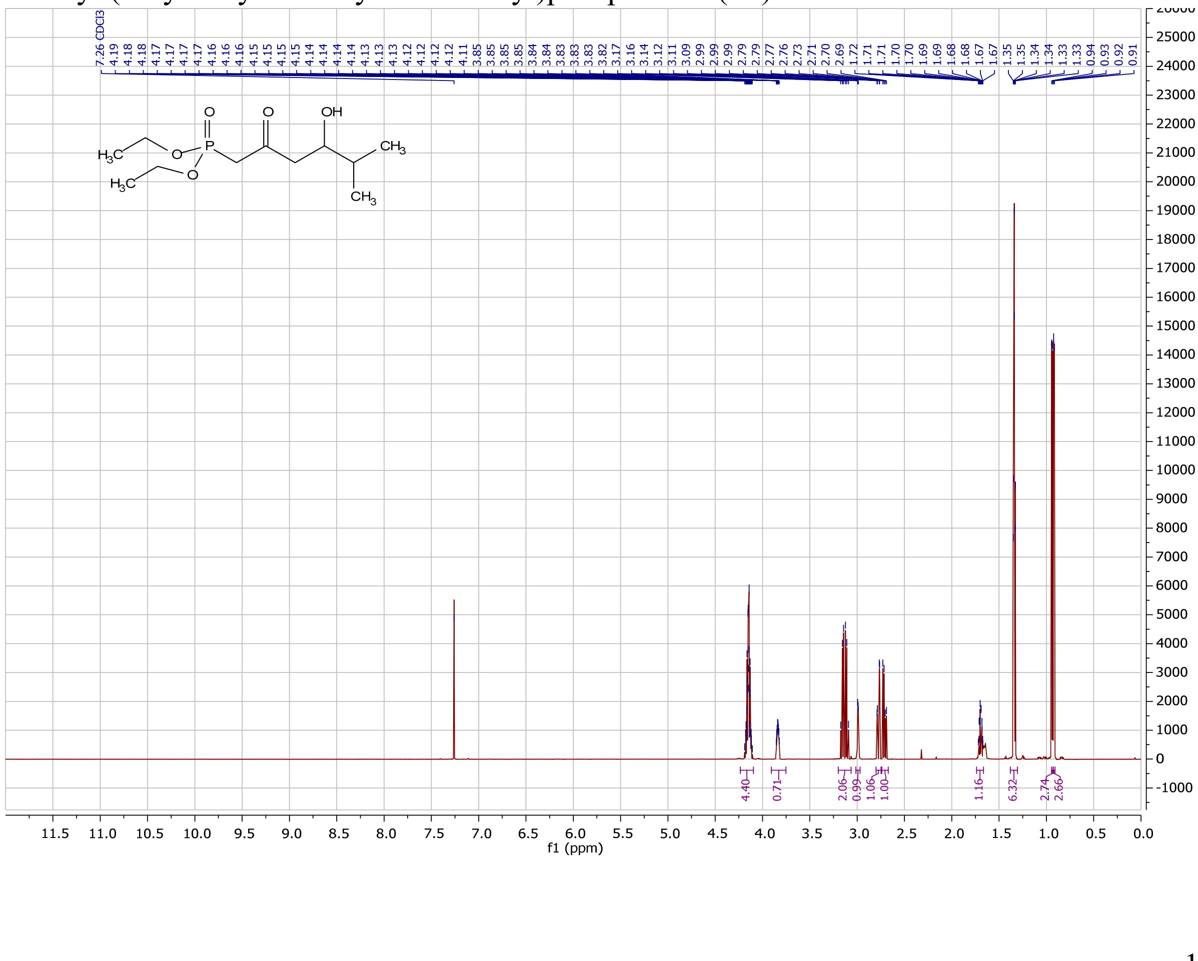

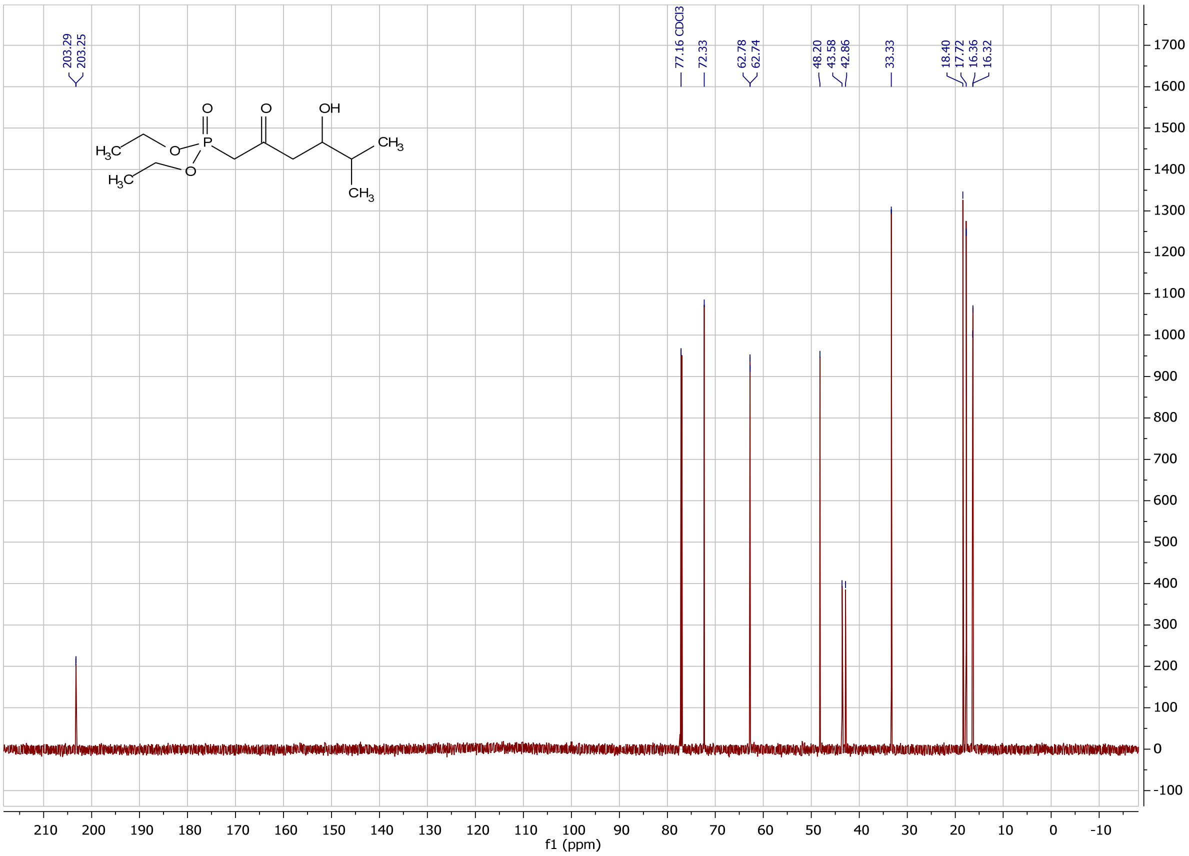
203.29
203.25
77.16 CDCl3
72.33
62.78
62.74
48.20
43.58
42.86
33.33
18.40
17.72
16.36
16.32
f1 (ppm)

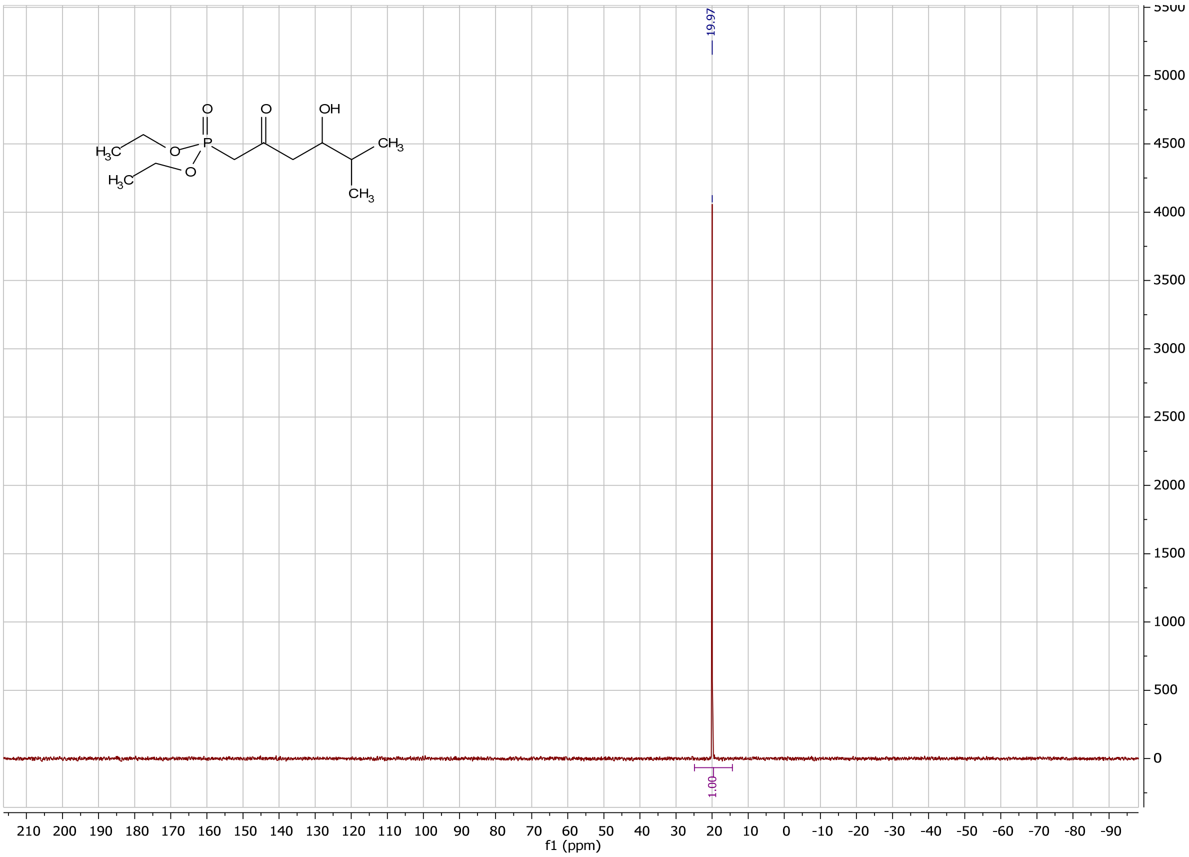
19.97
1.00
f1 (ppm)

Diethyl (4-hydroxy-2-oxo-4-phenylbutyl)phosphonate (**9c**)

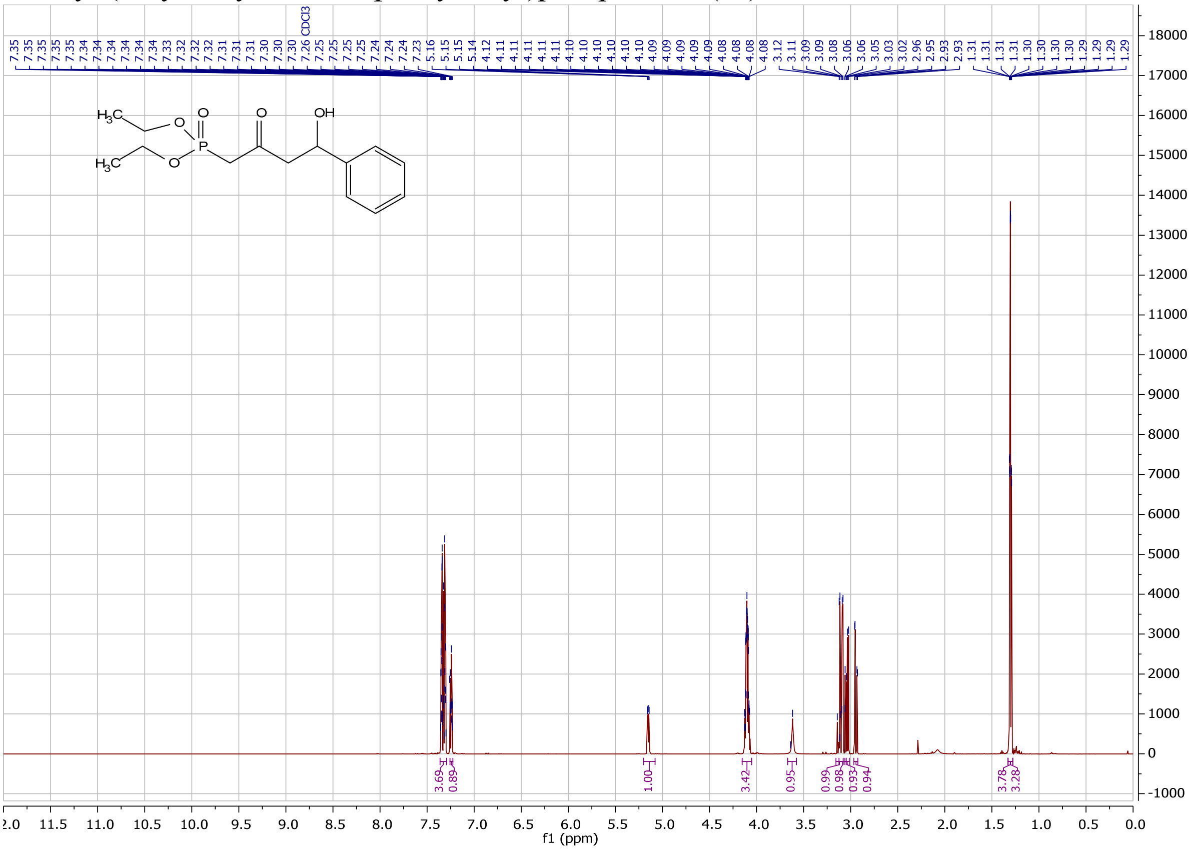

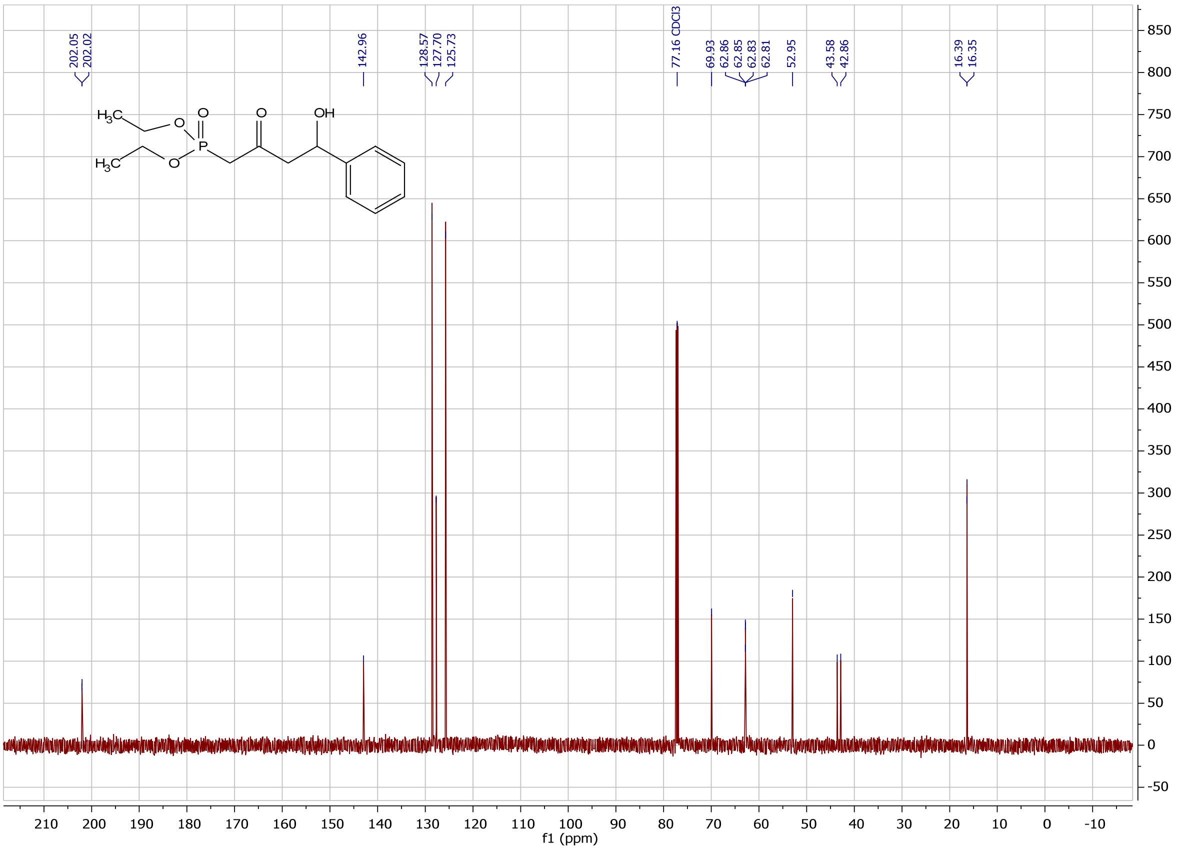

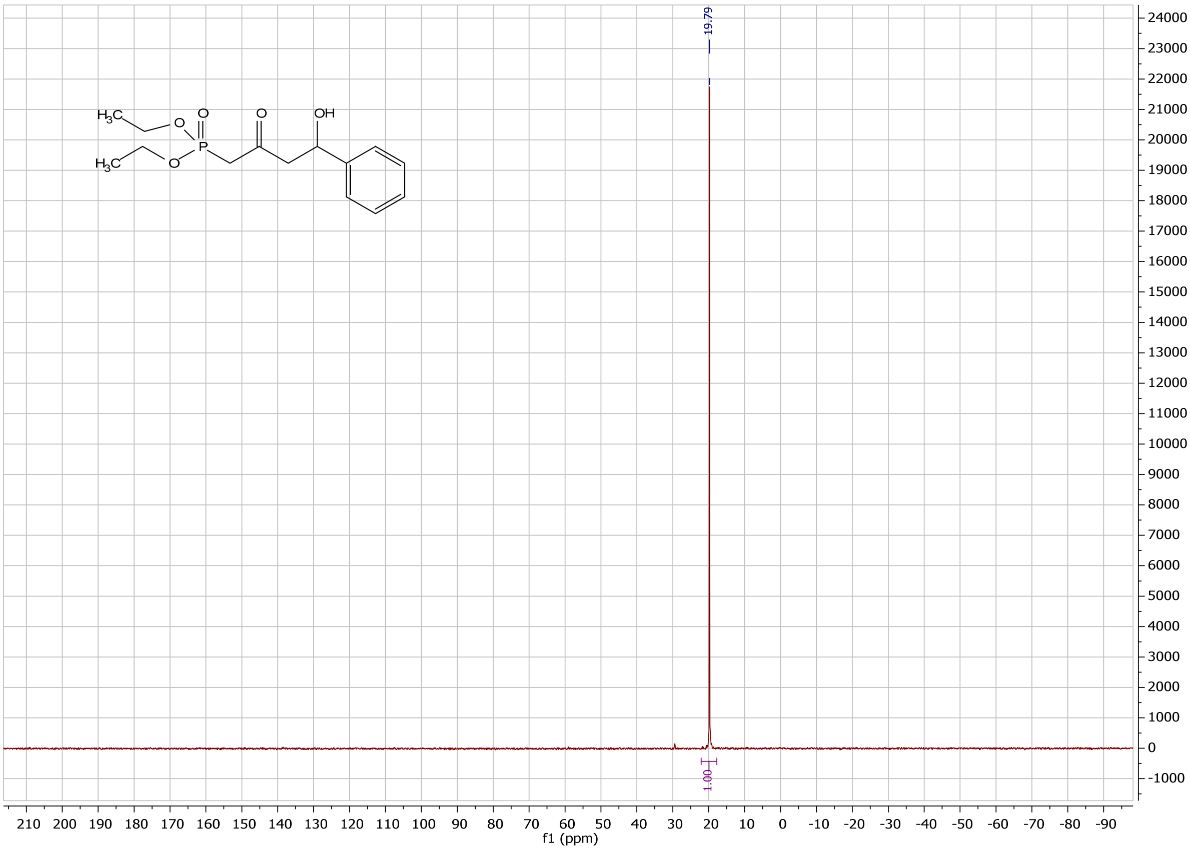

Diethyl (4-hydroxy-4-(4-methoxyphenyl)-2-oxobutyl)phosphonate (**9d**)

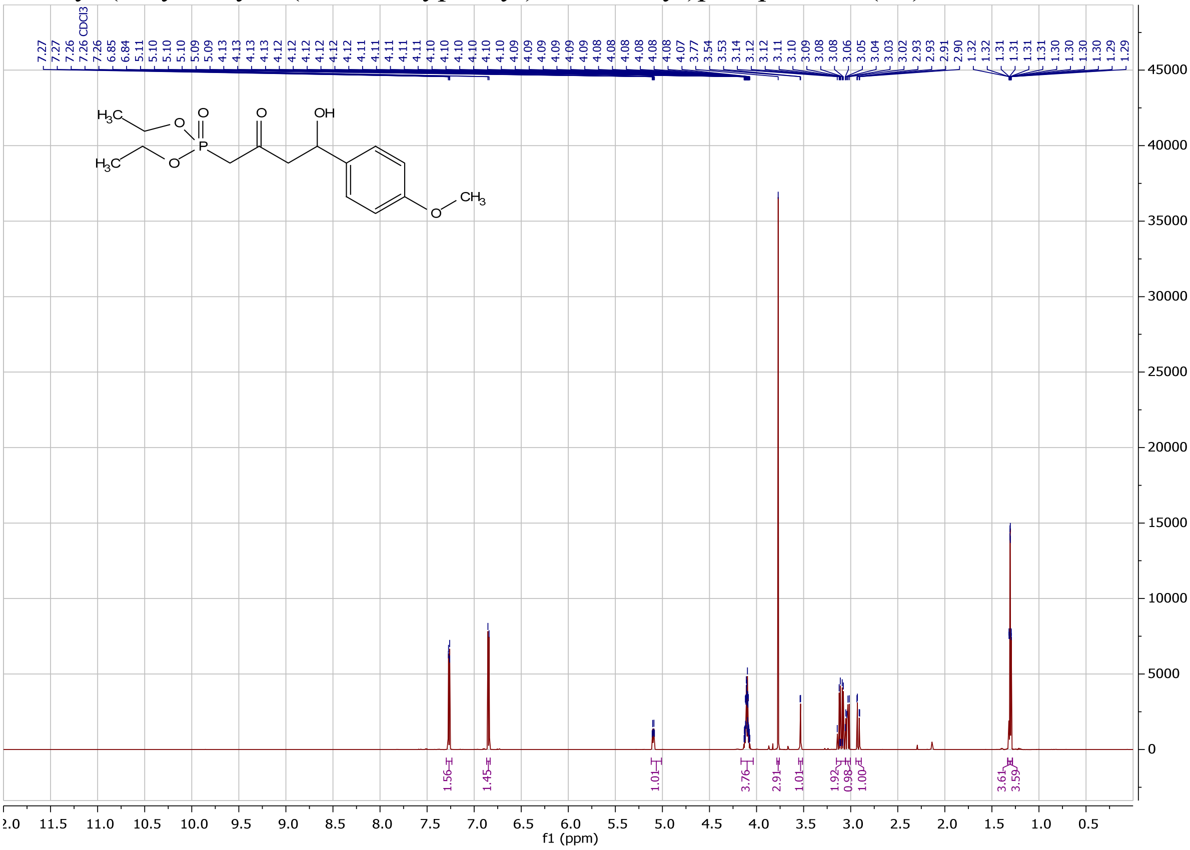

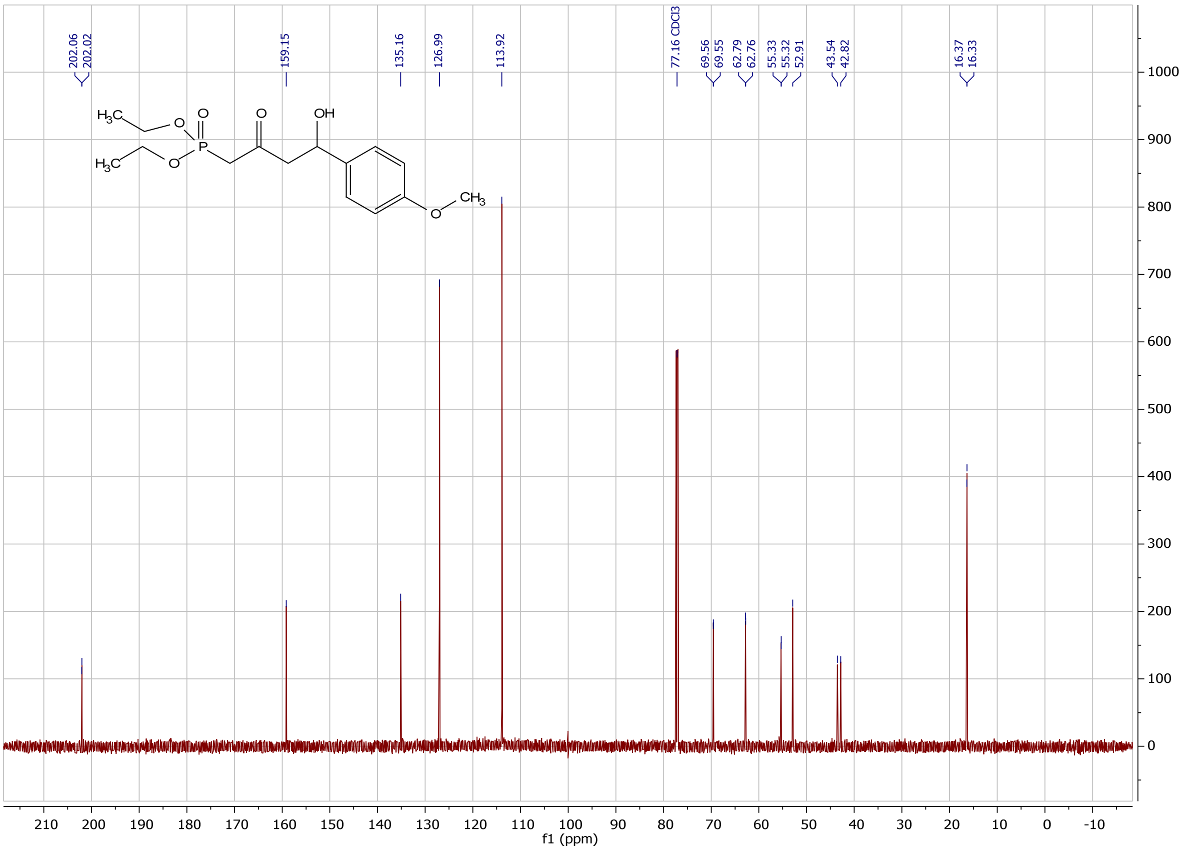
202.06
202.02
159.15
135.16
126.99
113.92
77.16 CDCl3
69.56
69.55
62.79
62.76
55.33
55.32
52.91
43.54
42.82
16.37
16.33
f1 (ppm)

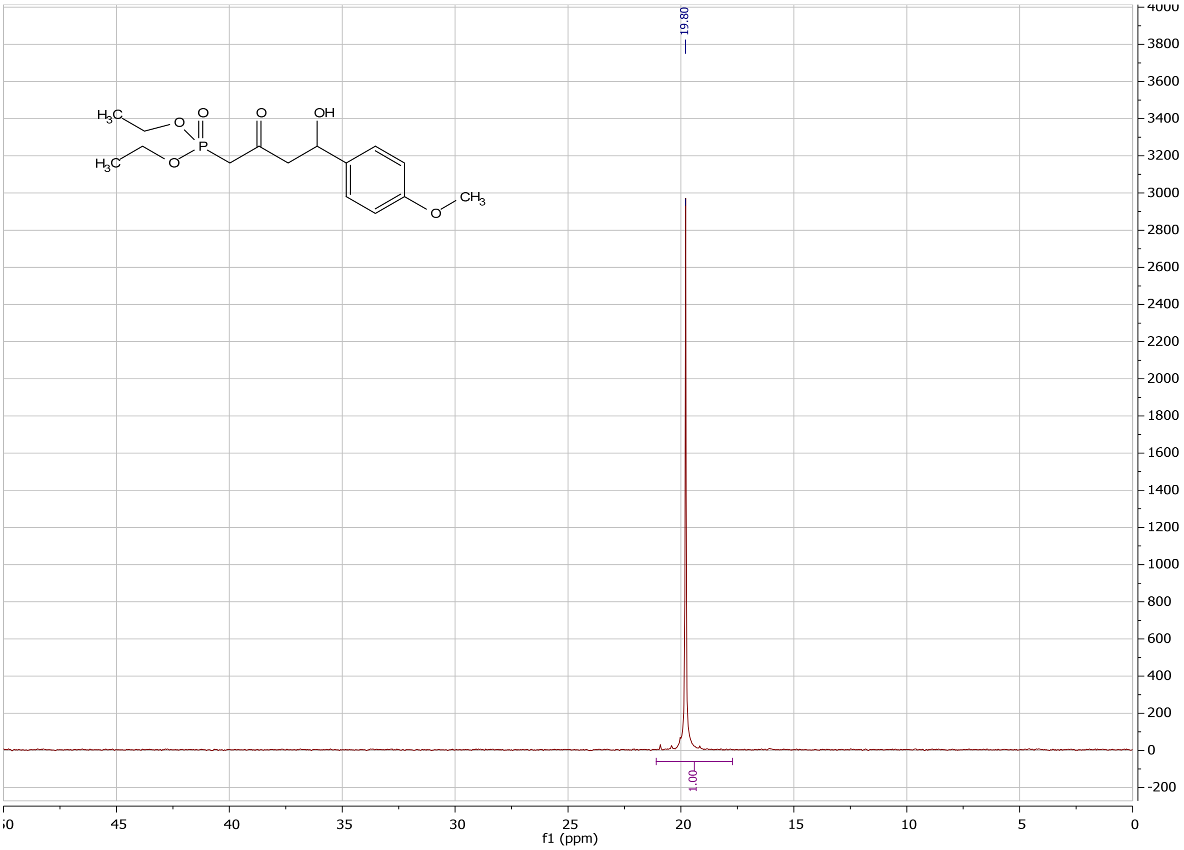
19.80
1.00
f1 (ppm)

Diethyl (4-hydroxy-2-oxo-4-phenylbutyl)phosphonate (**9e**)

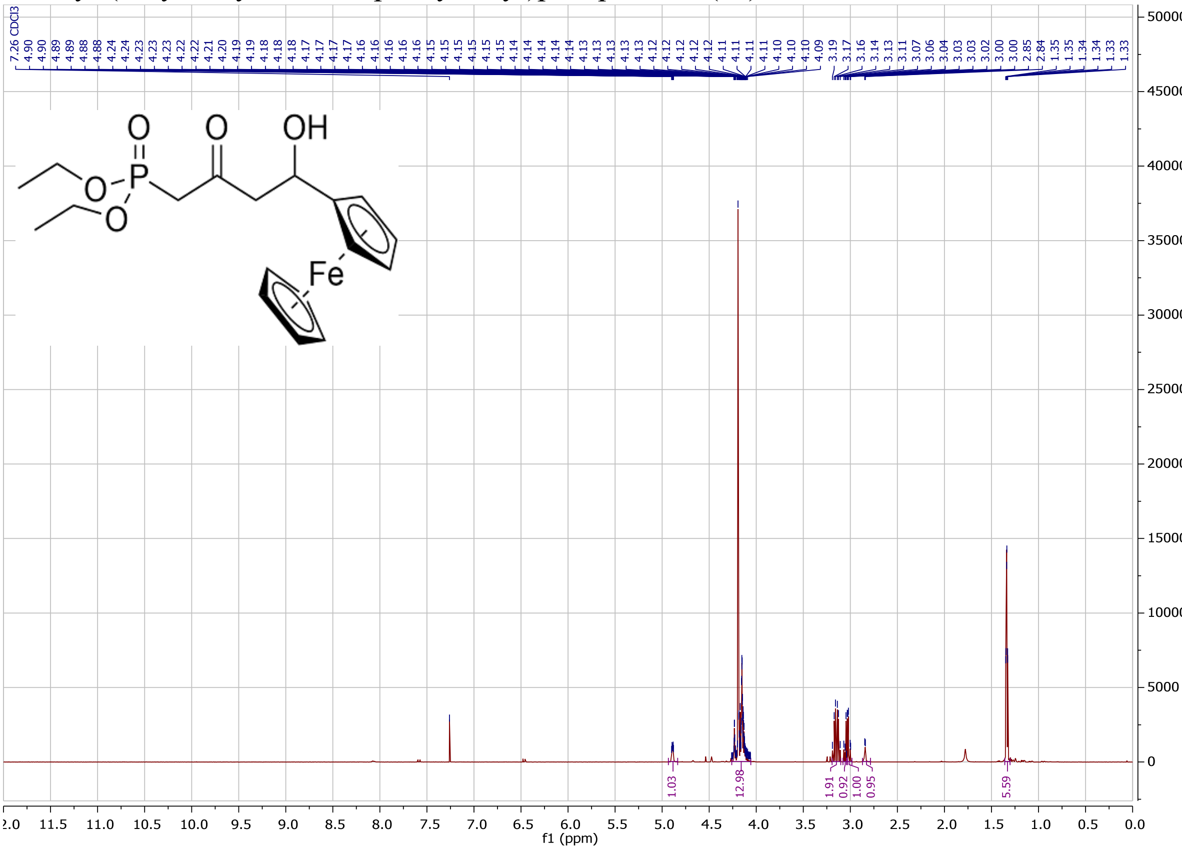

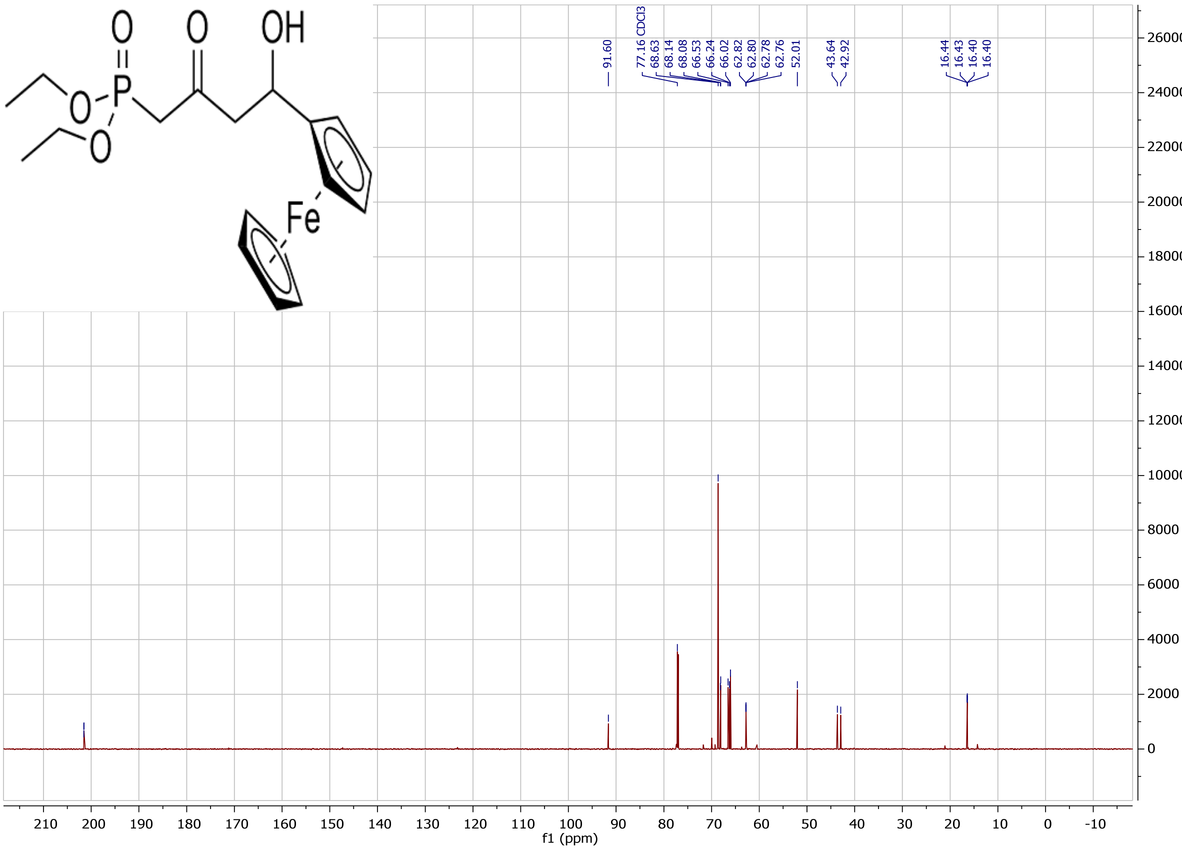

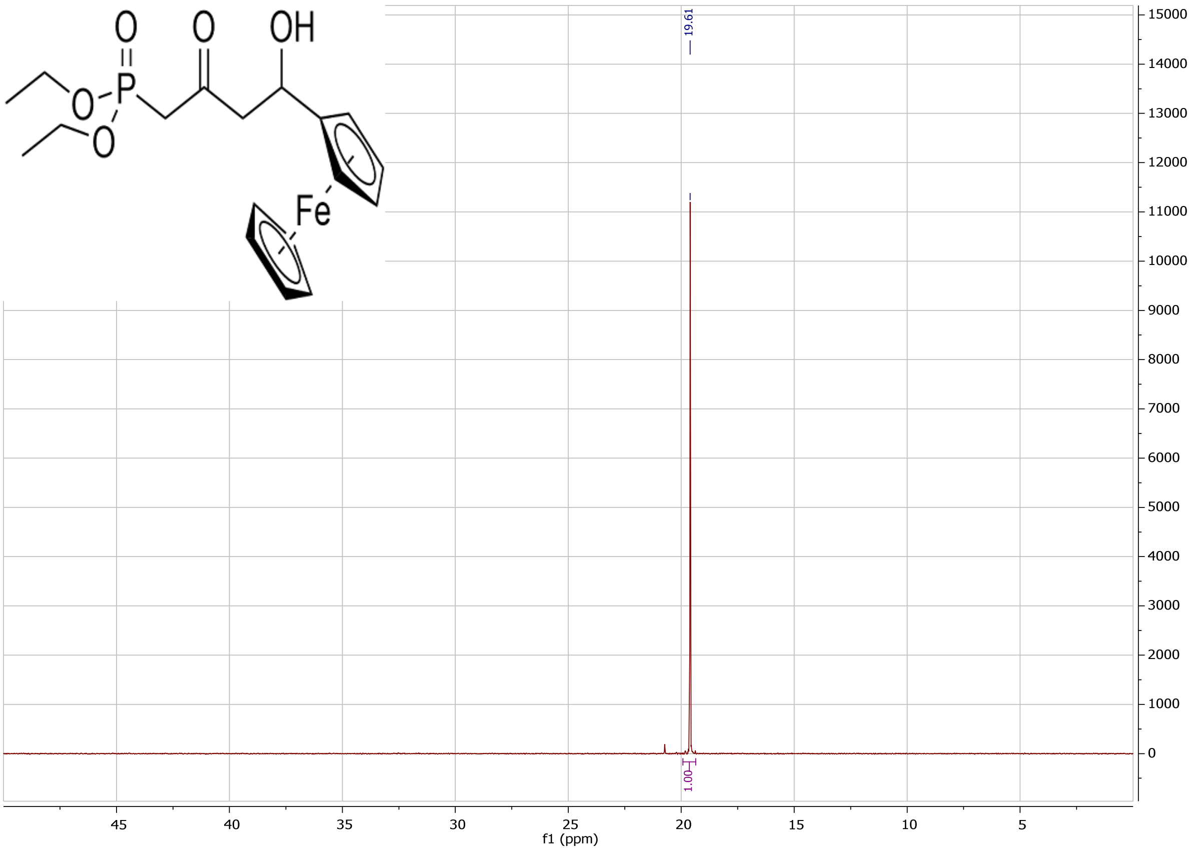

2-Butyl-3-diethoxyphosphoryl-6-ethyltetrahydropyran-4-one (**12a**)

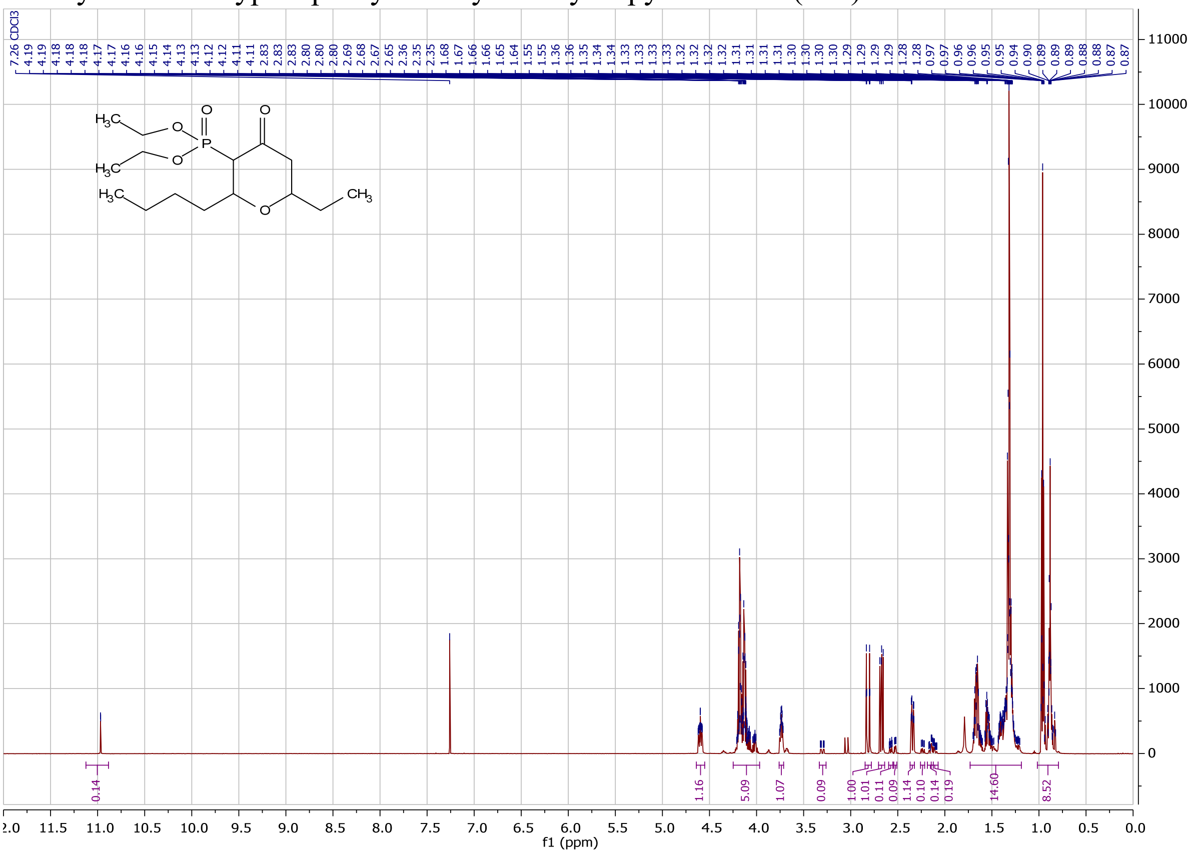

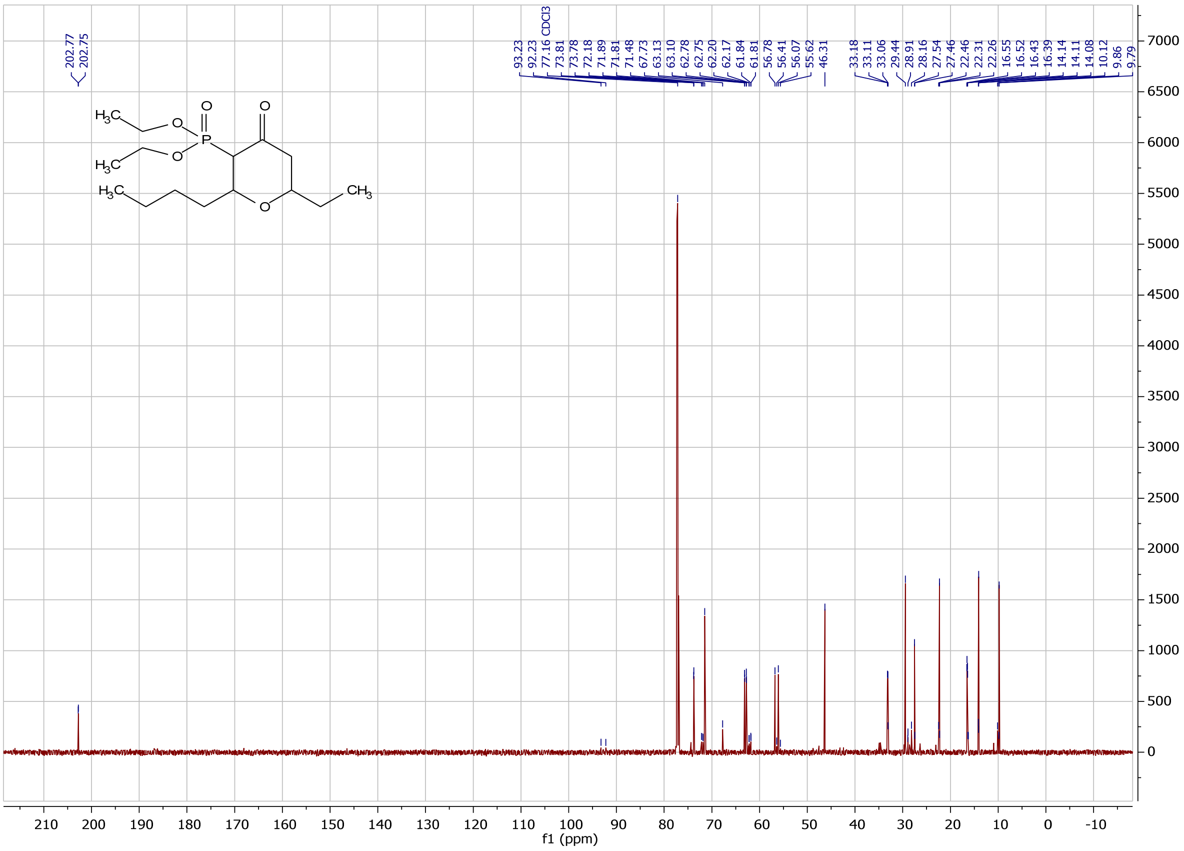

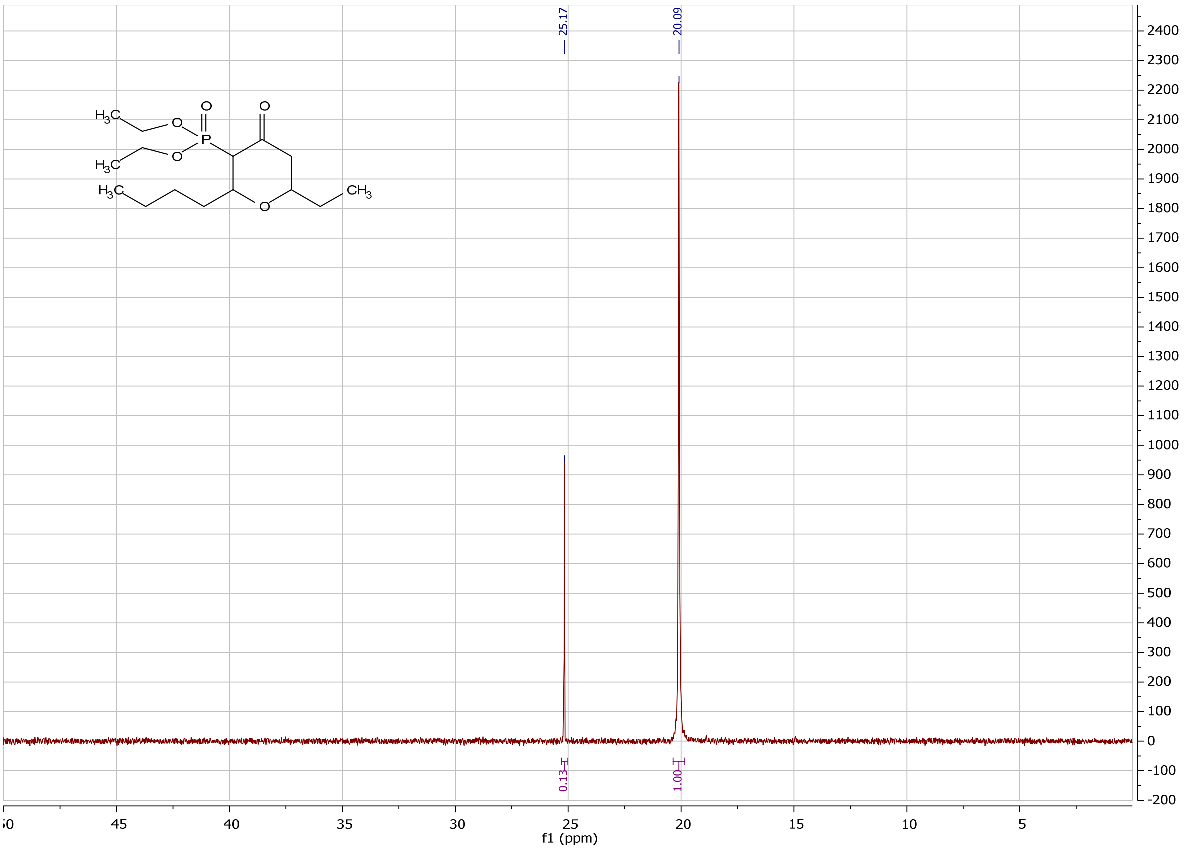

3-Diethoxyphosphoryl-6-ethyl-2-phenyltetrahydropyran-4-one (**12b**)

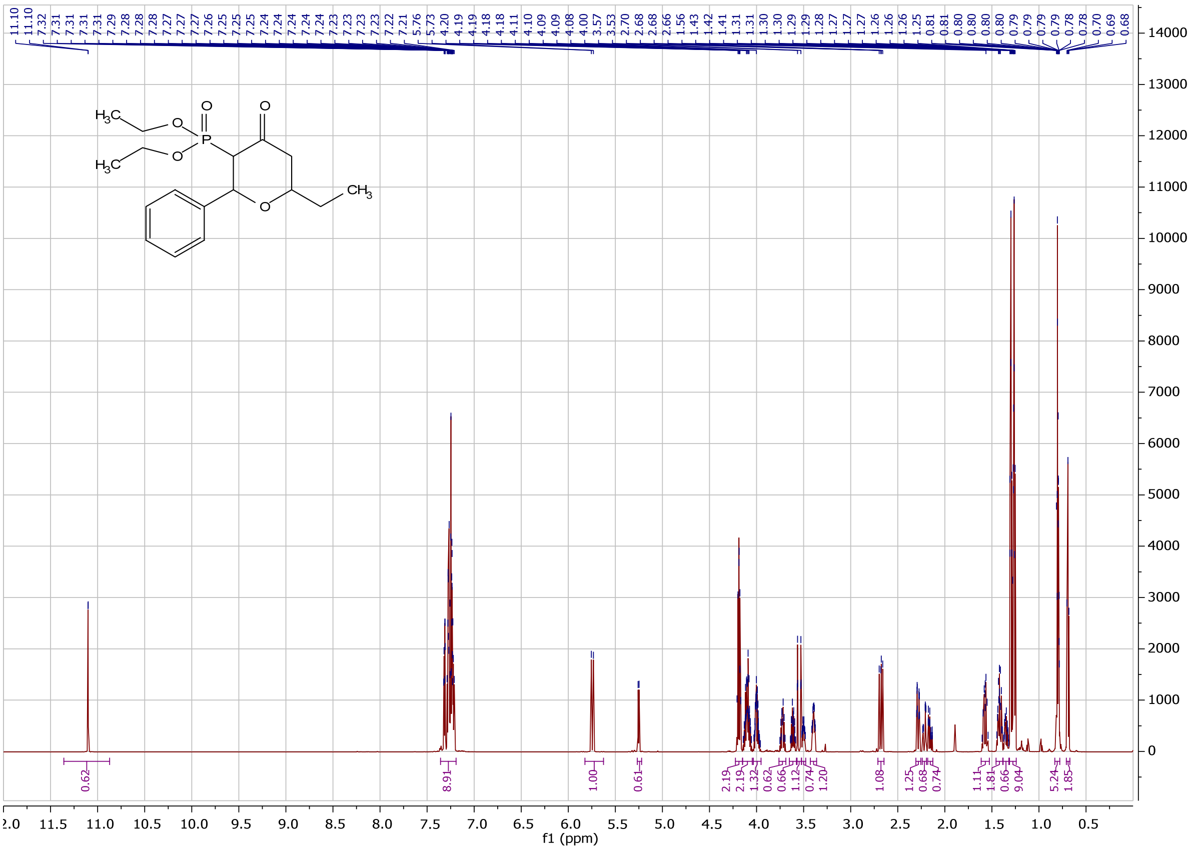

11.10 11.10 7.32 7.31 7.31 7.31 7.29 7.28 7.28 7.28 7.27 7.27 7.27 7.26 7.25 7.25 7.24 7.24 7.24 7.24 7.24 7.23 7.23 7.23 7.23 7.22 7.21 5.76 5.73 4.20 4.19 4.19 4.18 4.18 4.11 4.10 4.09 4.09 4.08 4.00 3.57 3.53 2.70 2.68 2.68 2.66 1.56 1.43 1.42 1.41 1.31 1.31 1.30 1.30 1.29 1.29 1.28 1.27 1.27 1.27 1.26 1.26 1.26 1.25 1.25 0.81 0.81 0.80 0.80 0.80 0.80 0.79 0.79 0.79 0.79 0.78 0.78 0.70 0.69 0.68
H3C
O
P
O
H3C
O
O
CH3
0.62 8.91 1.00 0.61 2.19 2.19 1.32 0.62 0.66 1.12 0.74 1.20 1.08 1.25 0.68 0.74 1.11 1.81 0.66 9.04 5.24 1.85
f1 (ppm)

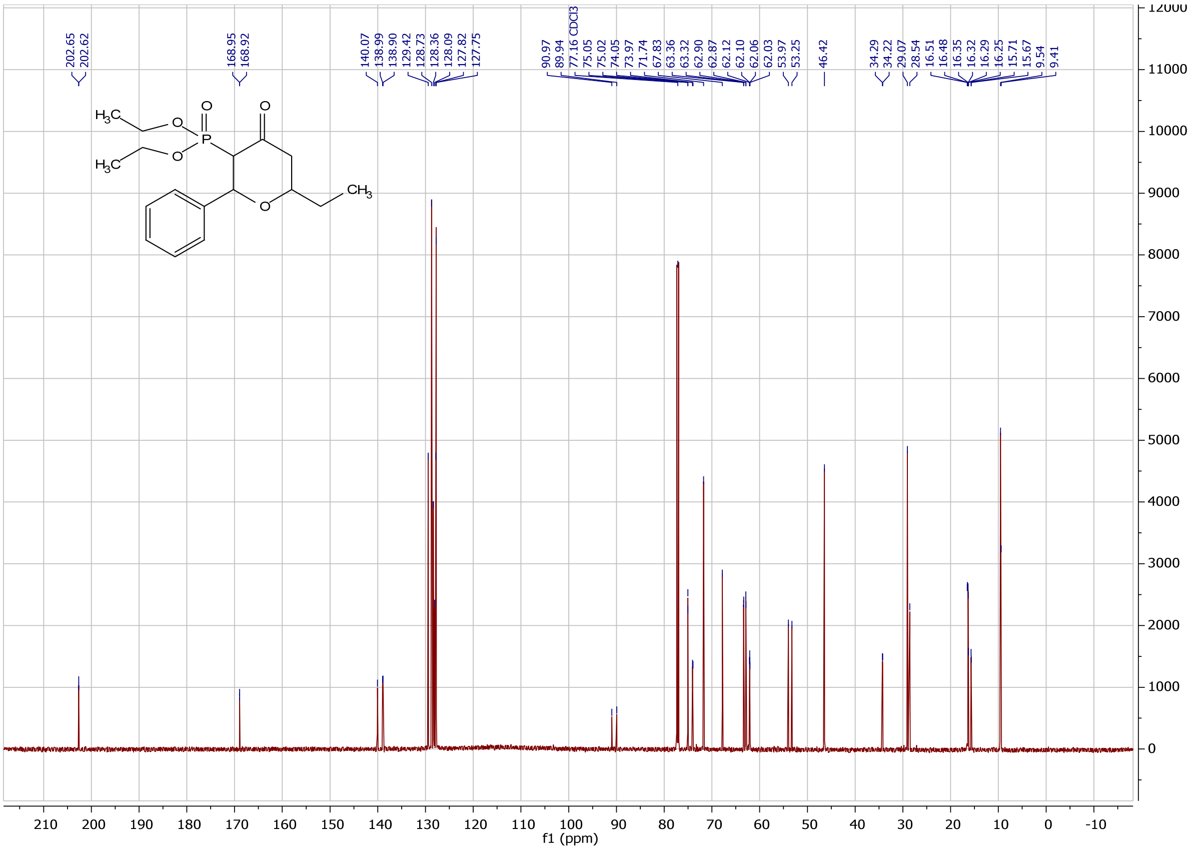

202.65 202.62 168.95 168.92 140.07 138.99 138.90 129.42 128.73 128.36 128.09 127.82 127.75 90.97 89.94 77.16 CDCl3 75.05 75.02 74.05 73.97 71.74 67.83 63.36 63.32 62.90 62.87 62.12 62.10 62.06 62.03 53.97 53.25 46.42 34.29 34.22 29.07 28.54 16.51 16.48 16.35 16.32 16.29 16.25 15.71 15.67 9.54 9.41
H3C
O
P
O
H3C
O
O
CH3
f1 (ppm)

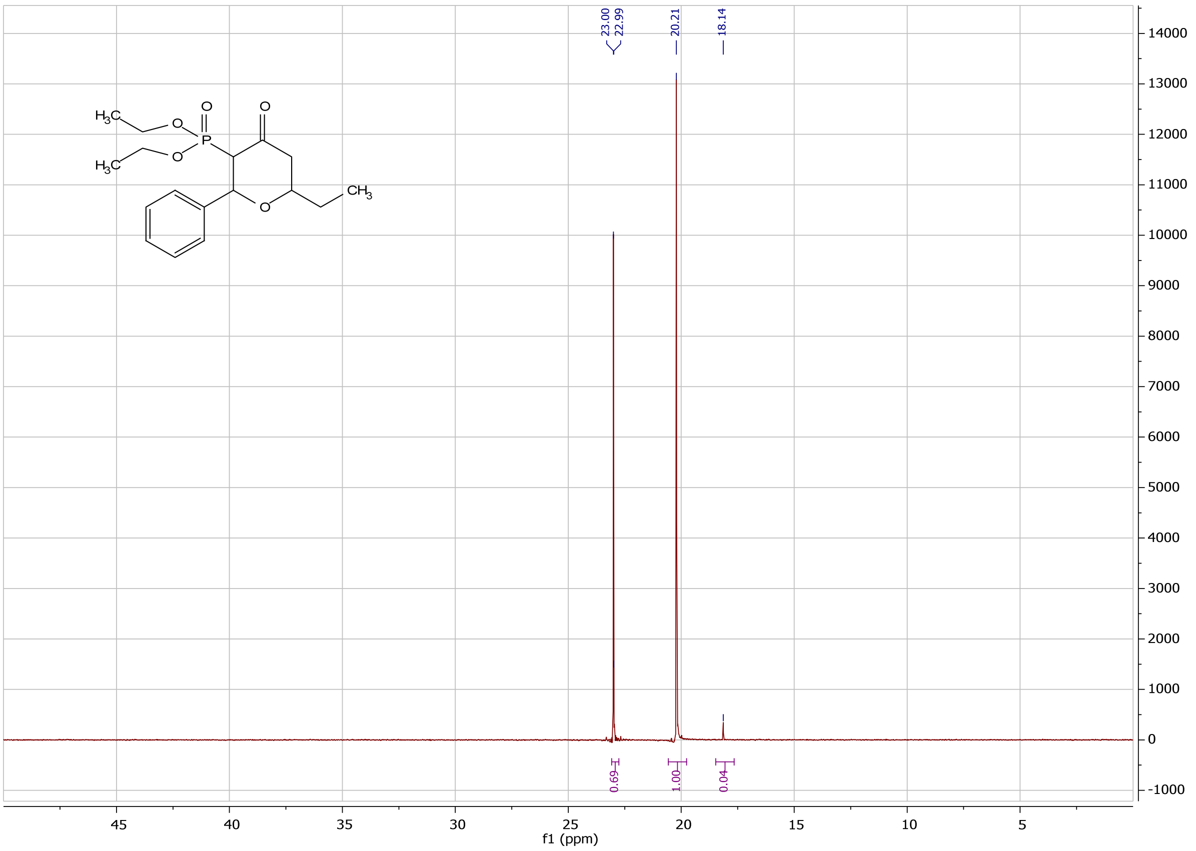

2-Butyl-3-diethoxyphosphoryl-6-isopropyltetrahydropyran-4-one (**12c**)

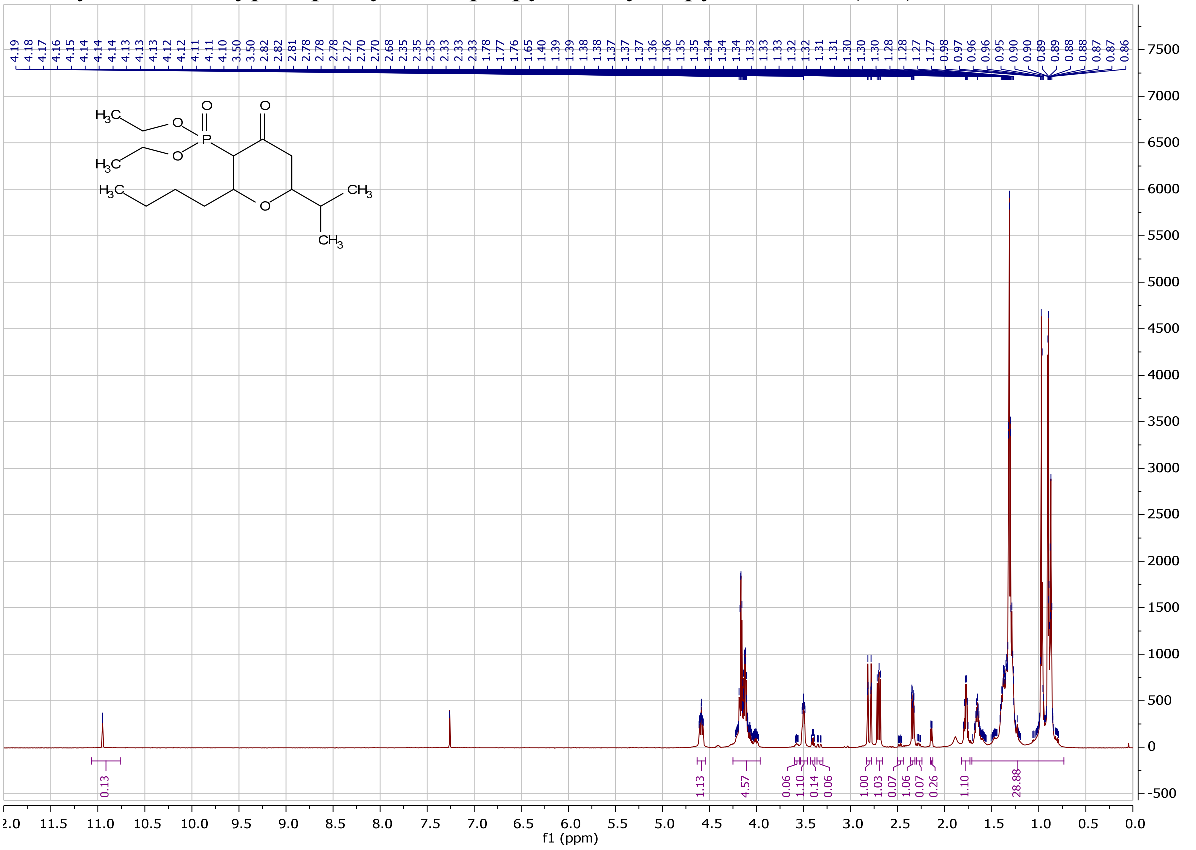

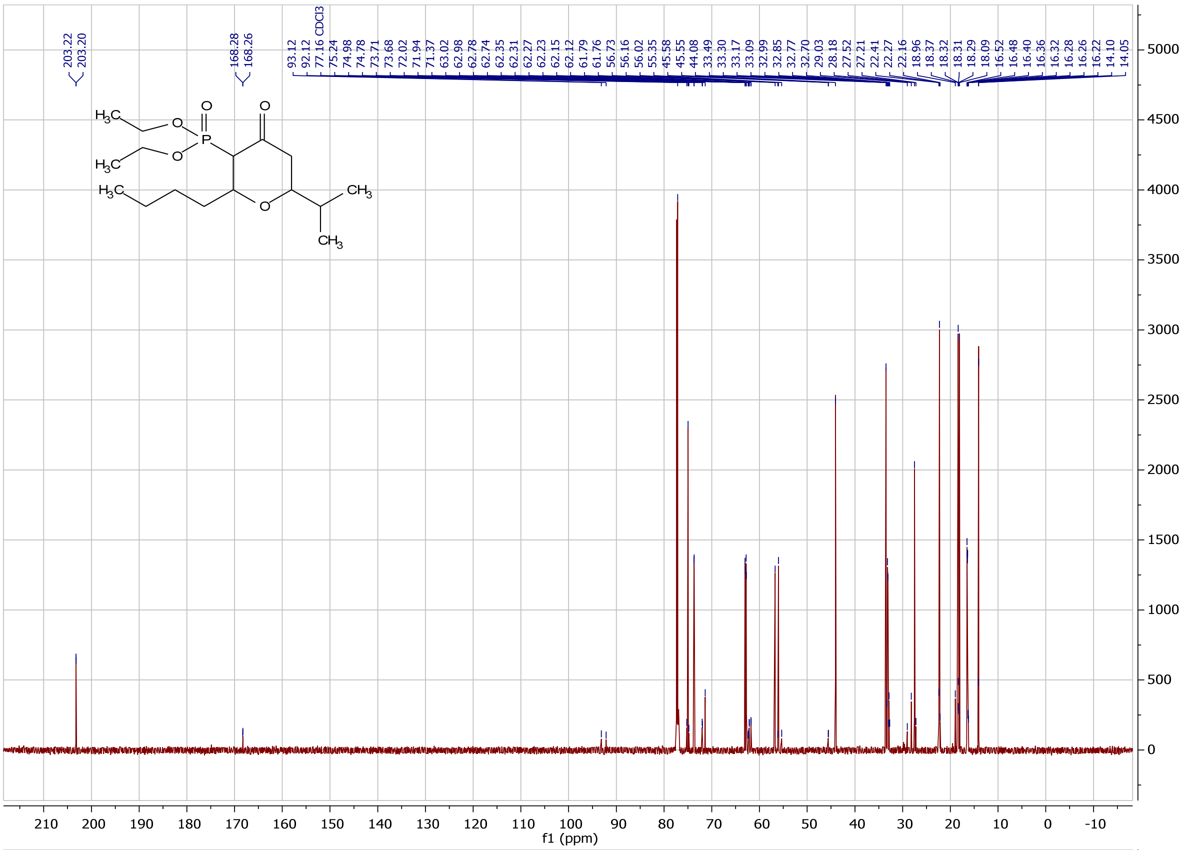

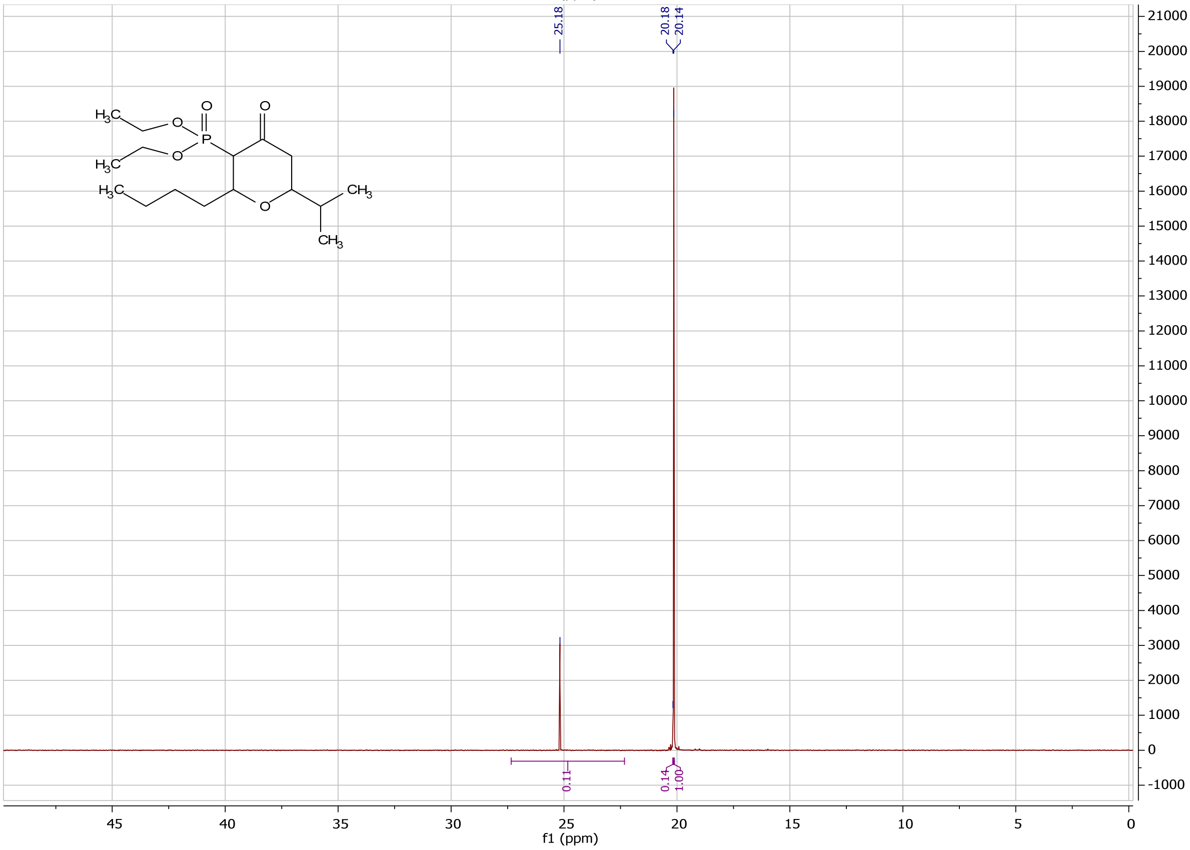

3-Diethoxyphosphoryl-6-isopropyl-2-phenyltetrahydropyran-4-one (**12d**)

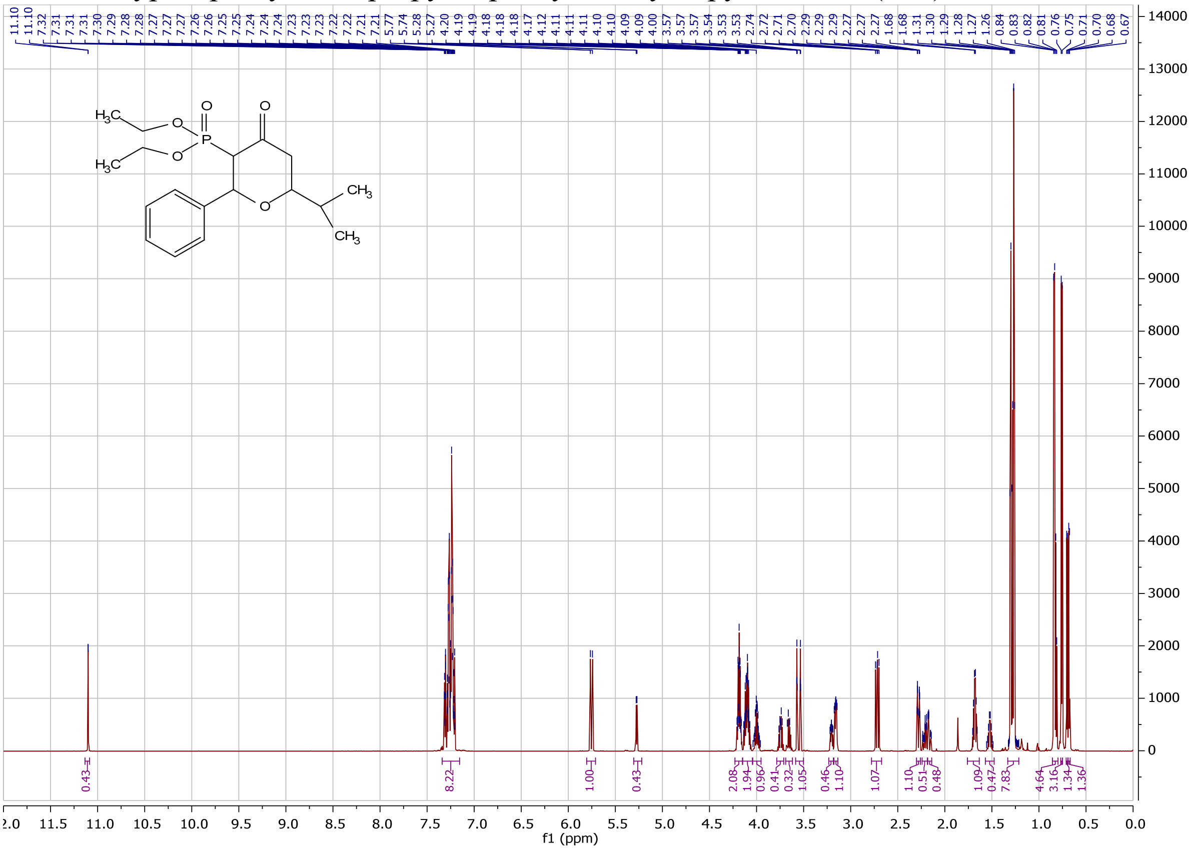

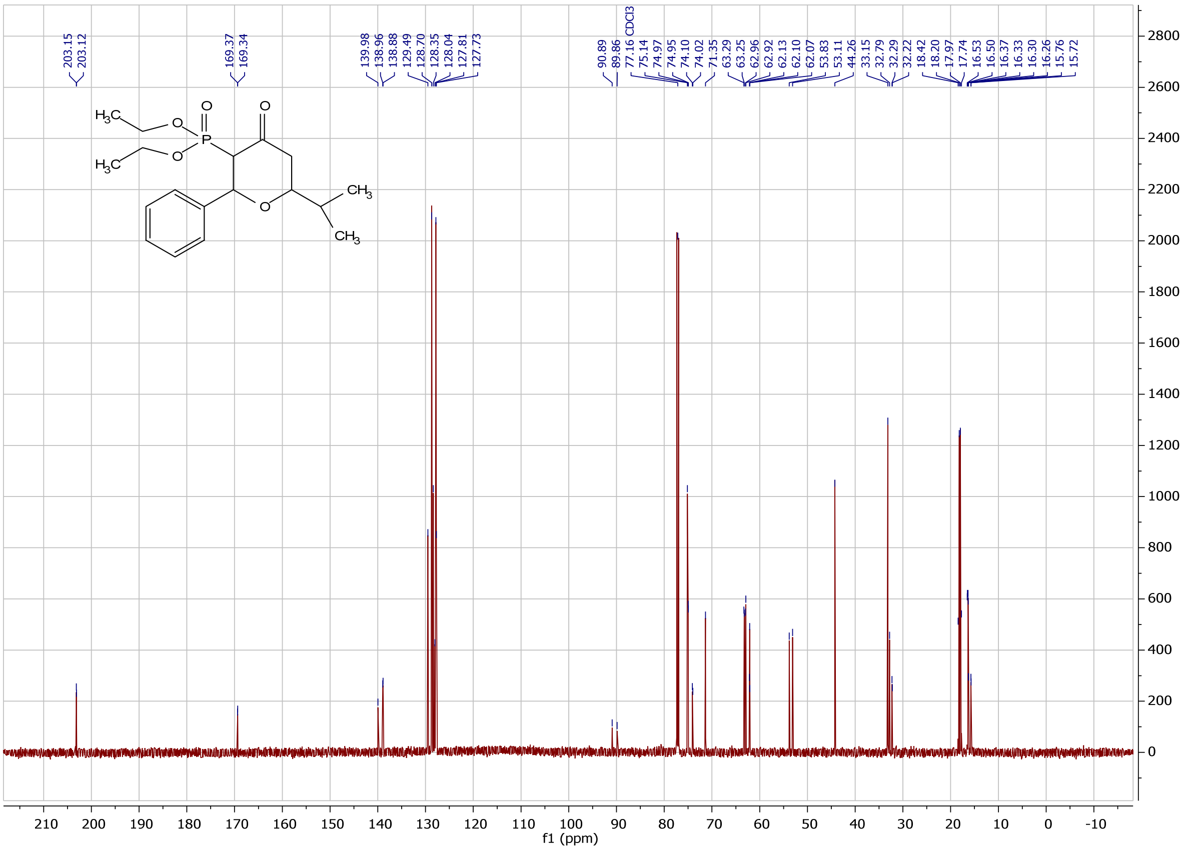

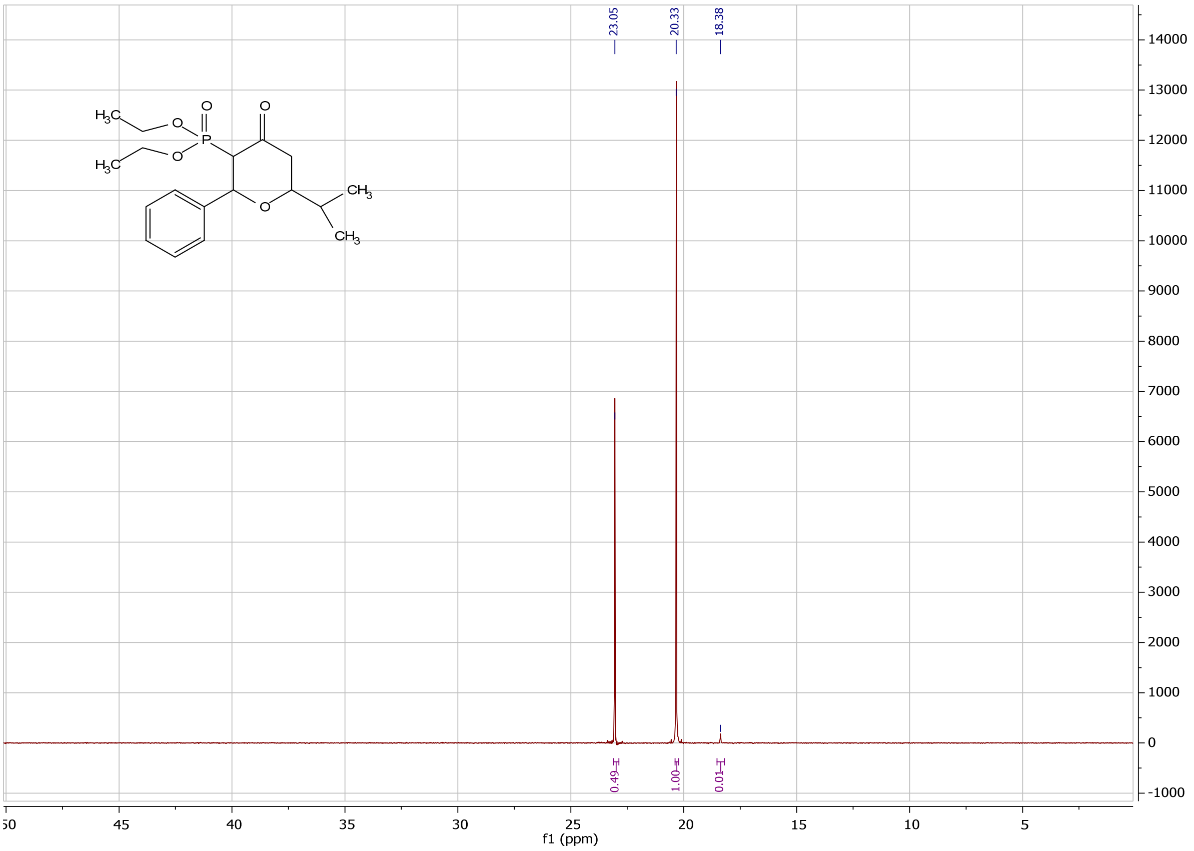

2-Butyl-3-diethoxyphosphoryl-6-phenyltetrahydropyran-4-one(**12e**)

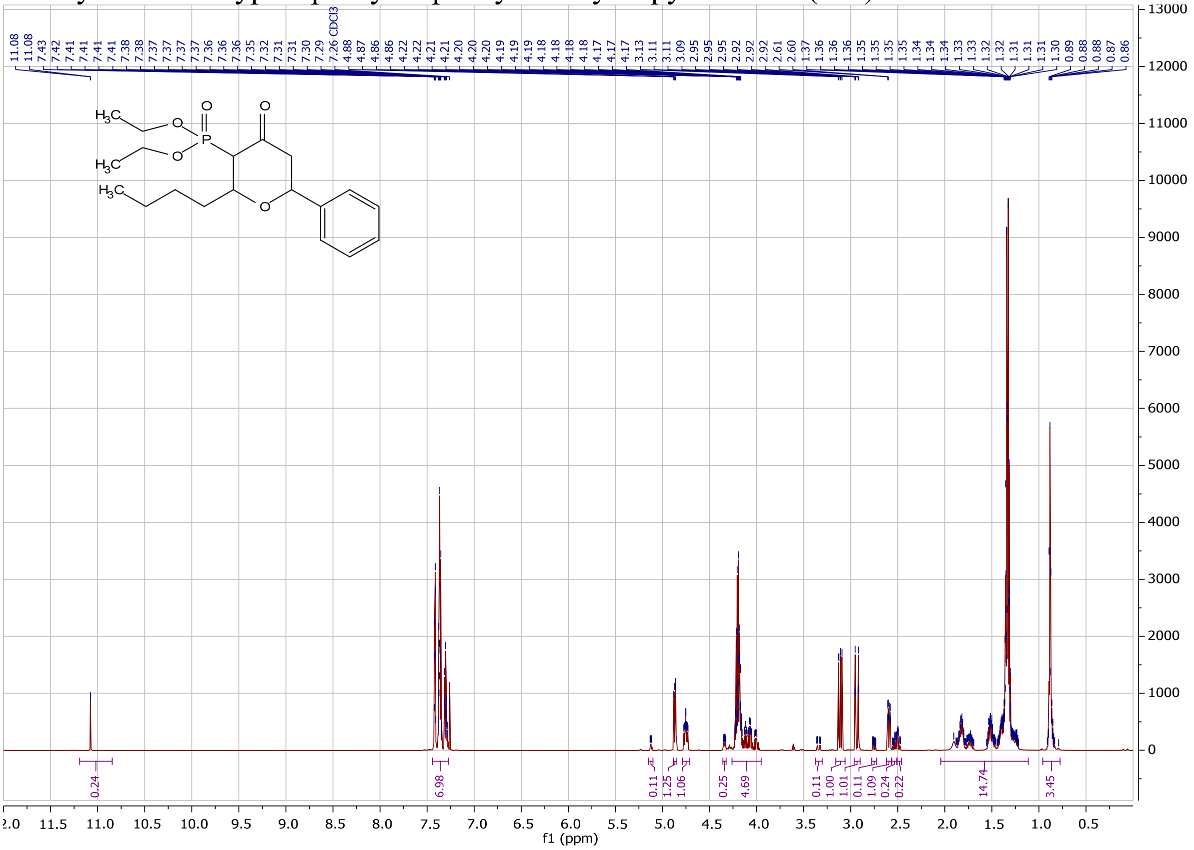

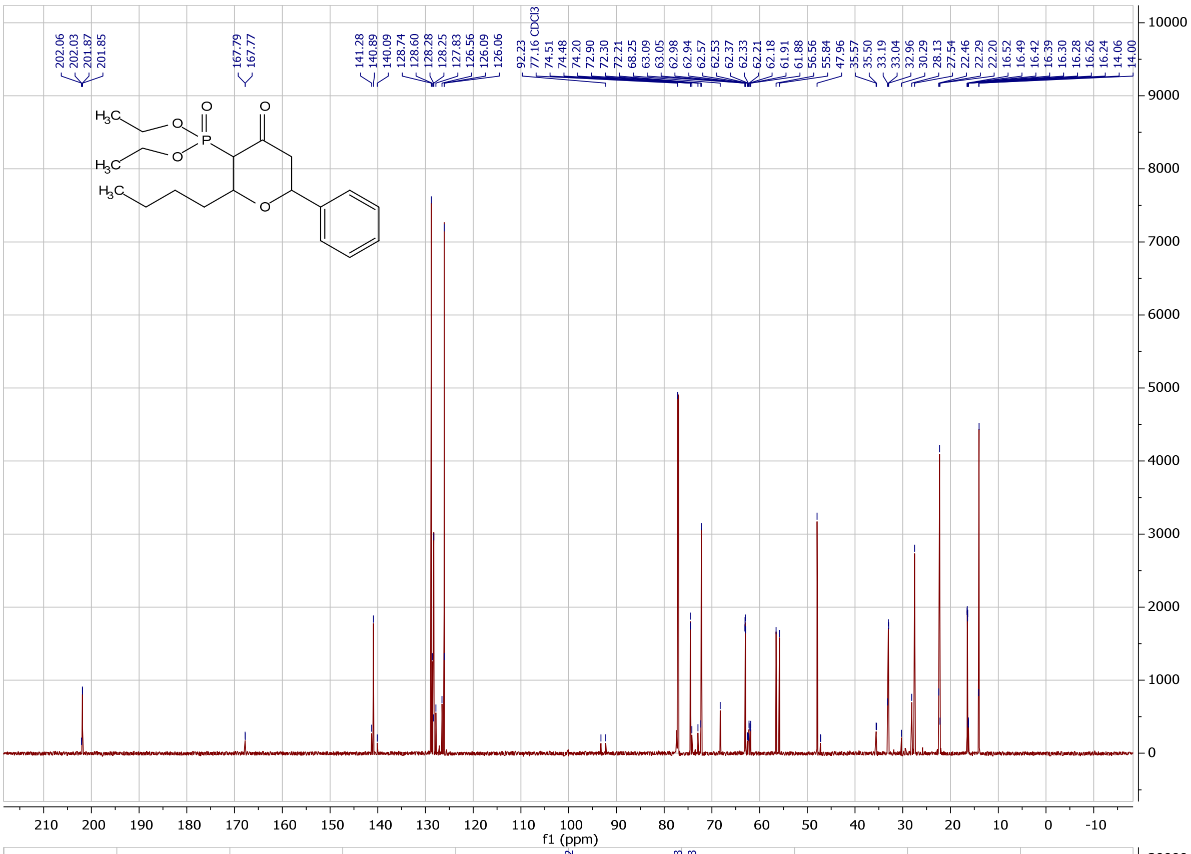

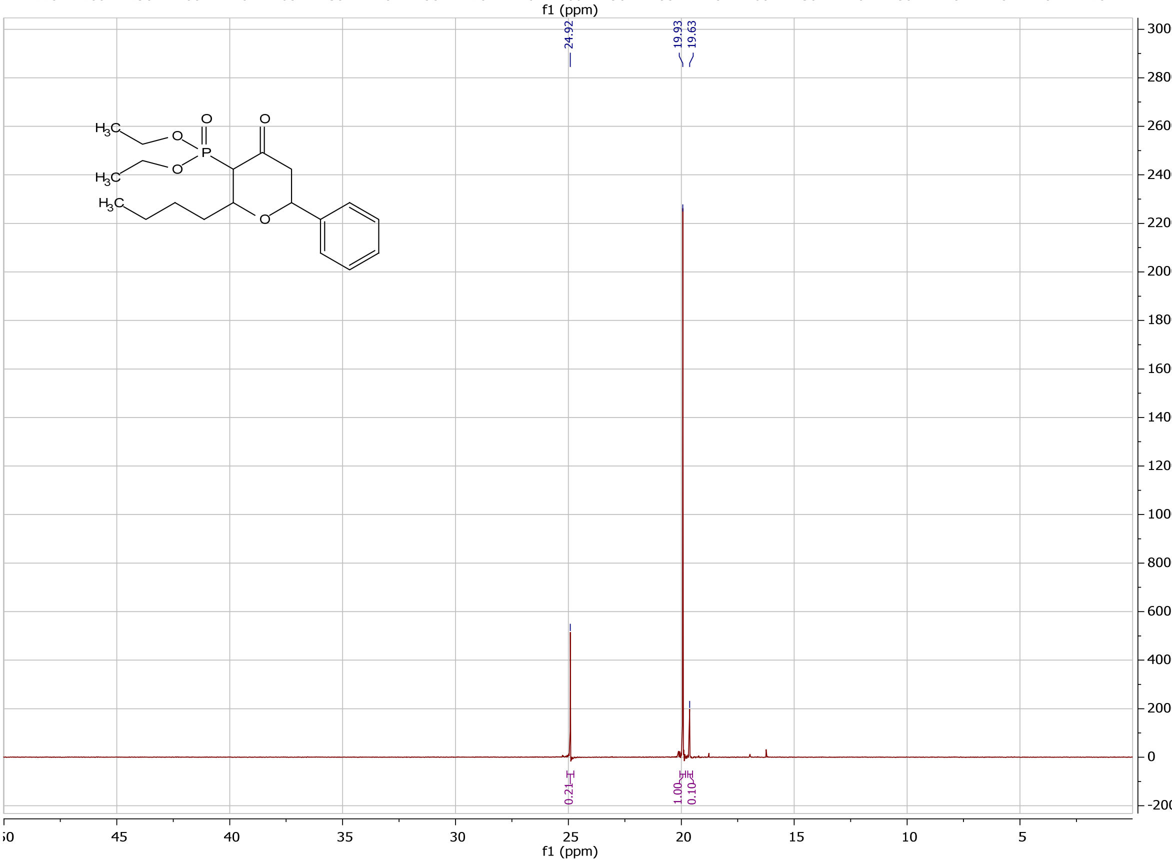

3-diethoxyphosphoryl-2,6-diphenyltetrahydropyran-4-one (**12f**)

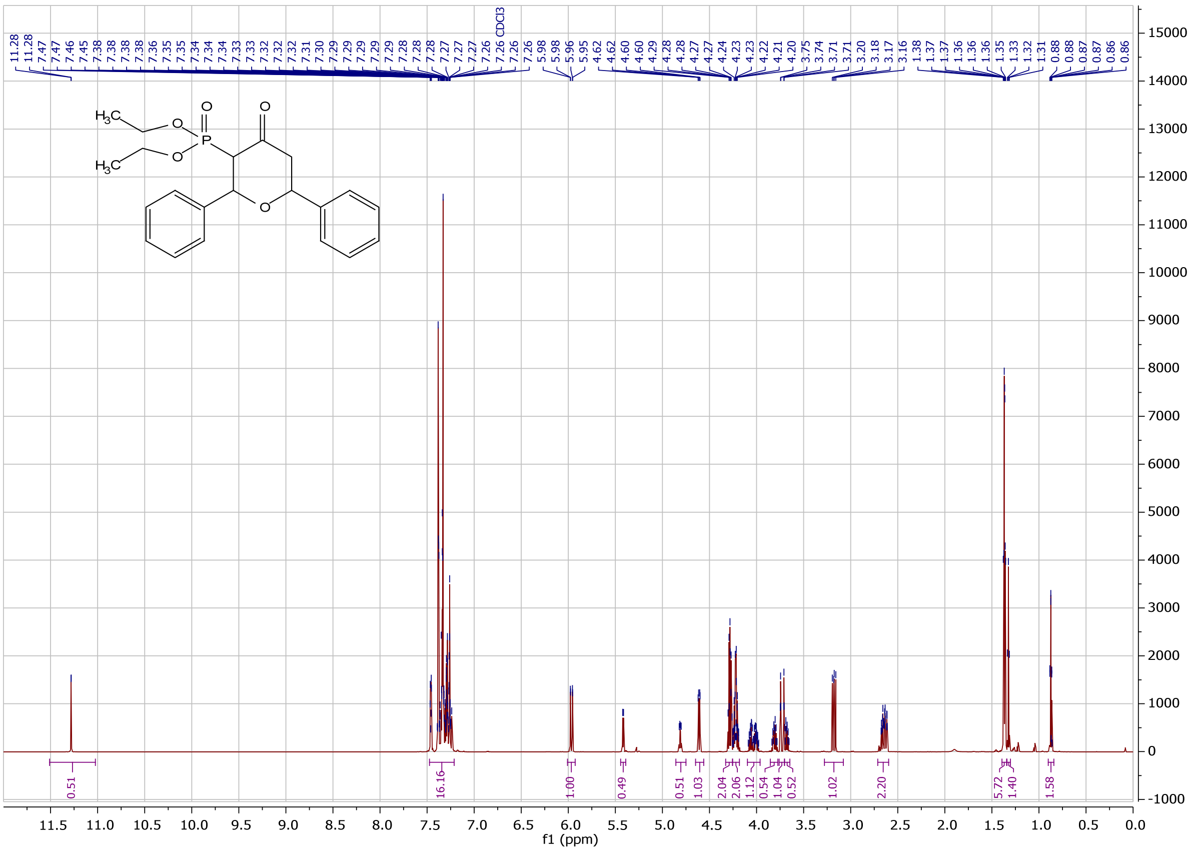

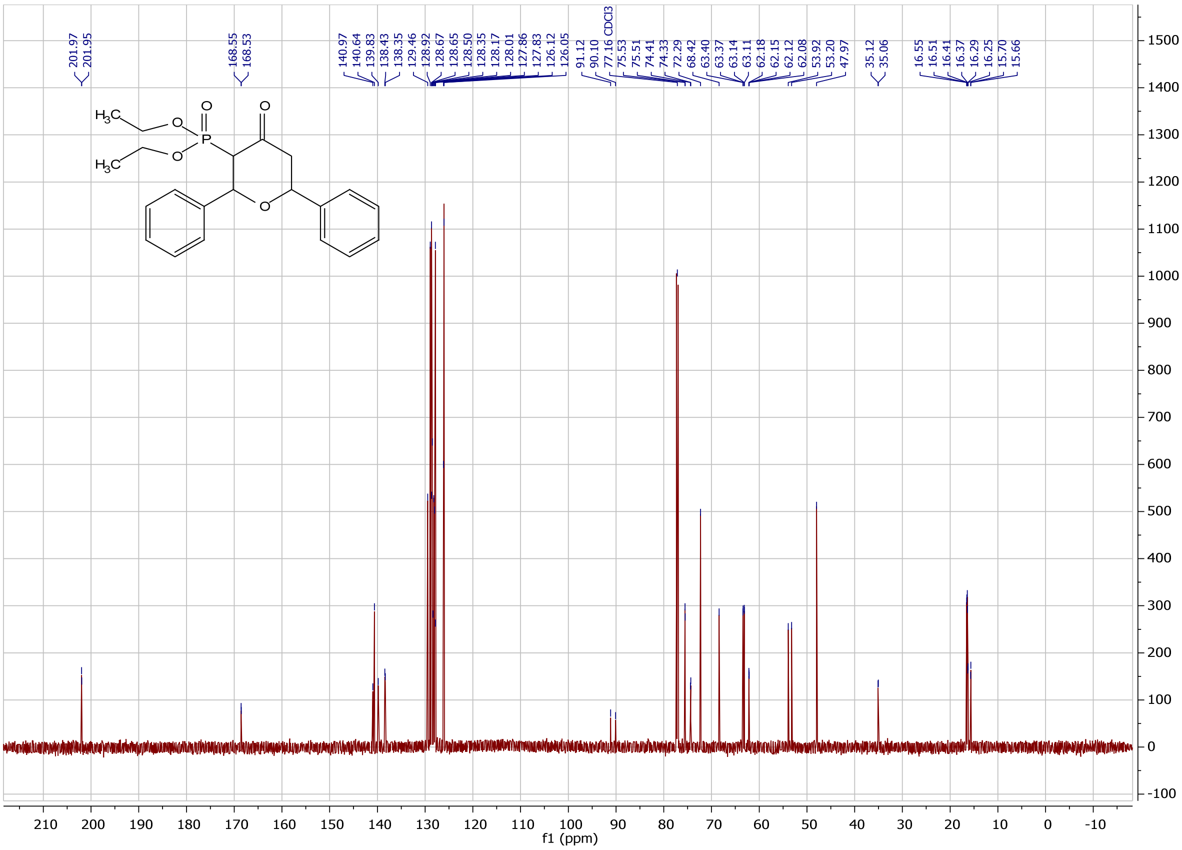

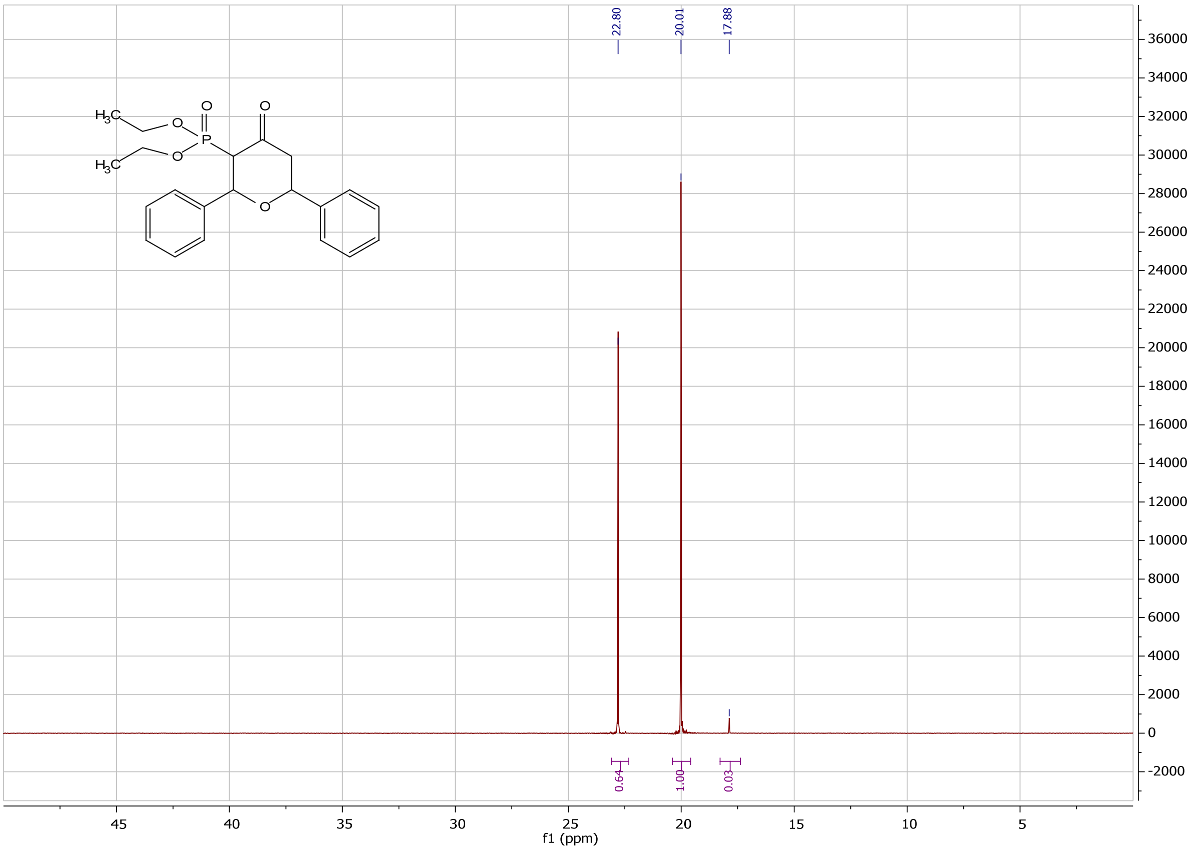

2-Butyl-3-diethoxyphosphoryl-6-(4-methoxyphenyl)tetrahydropyran-4-one (**12g**)

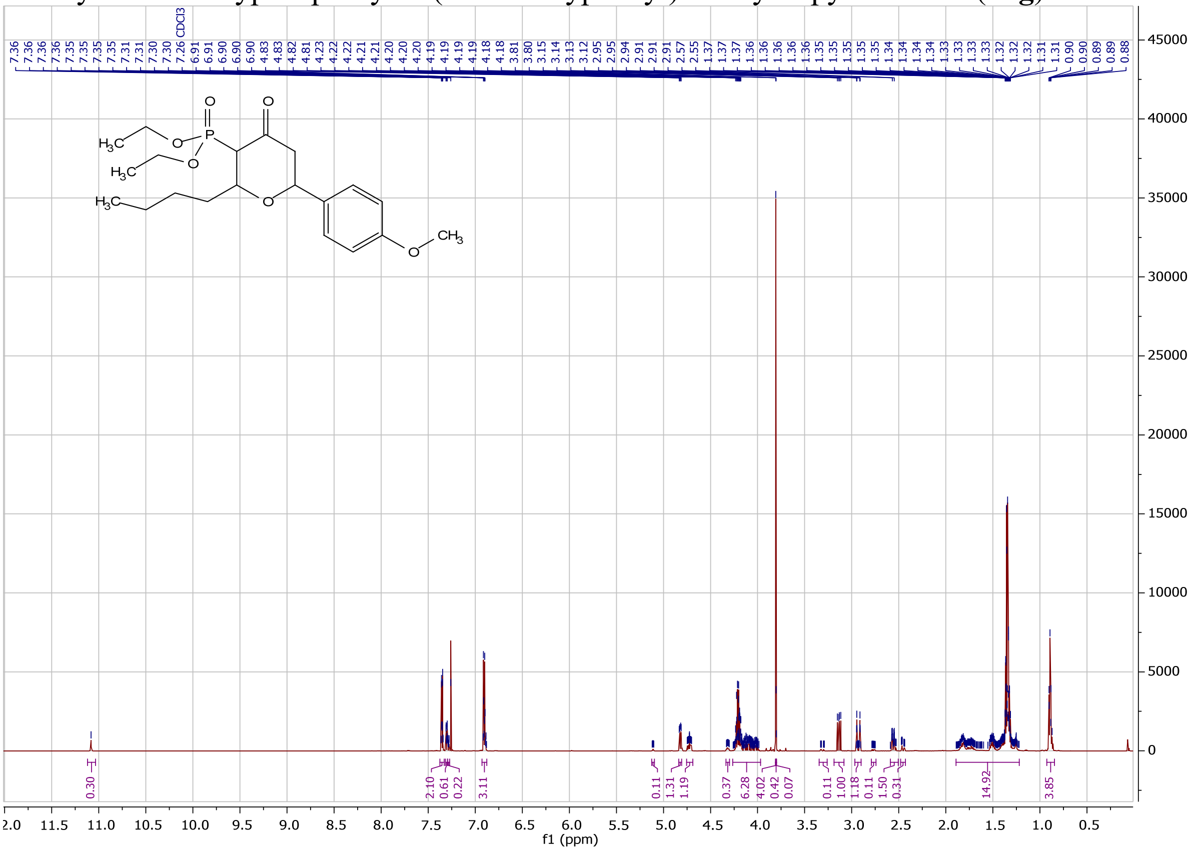

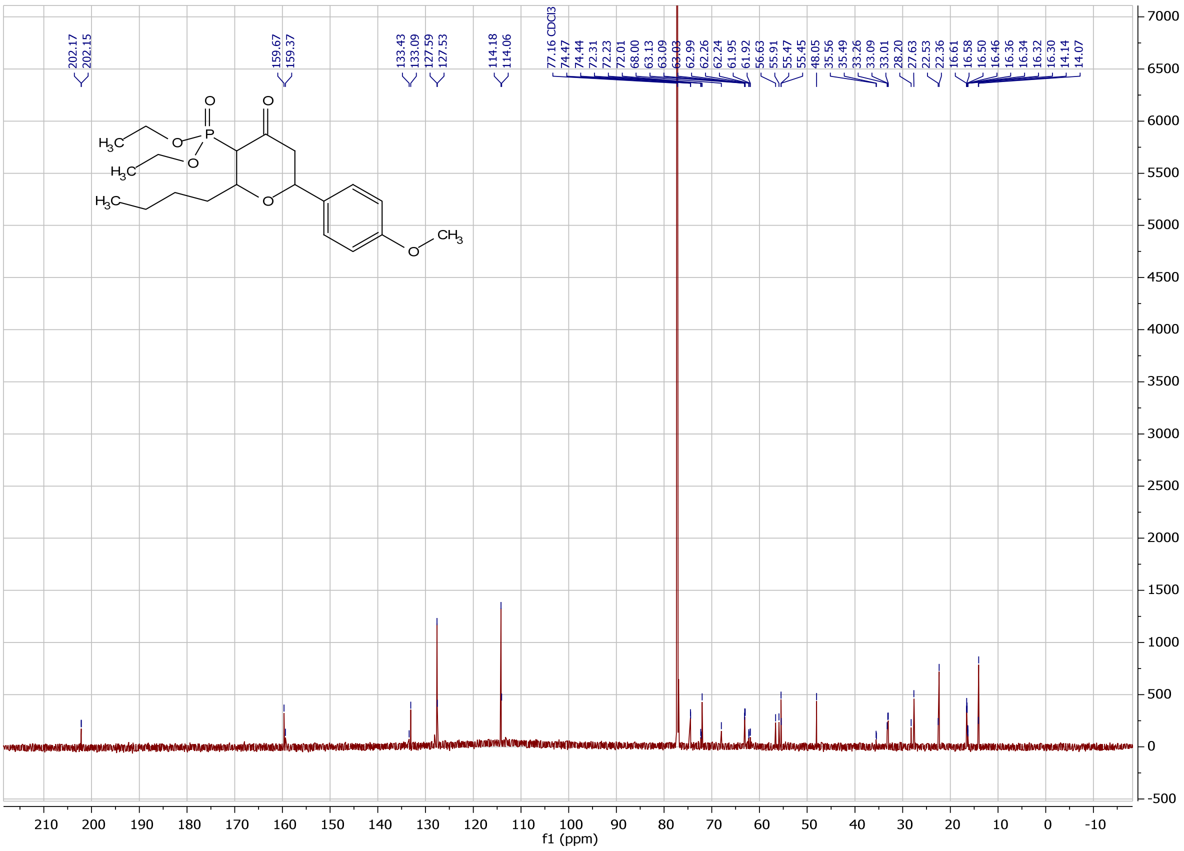

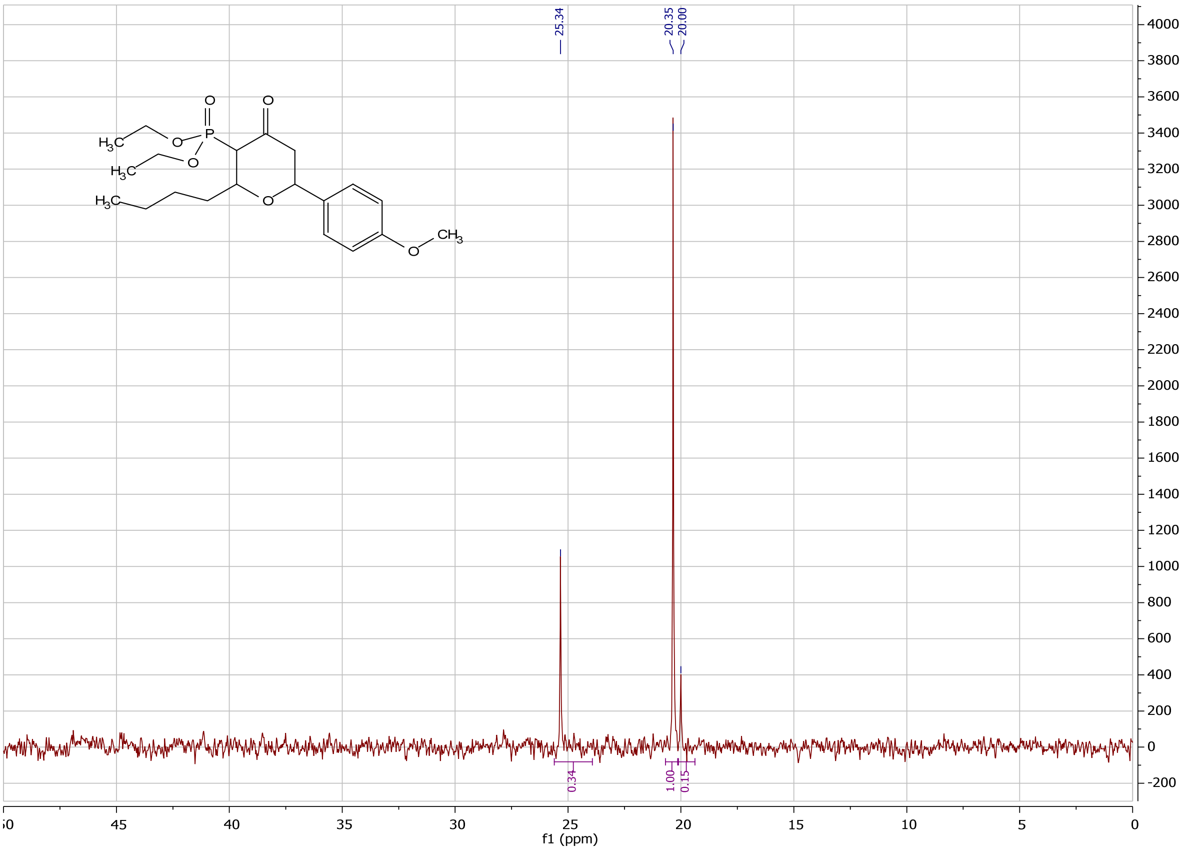

3-Diethoxyphosphoryl-6-(4-methoxyphenyl)-2-phenyltetrahydropyran-4-one (**12h**)

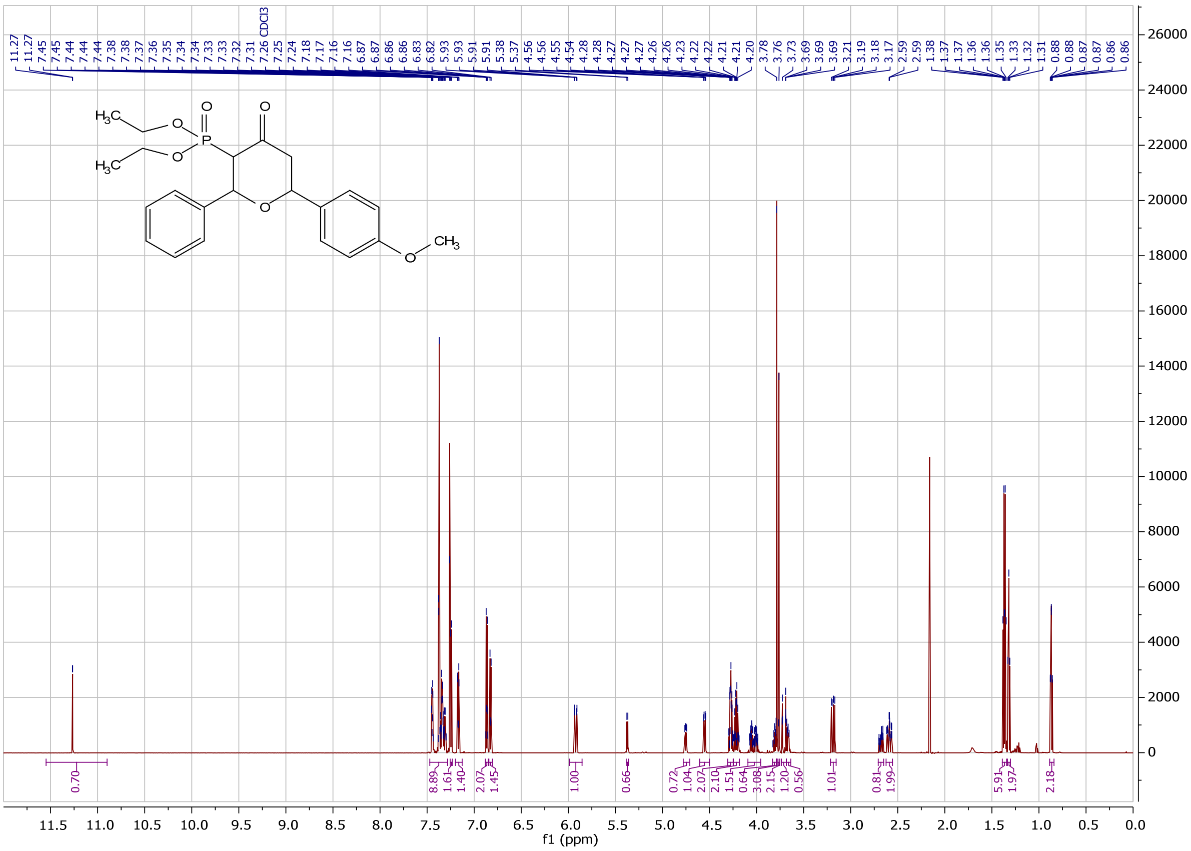
11.27
11.27
7.45
7.45
7.44
7.44
7.38
7.38
7.37
7.36
7.35
7.34
7.34
7.33
7.33
7.32
7.31
7.26 CDCl3
7.25
7.24
7.18
7.17
7.16
7.16
6.87
6.87
6.86
6.86
6.83
6.82
5.93
5.93
5.91
5.91
5.38
5.37
4.56
4.56
4.55
4.54
4.28
4.28
4.27
4.27
4.27
4.26
4.26
4.23
4.22
4.22
4.21
4.21
4.20
3.78
3.76
3.73
3.69
3.69
3.69
3.21
3.19
3.18
3.17
2.59
2.59
1.38
1.37
1.37
1.36
1.36
1.35
1.33
1.32
1.31
0.88
0.88
0.87
0.87
0.86
0.86
H3C
H3C
O
O
P
O
O
O
CH3
O
0.70
8.89
1.61
1.40
2.07
1.45
1.00
0.66
0.72
1.04
2.07
2.10
1.51
0.64
3.08
2.15
1.20
0.56
1.01
0.81
1.99
5.91
1.97
2.18
f1 (ppm)

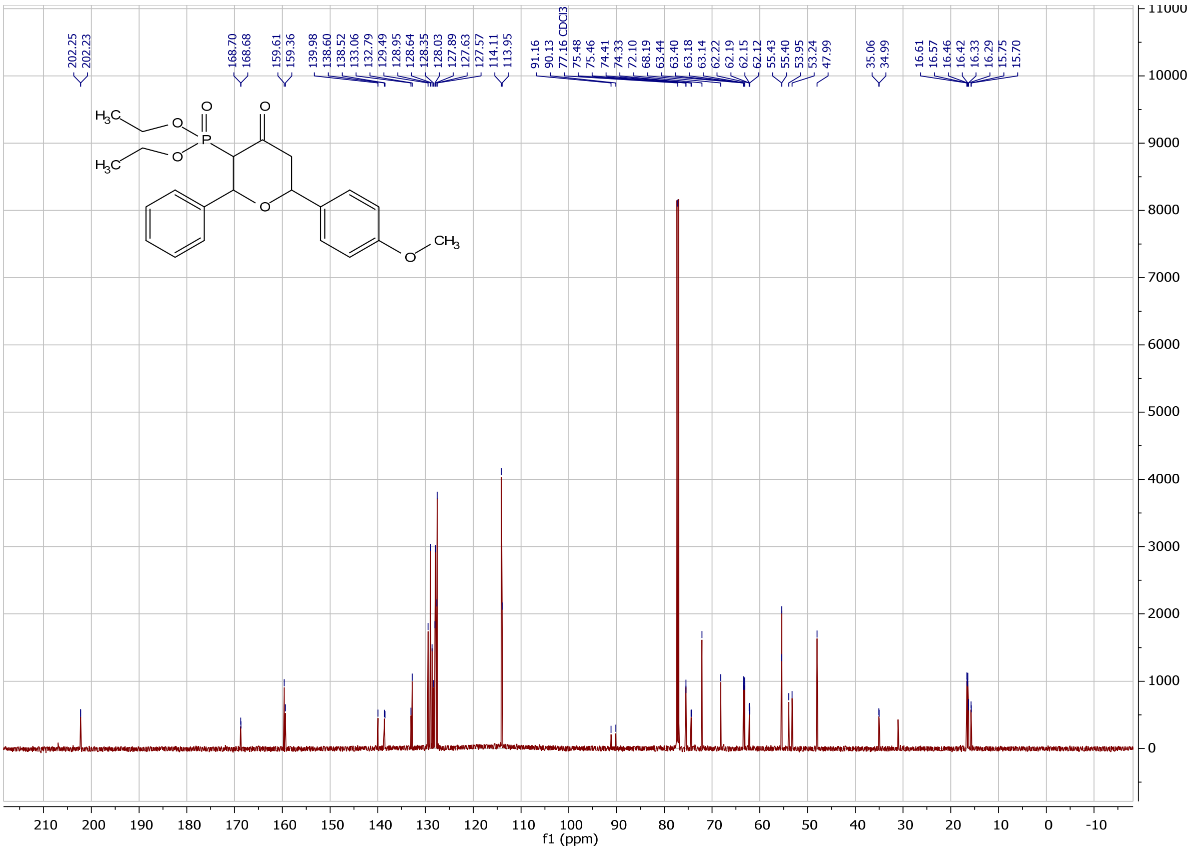
202.25
202.23
168.70
168.68
159.61
159.36
139.98
138.60
138.52
133.06
132.79
129.49
128.95
128.64
128.35
128.03
127.89
127.63
127.57
114.11
113.95
91.16
90.13
77.16 CDCl3
75.48
75.46
74.41
74.33
72.10
68.19
63.44
63.40
63.18
63.14
62.22
62.19
62.15
62.12
55.43
55.40
53.95
53.24
47.99
35.06
34.99
16.61
16.57
16.46
16.42
16.33
16.29
15.75
15.70
H3C
H3C
O
O
P
O
O
O
CH3
O
f1 (ppm)

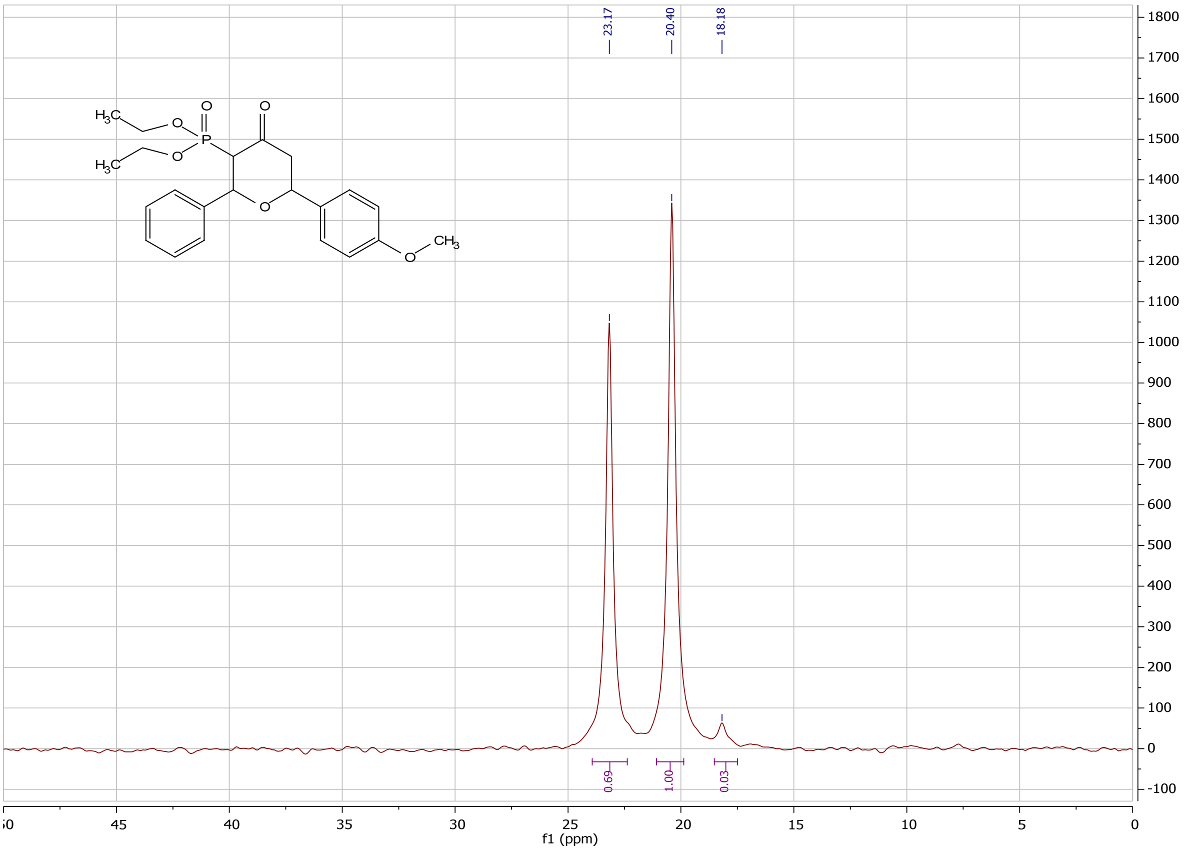

2-Butyl-3-diethoxyphosphoryl-6-ferrocenyltetrahydropyran-4-one (**12i**)

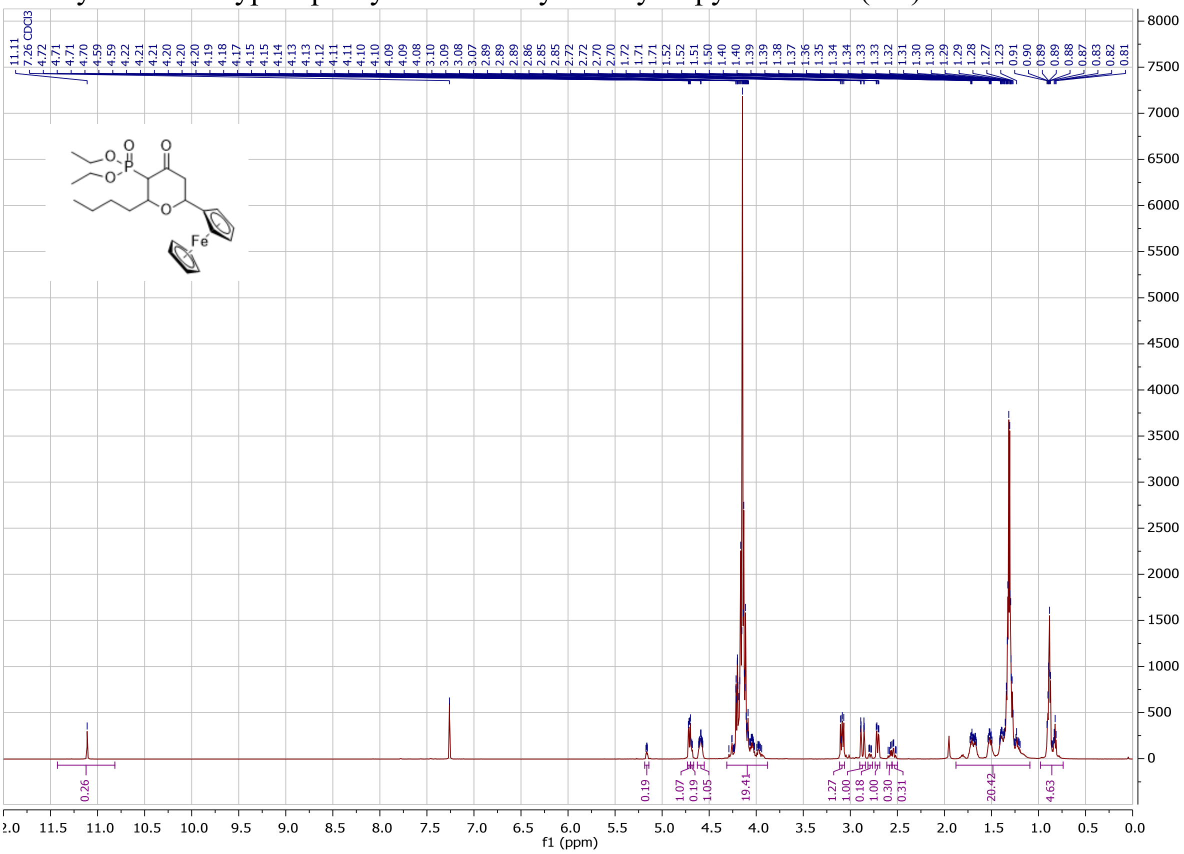

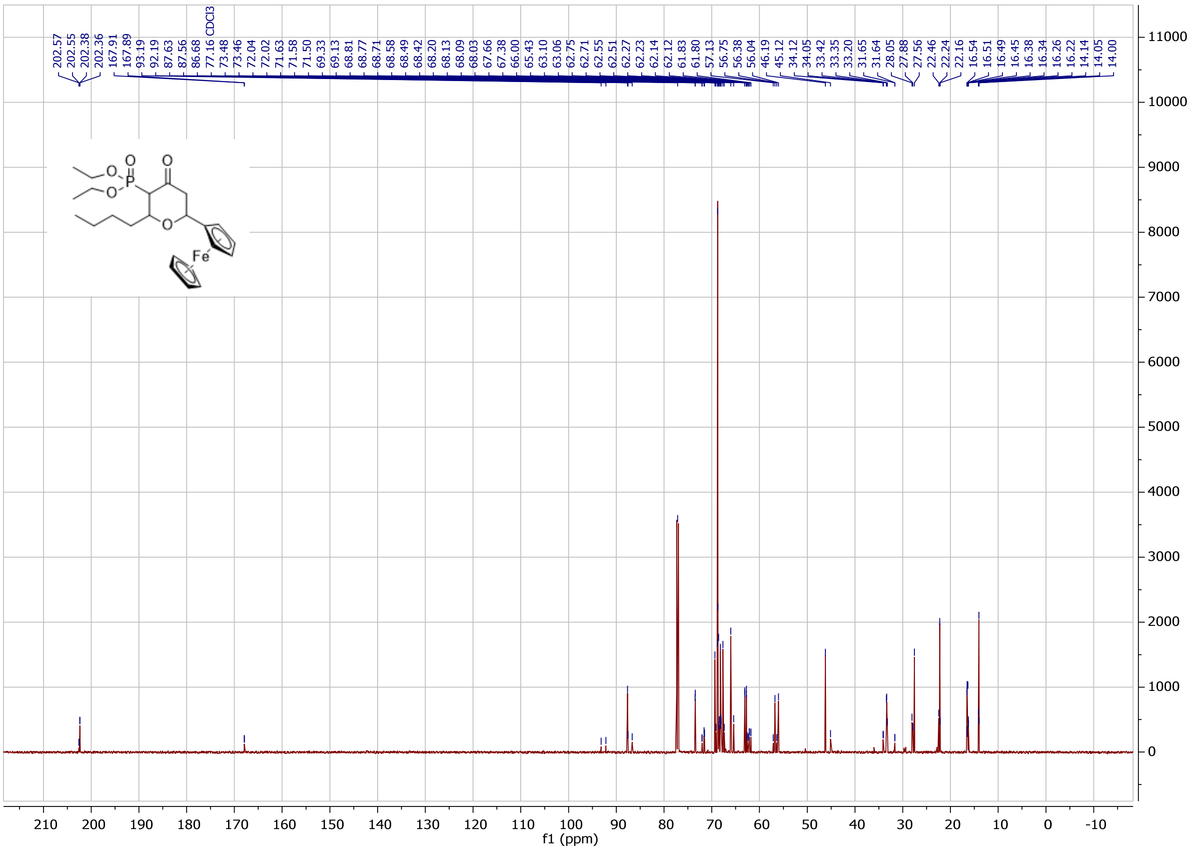

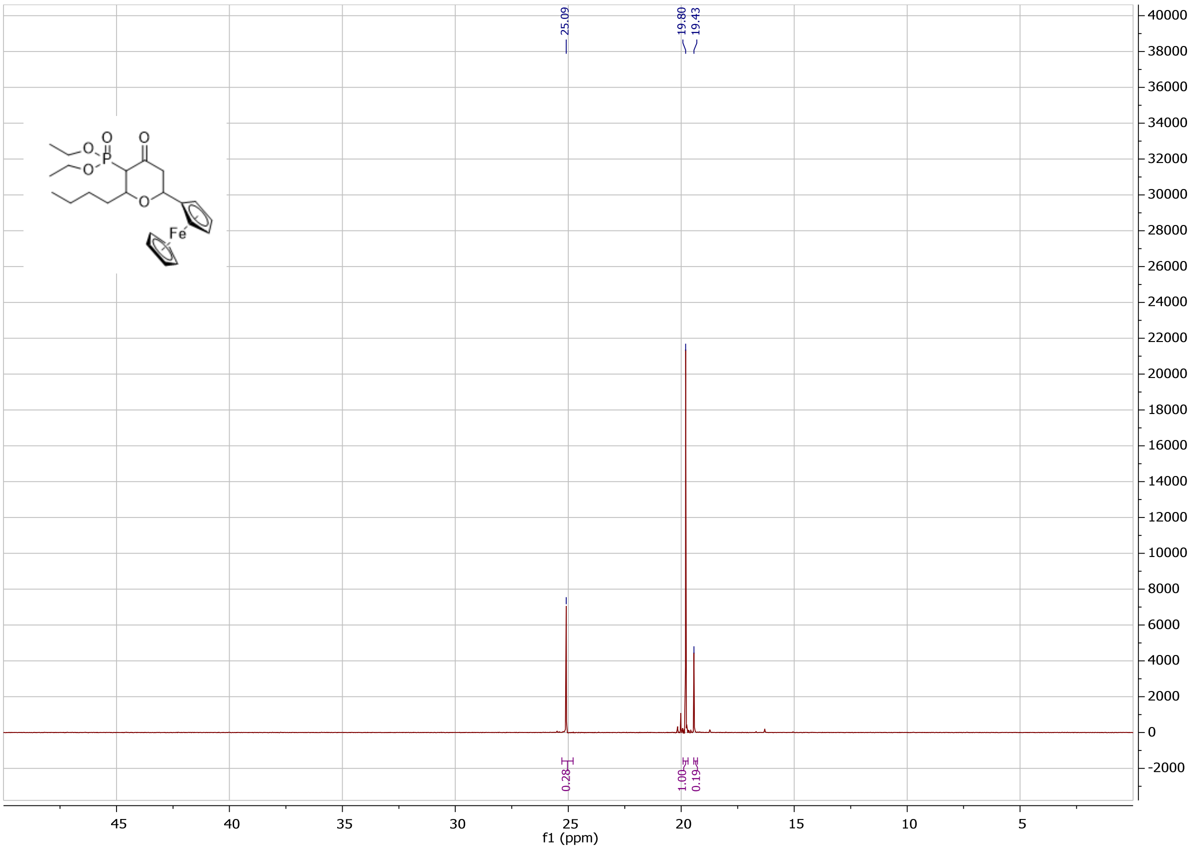

3-diethoxyphosphoryl-6-ferrocenyl-2-phenyltetrahydropyran-4-one (**12j**)

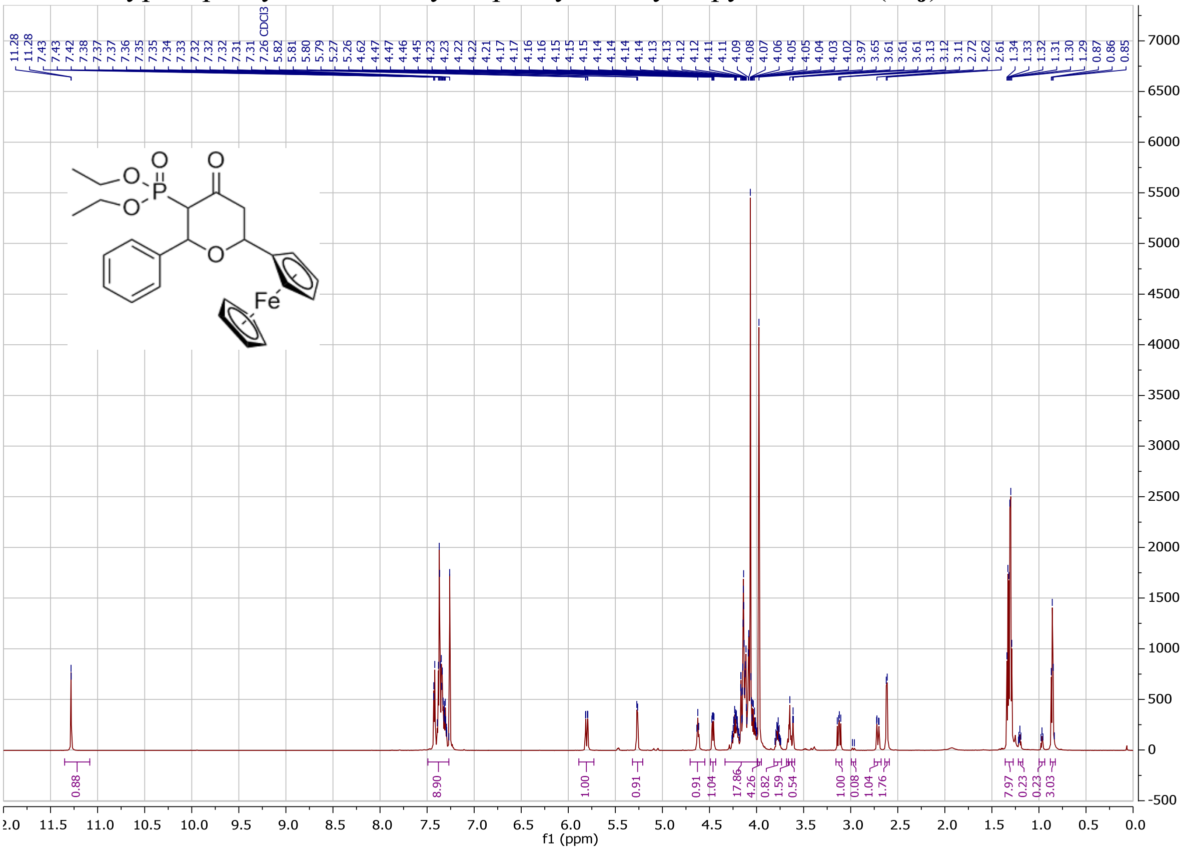

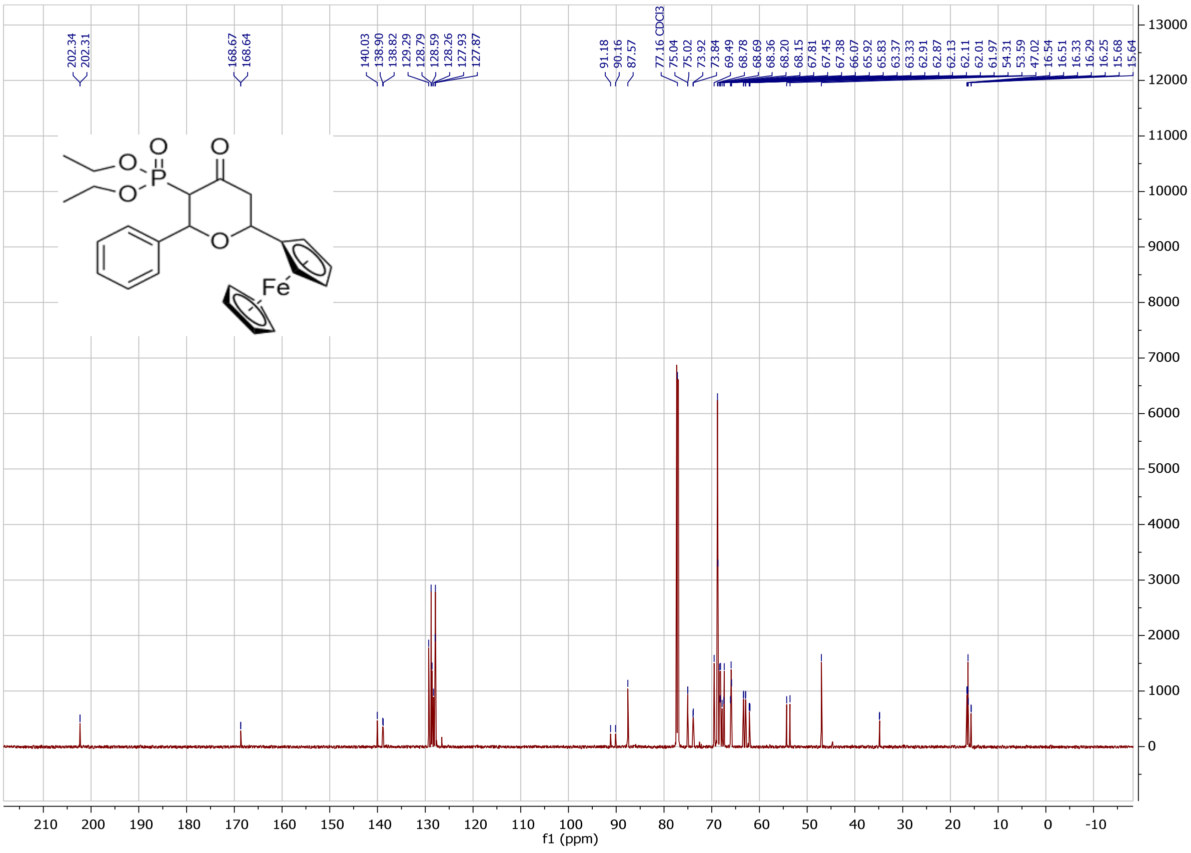

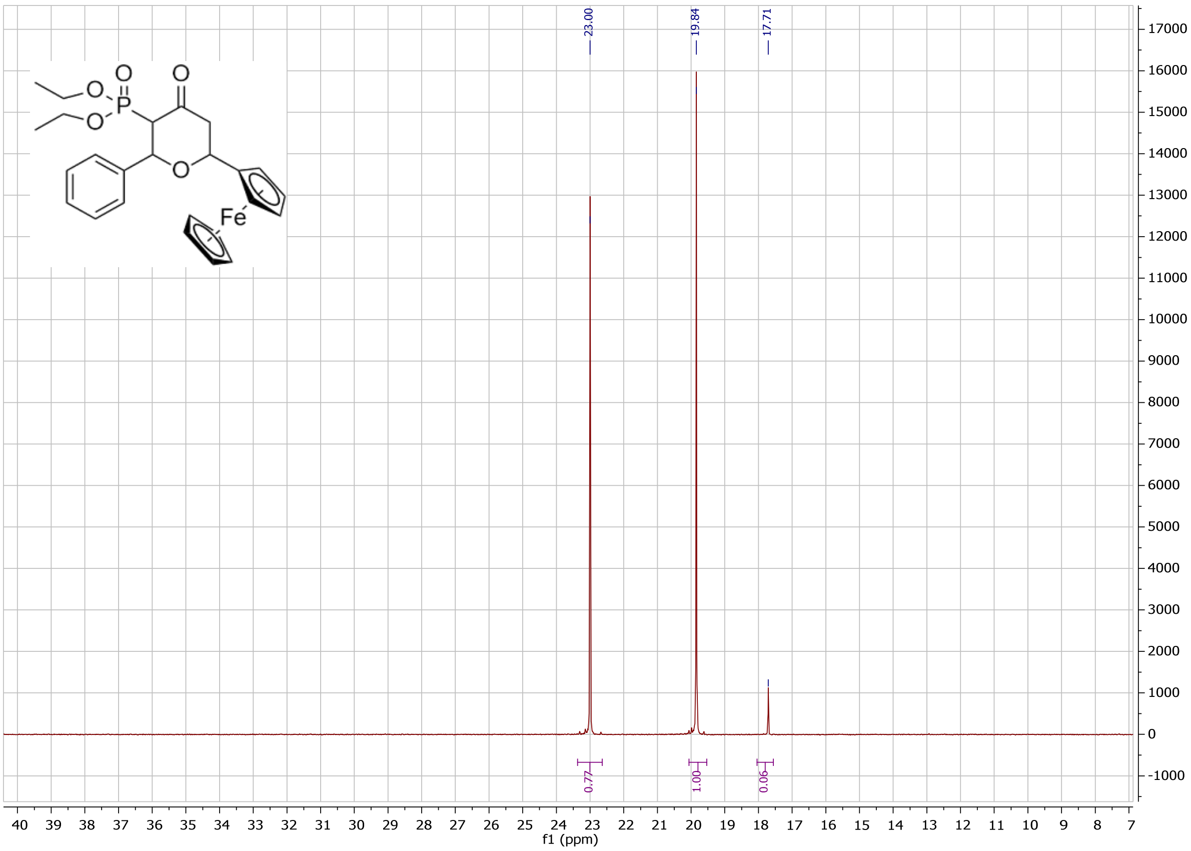

3-Diethoxyphosphoryl-6-ethyl-2-isopropyltetrahydropyran-4-one (**12k**)

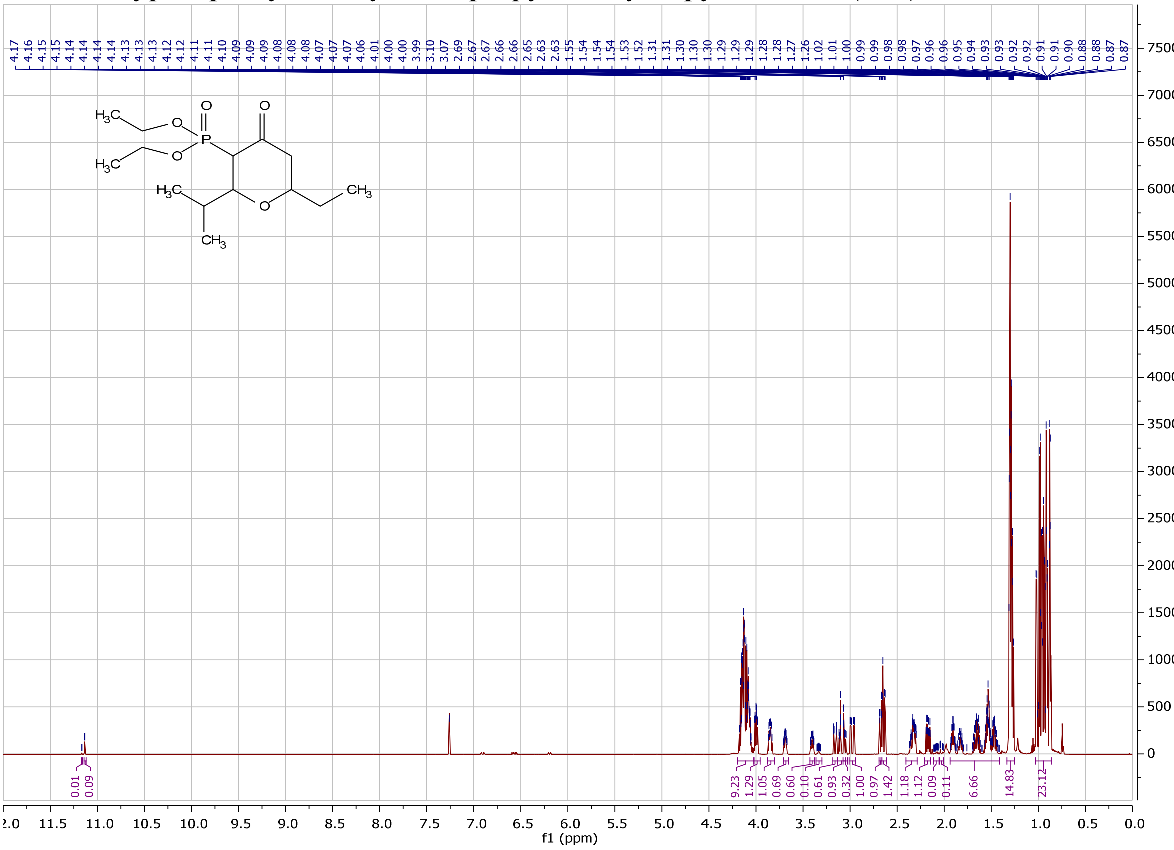

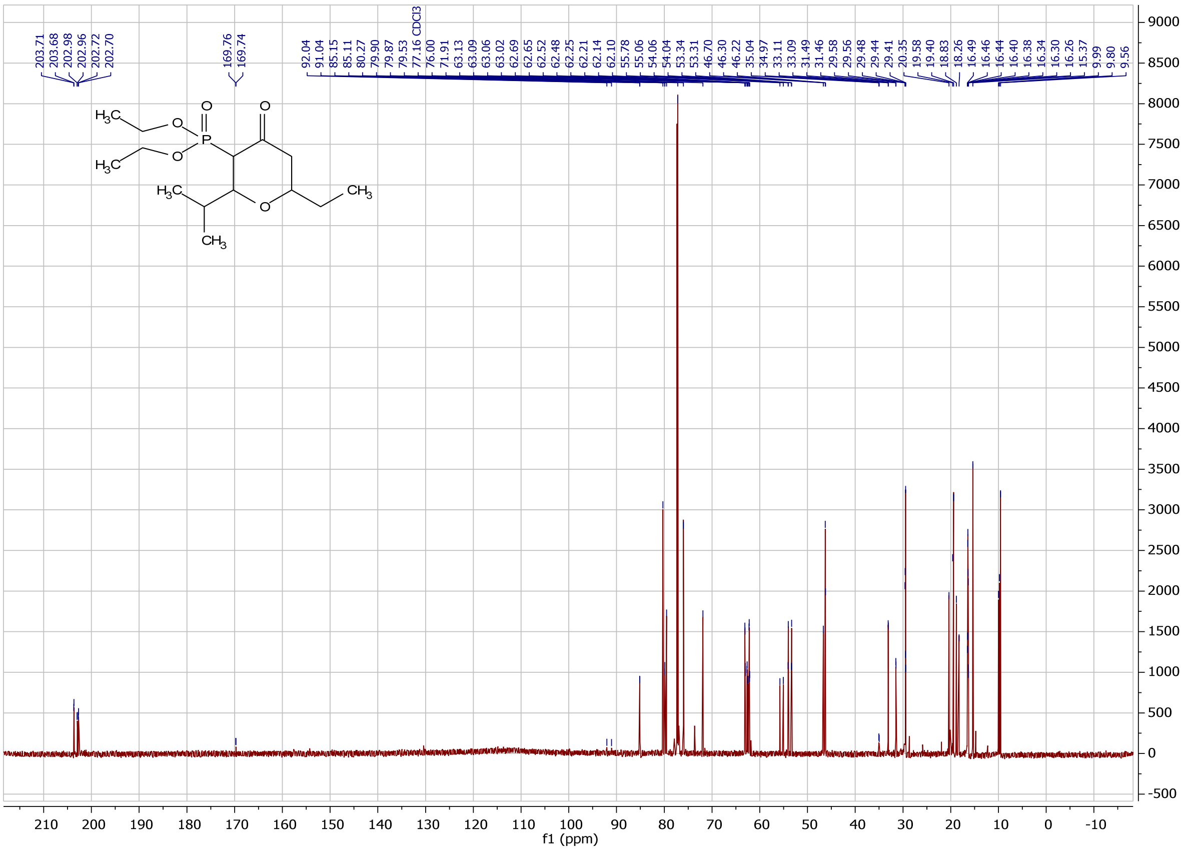

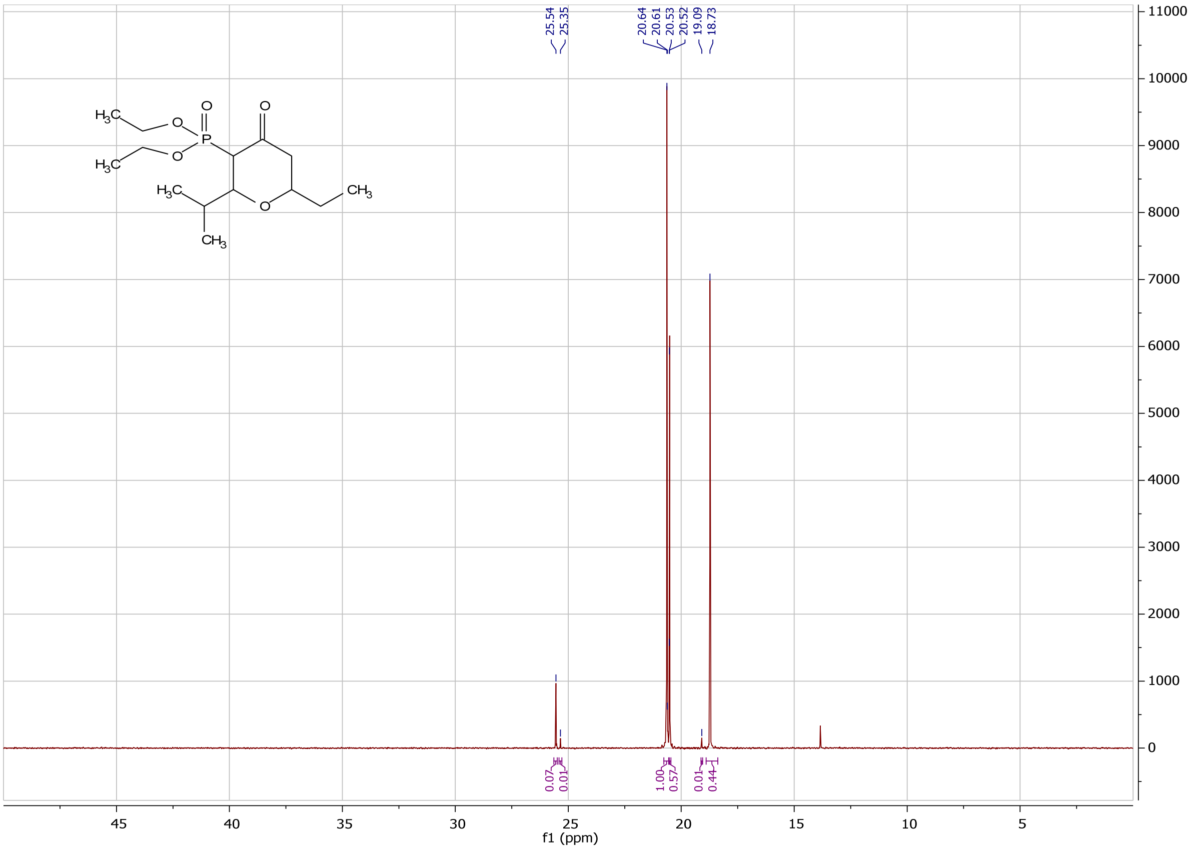

3-Diethoxyphosphoryl-2,6-diisopropyltetrahydropyran-4-one (**12l**)

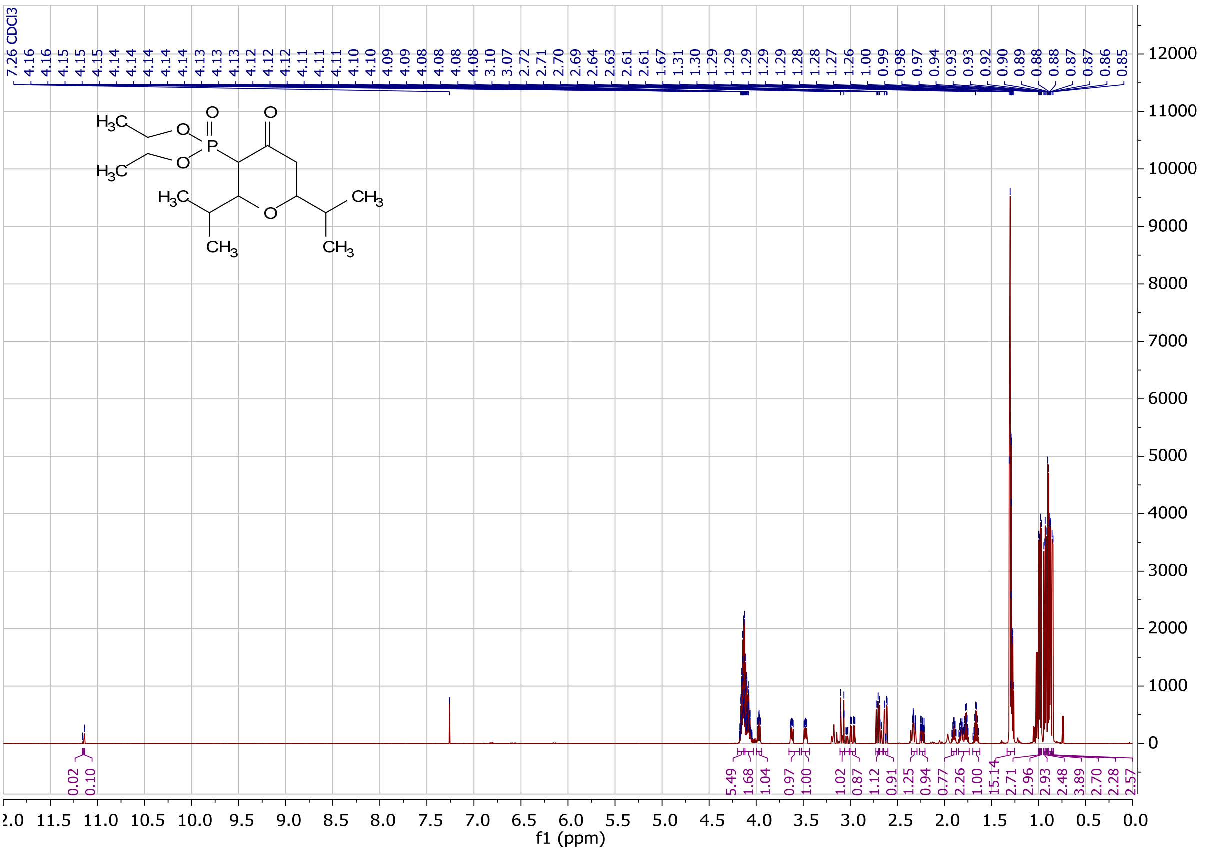

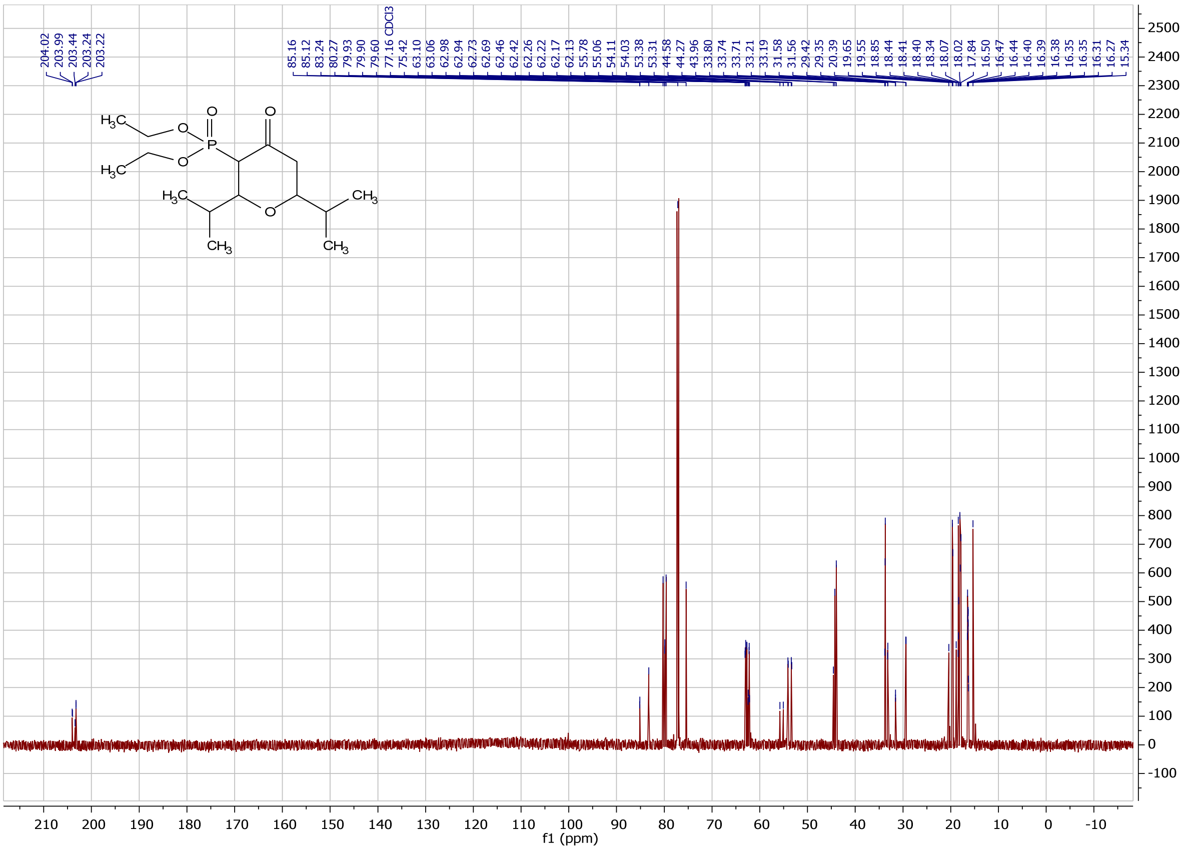

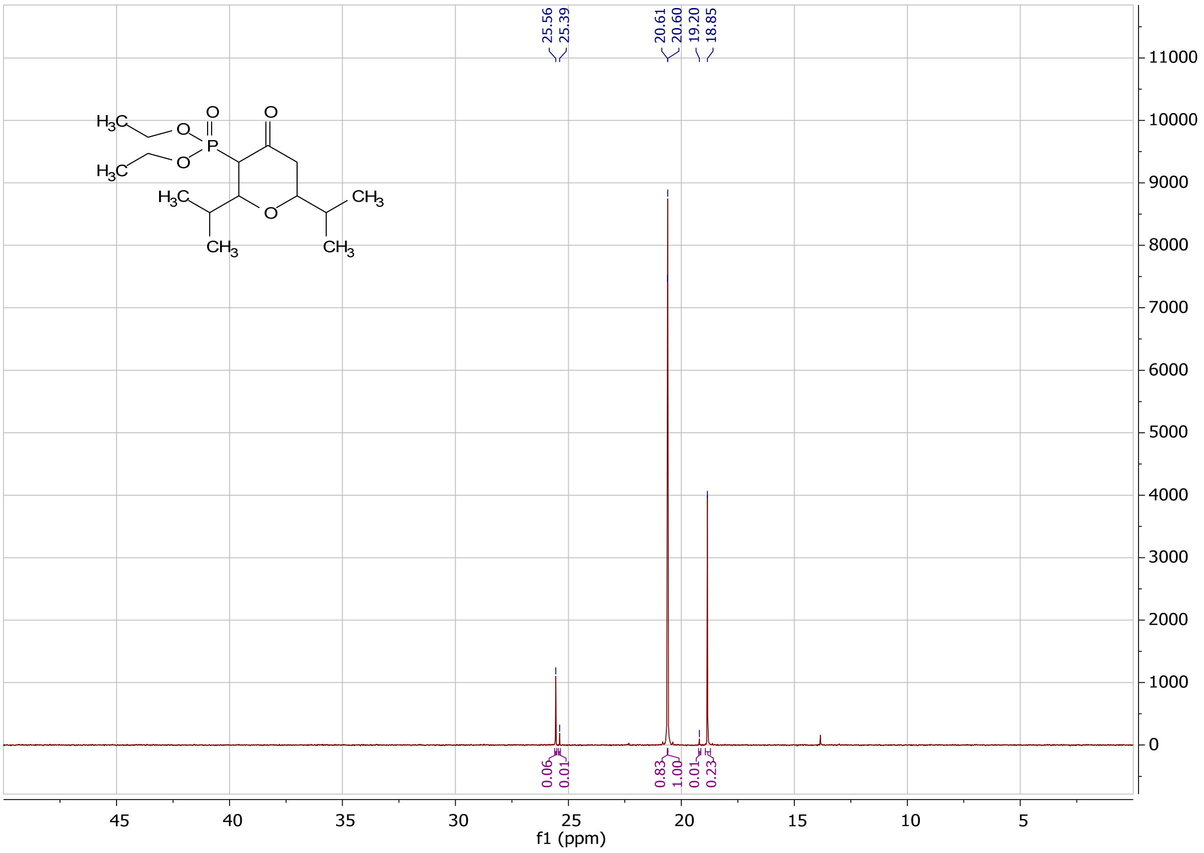

3-Diethoxyphosphoryl-2-isopropyl-6-phenyltetrahydropyran-4-one (**12m**)

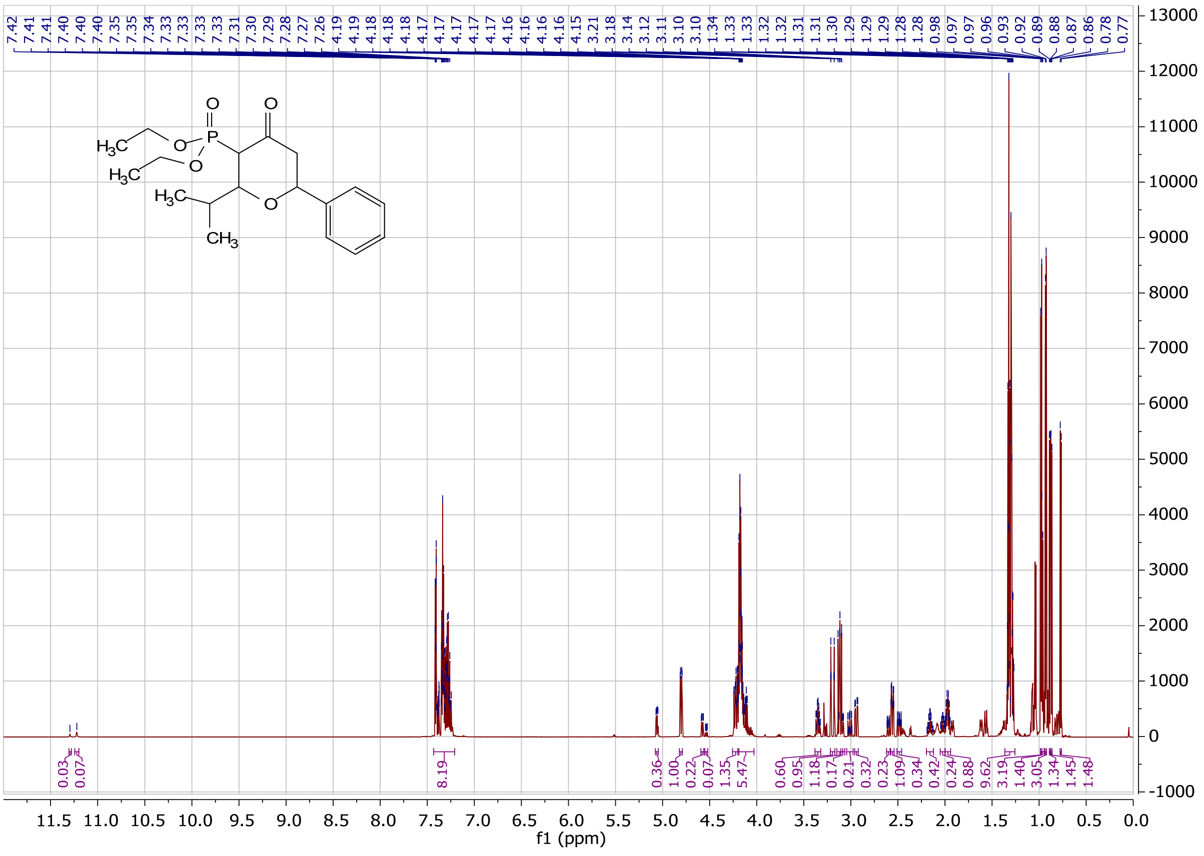

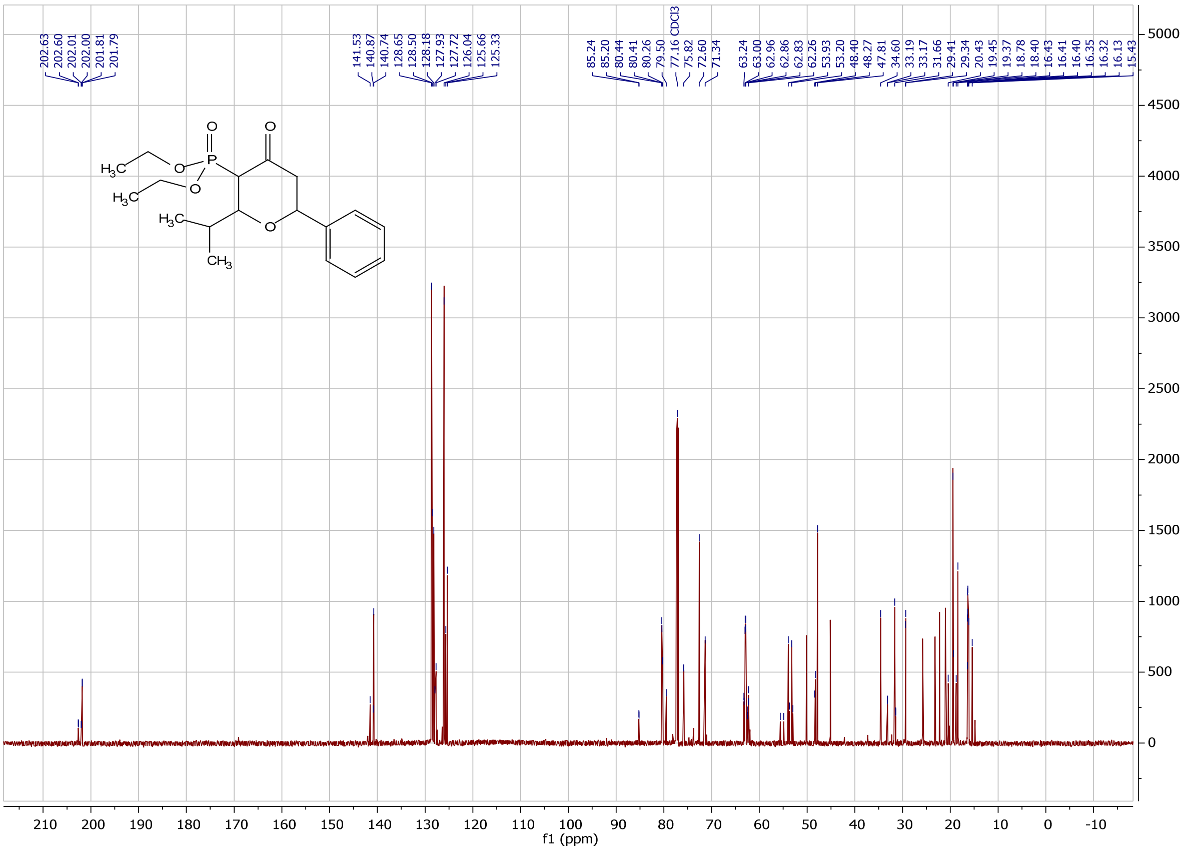

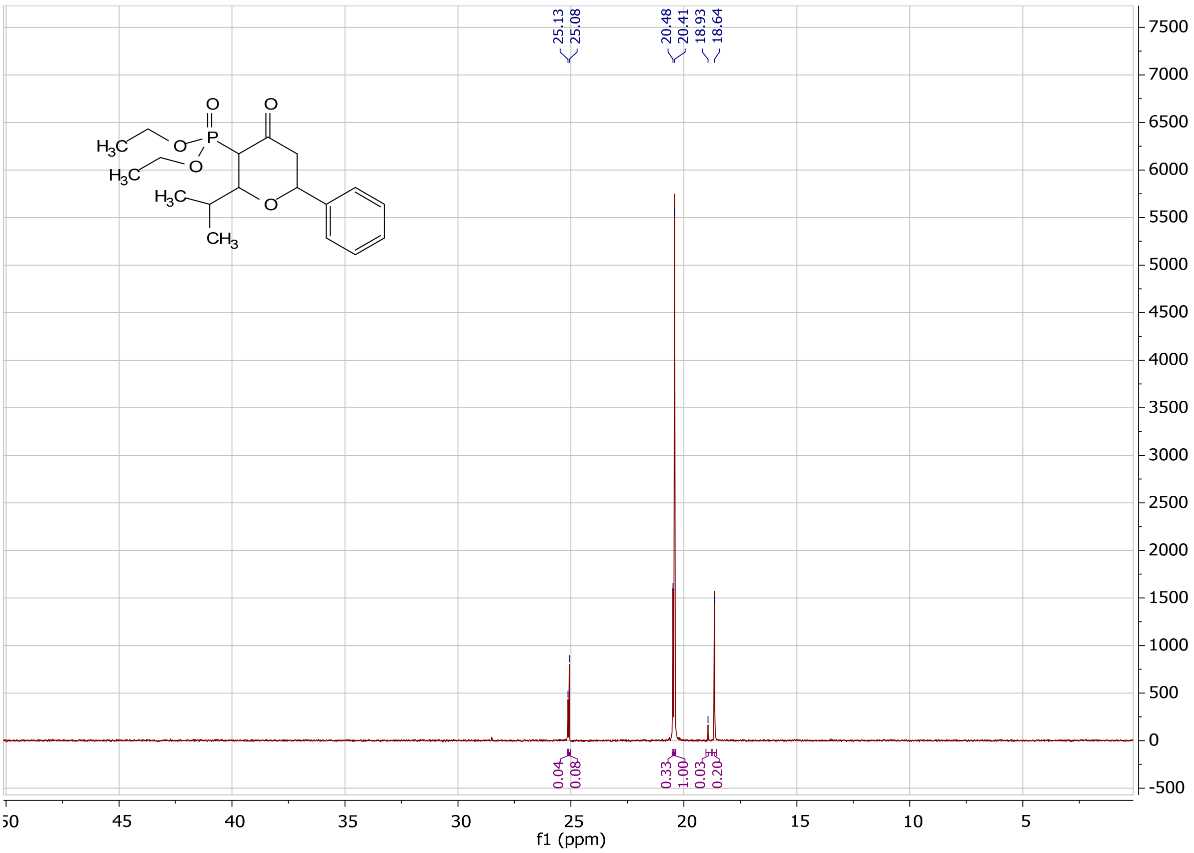

3-Diethoxyphosphoryl-2-isopropyl-6-(4-methoxyphenyl)tetrahydropyran-4-one (**12n**)

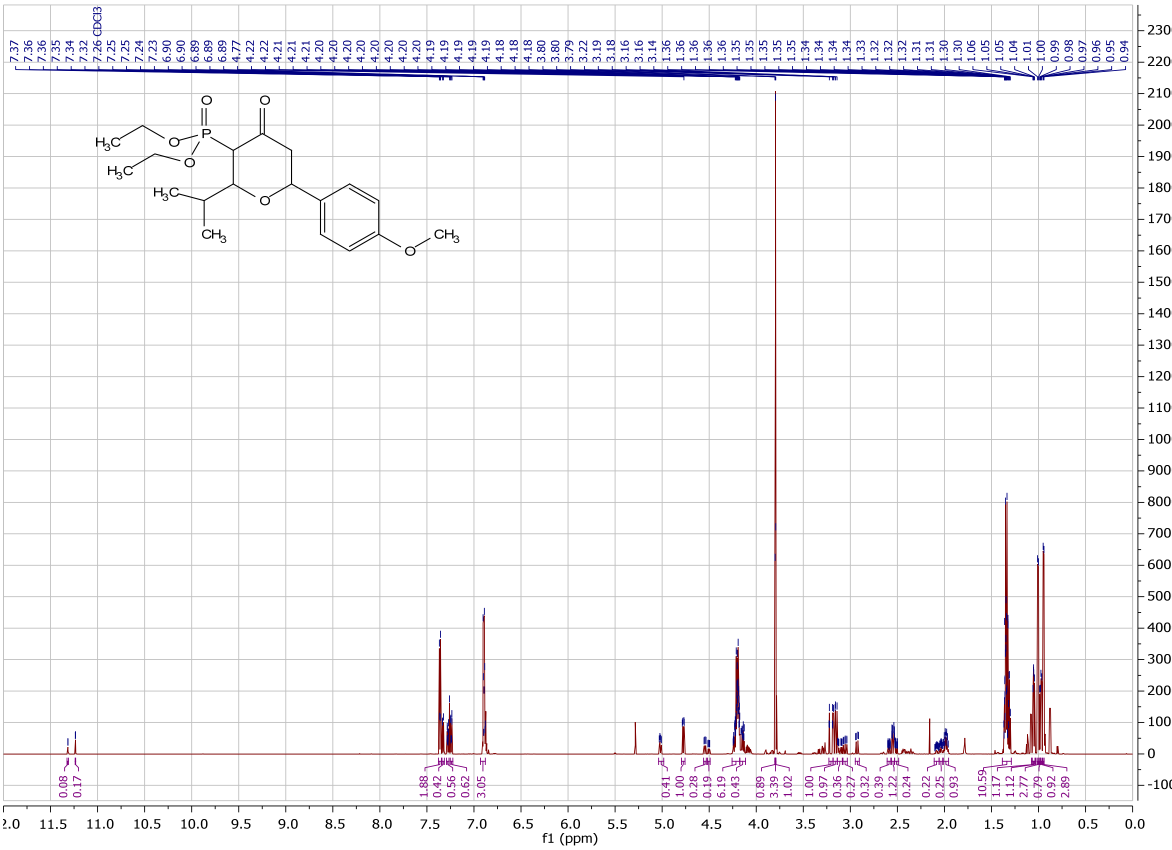

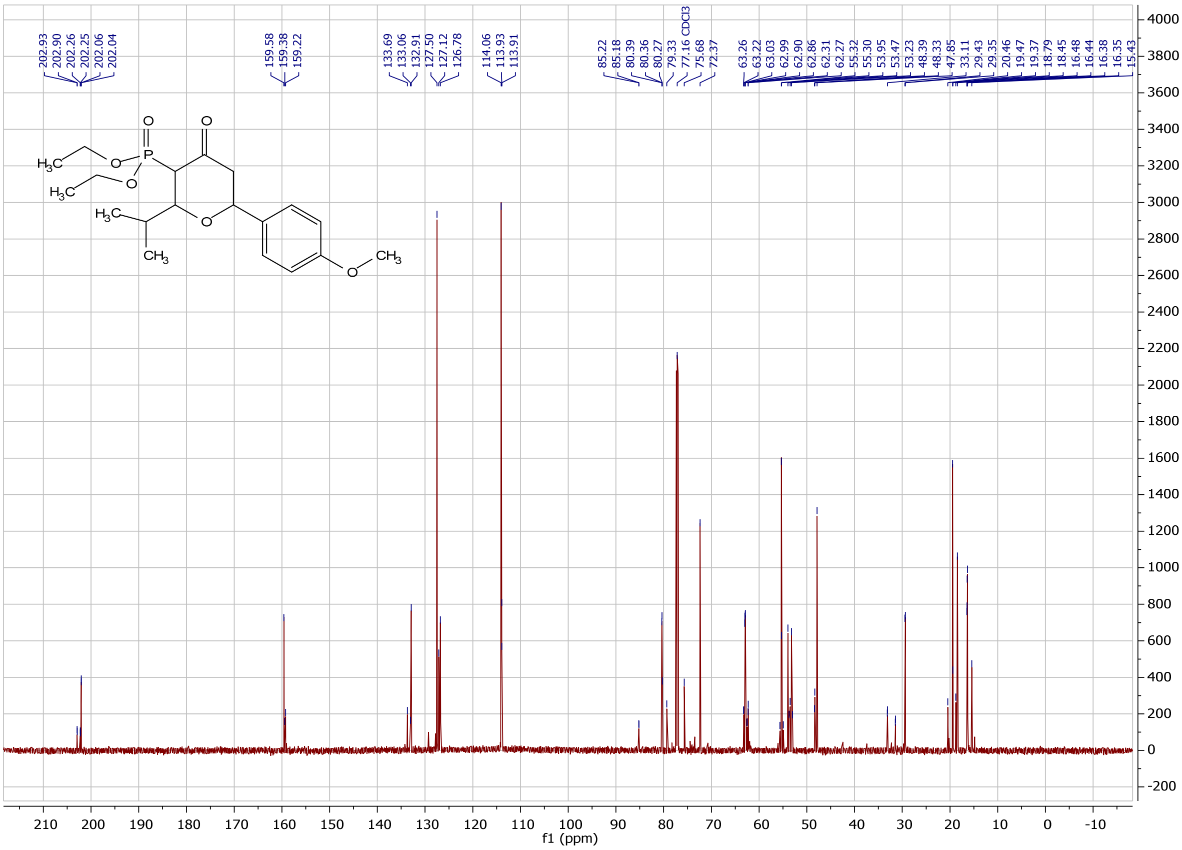

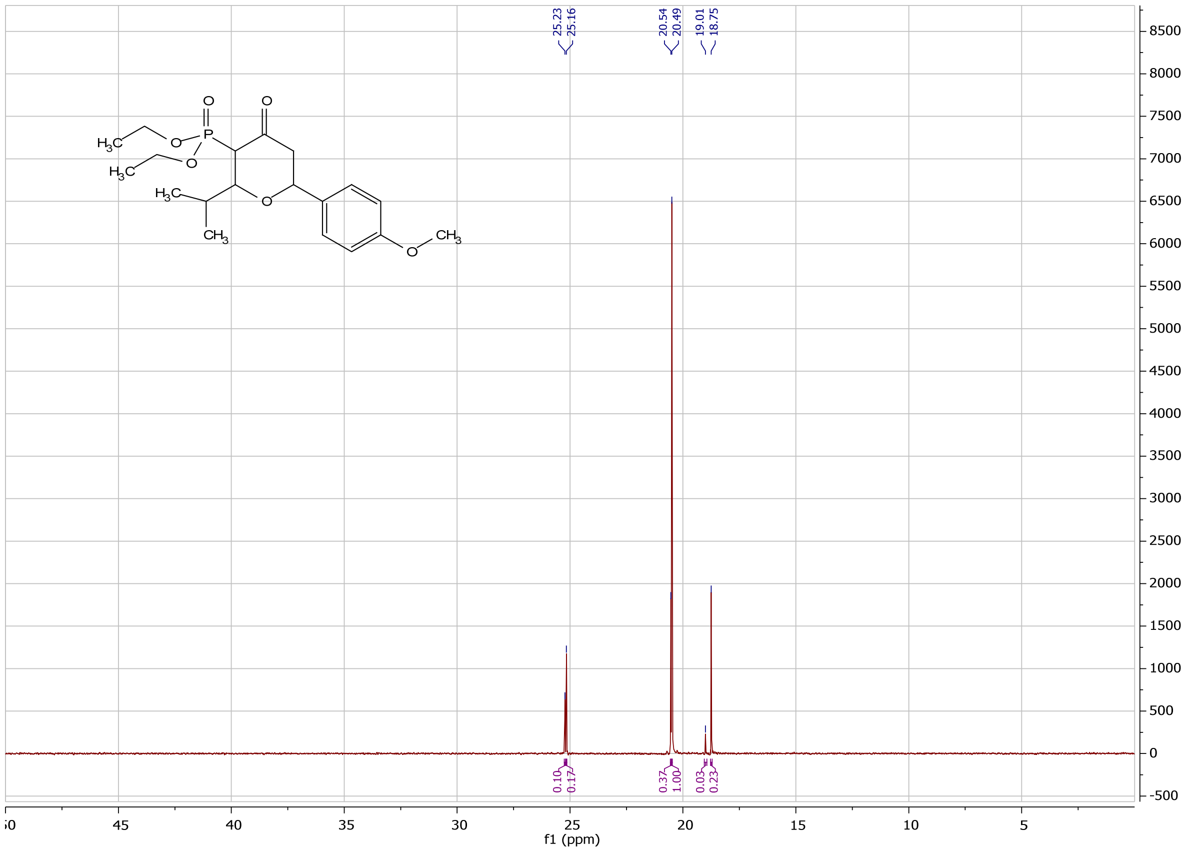

3-Diethoxyphosphoryl-2-isopropyl-6-ferrocenyltetrahydropyran-4-one (**12o**)

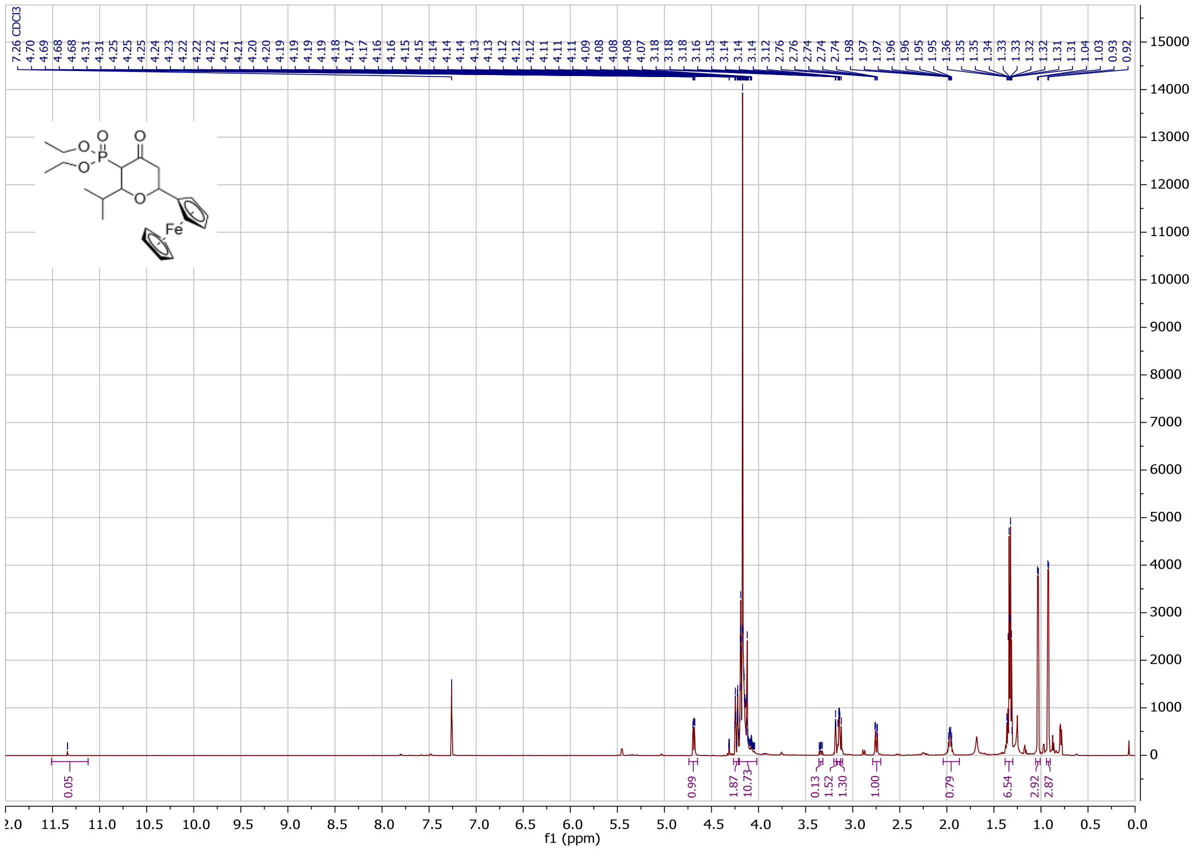

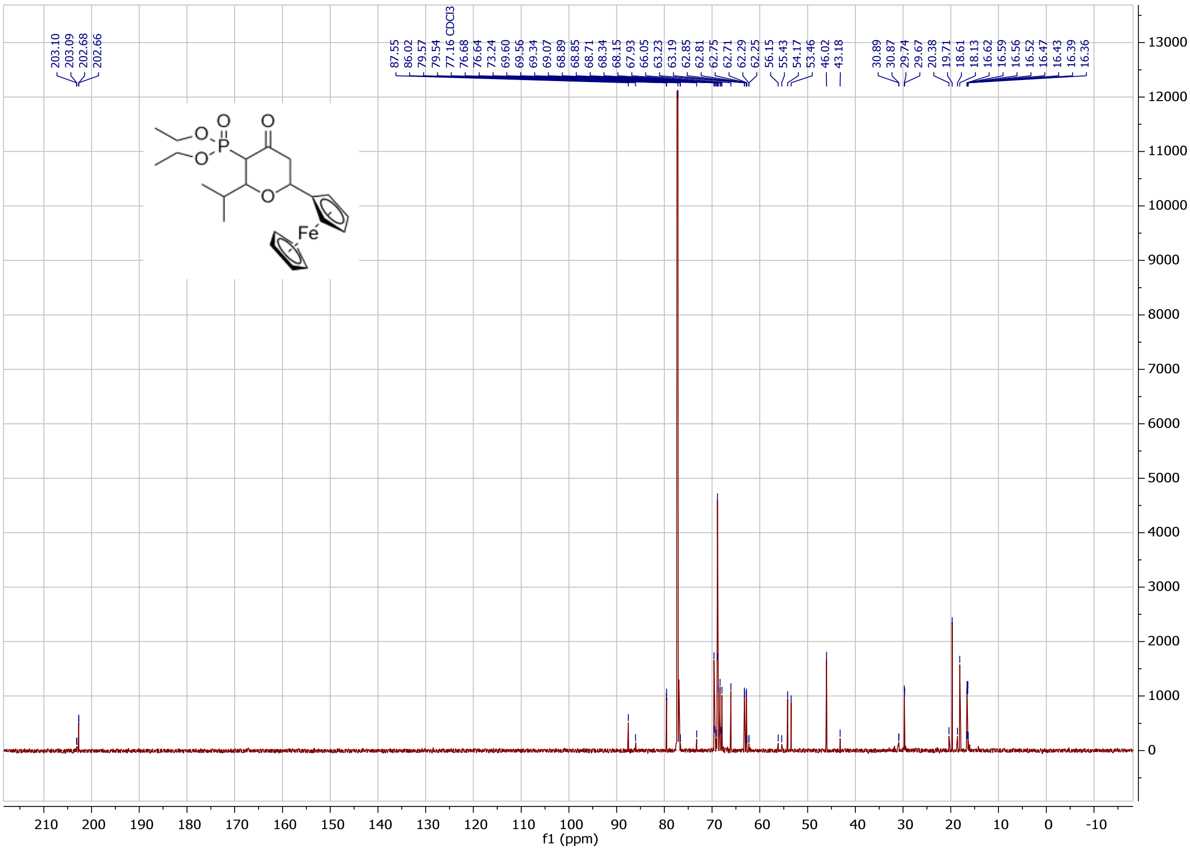

203.10
203.09
202.68
202.66
87.55
86.02
79.57
79.54
77.16 CDCl3
76.68
76.64
73.24
69.60
69.56
69.34
69.07
68.89
68.85
68.71
68.34
68.15
67.93
66.05
63.23
63.19
62.85
62.81
62.75
62.71
62.29
62.25
56.15
55.43
54.17
53.46
46.02
43.18
30.89
30.87
29.74
29.67
20.38
19.71
18.61
18.13
16.62
16.59
16.56
16.52
16.47
16.43
16.39
16.36
Fe
f1 (ppm)

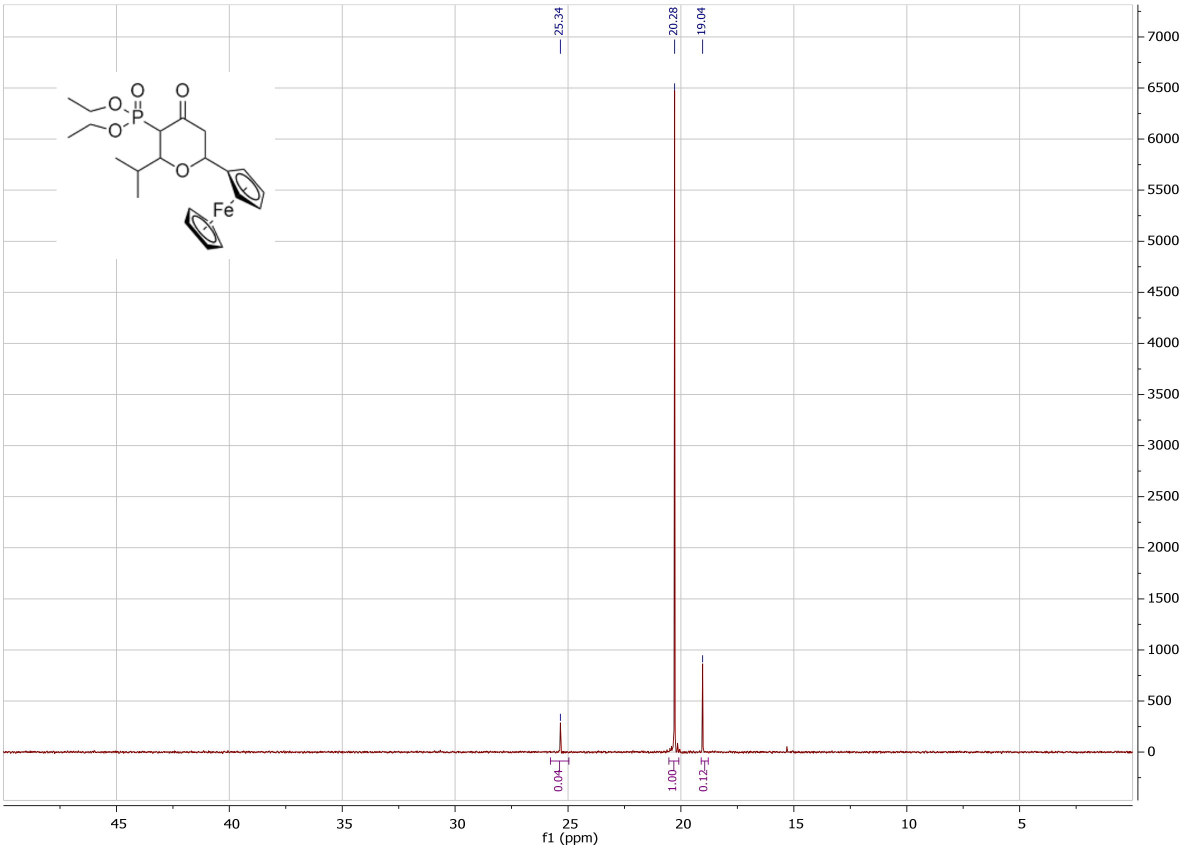

25.34
20.28
19.04
0.04
1.00
0.12
Fe
f1 (ppm)

5-Diethoxyphosphoryl-2-ethyltetrahydropyran-4-one (**14a**)

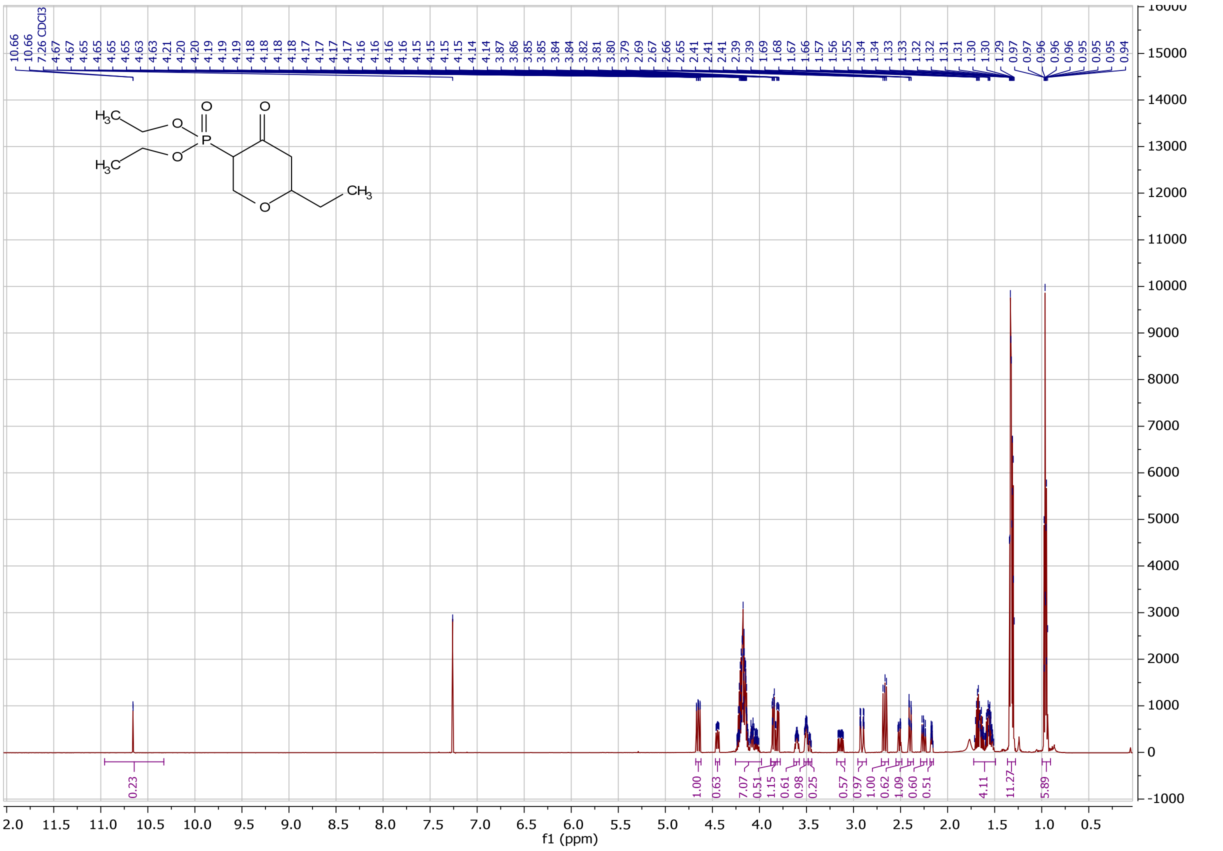

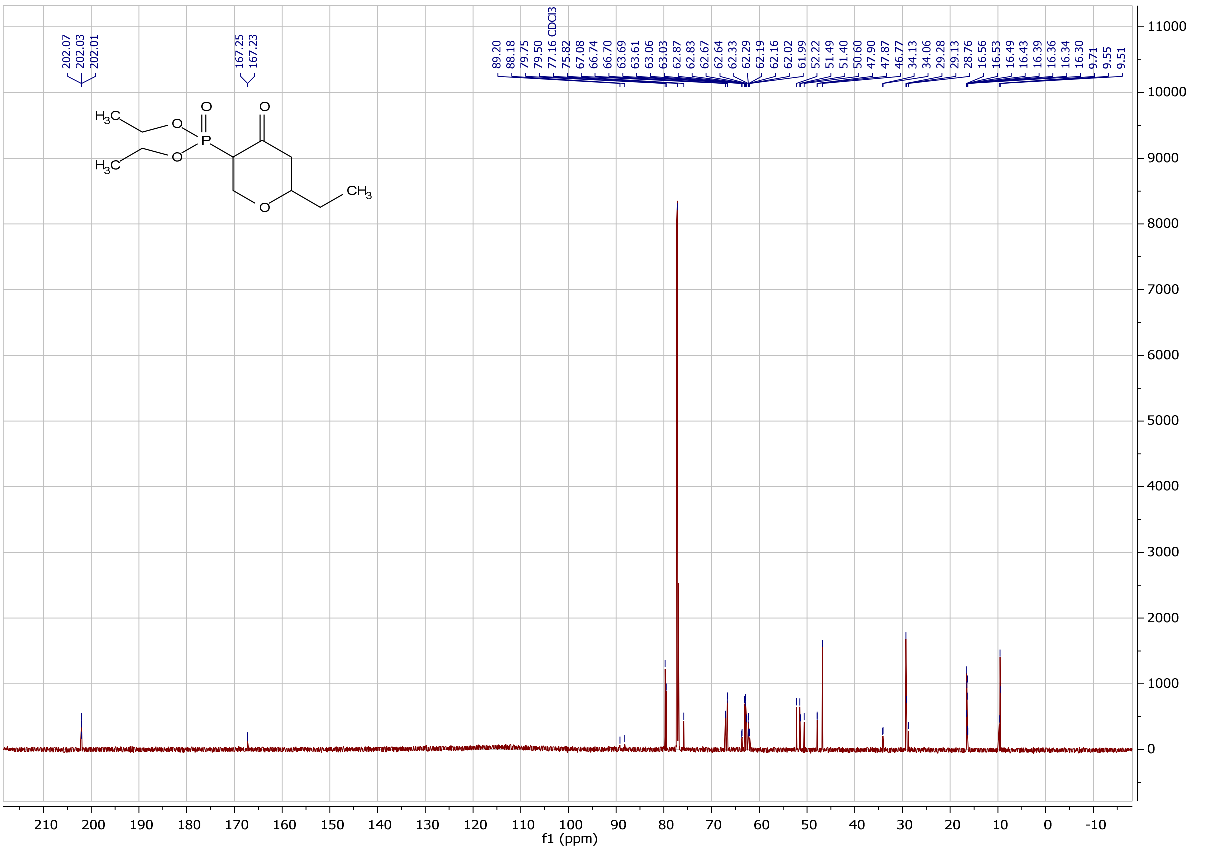

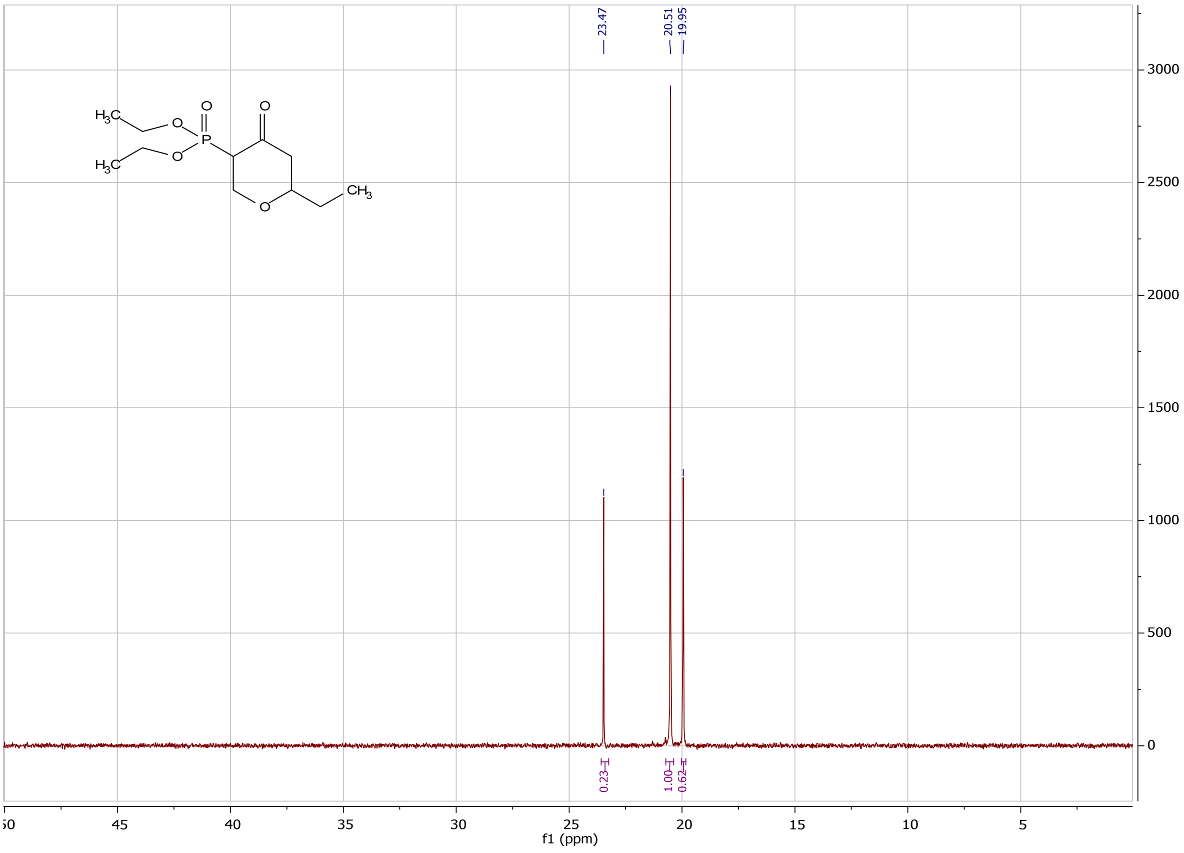

5-Diethoxyphosphoryl-2-isopropyltetrahydropyran-4-one (**14b**)

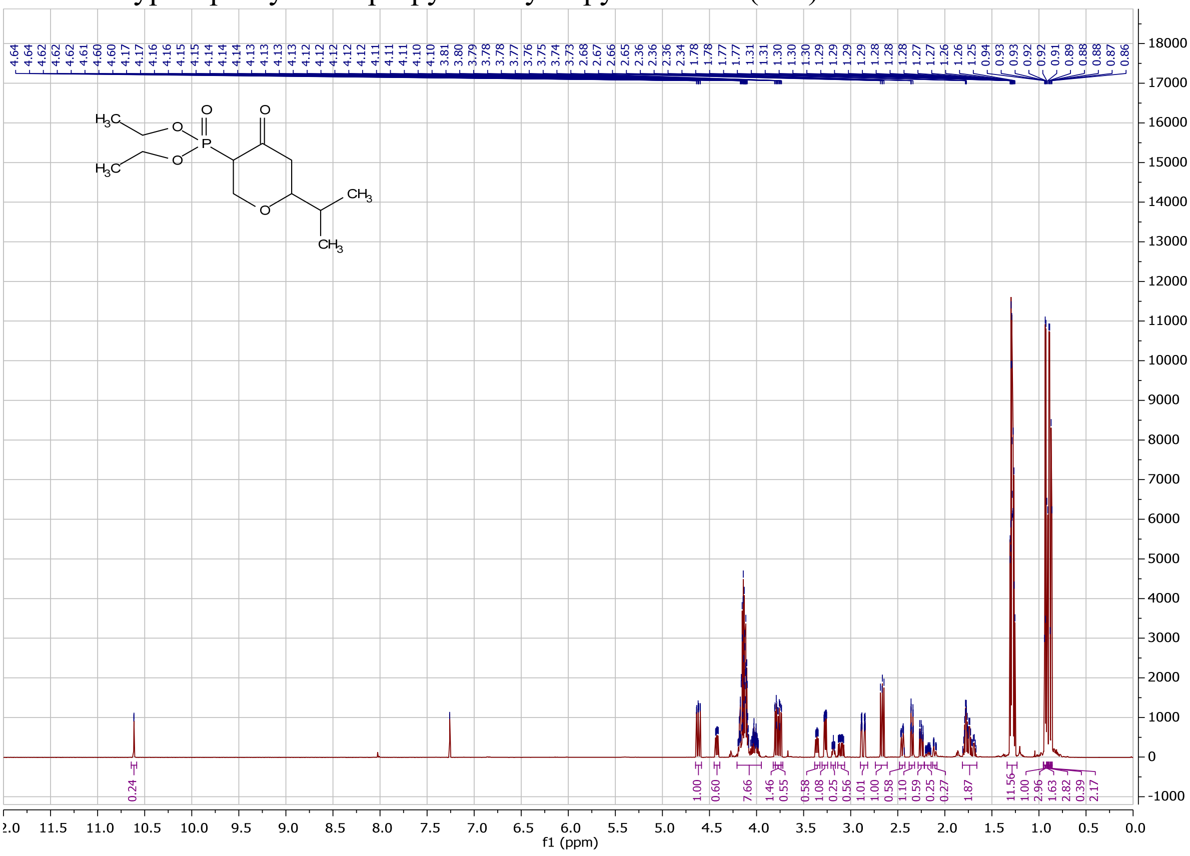

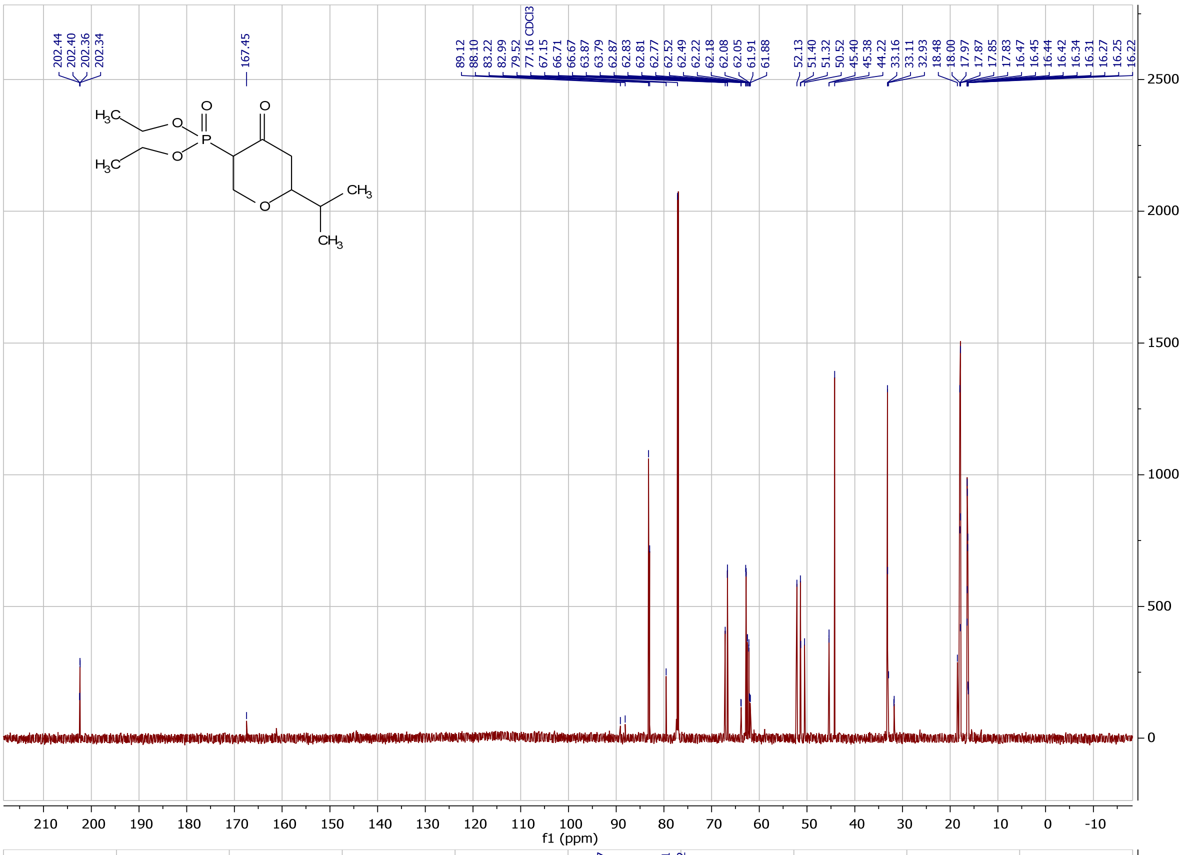
202.44
202.40
202.36
202.34
167.45
89.12
88.10
83.22
82.99
79.52
77.16 CDCl3
67.15
66.71
66.67
63.87
63.79
62.87
62.83
62.81
62.77
62.52
62.49
62.22
62.18
62.08
62.05
61.91
61.88
52.13
51.40
51.32
50.52
45.40
45.38
44.22
33.16
33.11
32.93
18.48
18.00
17.97
17.87
17.85
17.83
16.47
16.45
16.44
16.42
16.34
16.31
16.27
16.25
16.22
f1 (ppm)

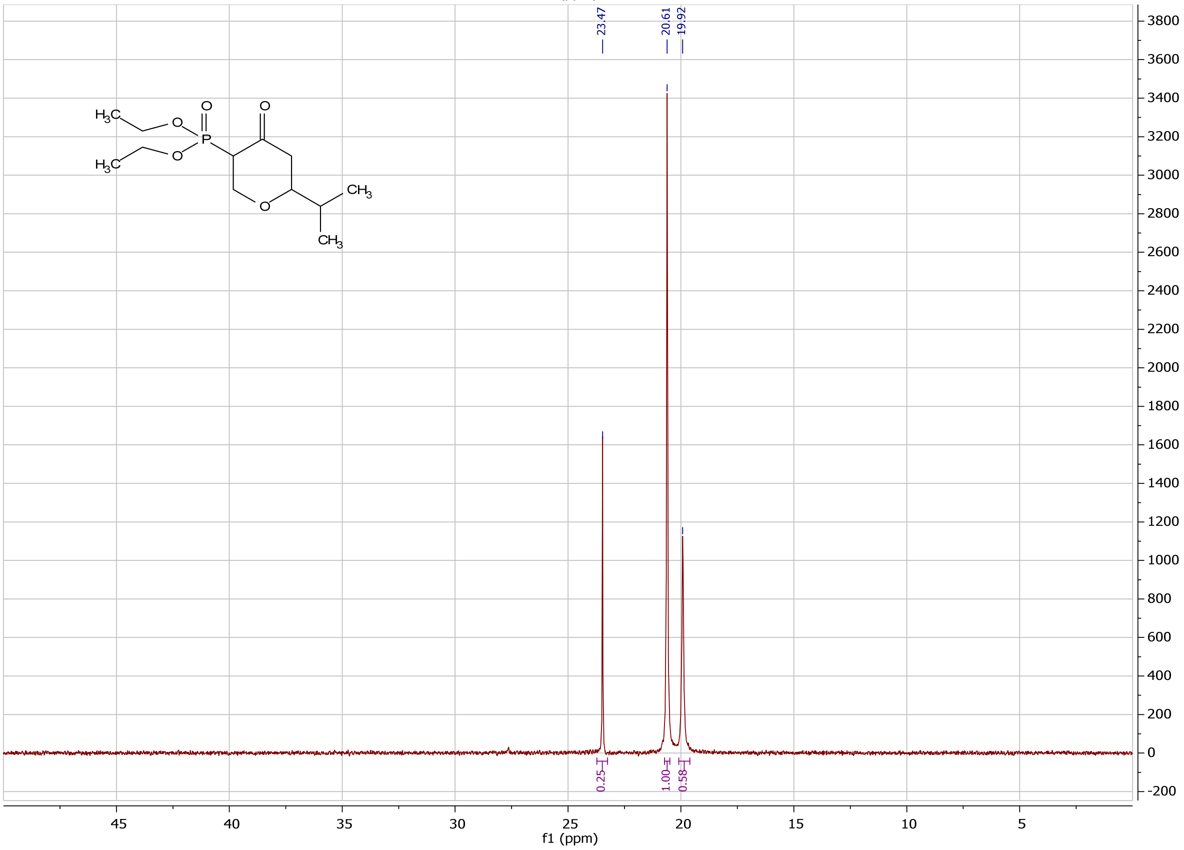
23.47
20.61
19.92
0.25
1.00
0.58
f1 (ppm)

5-Diethoxyphosphoryl-2-phenyltetrahydropyran-4-one (**14c**)

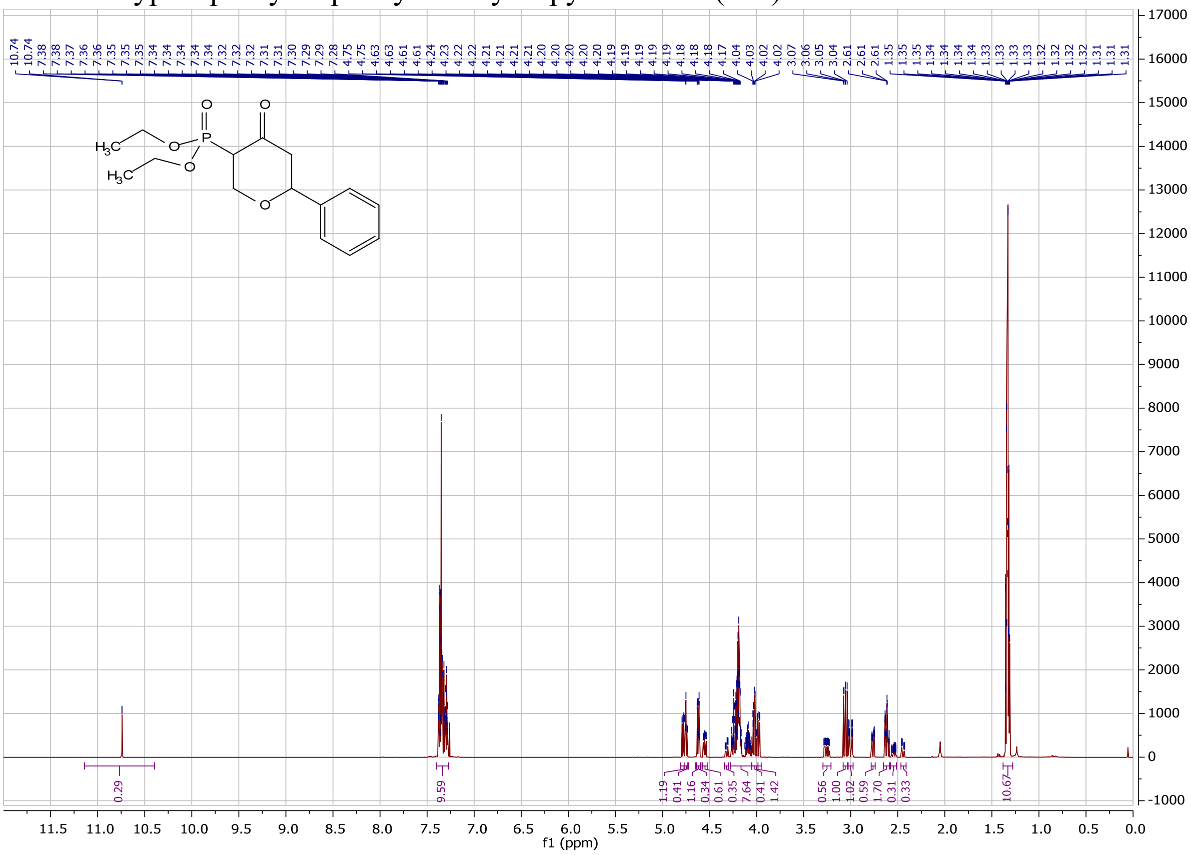

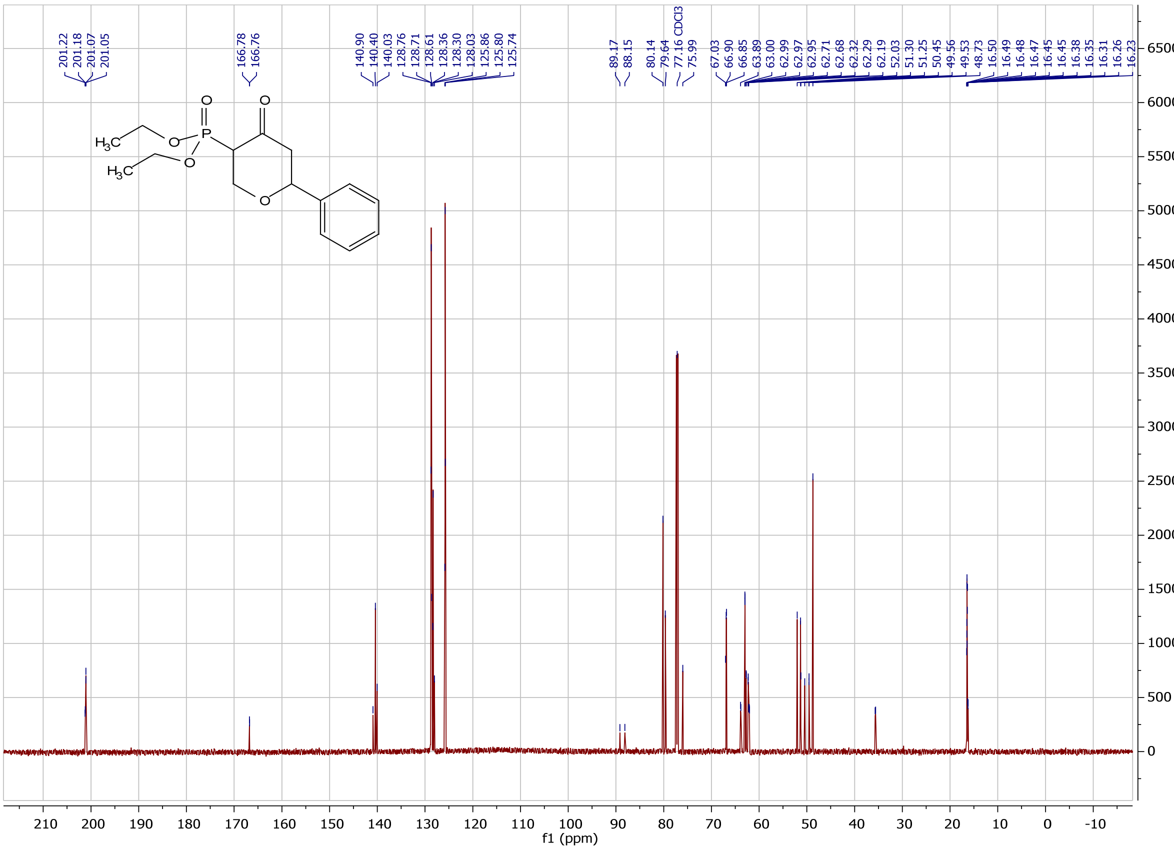

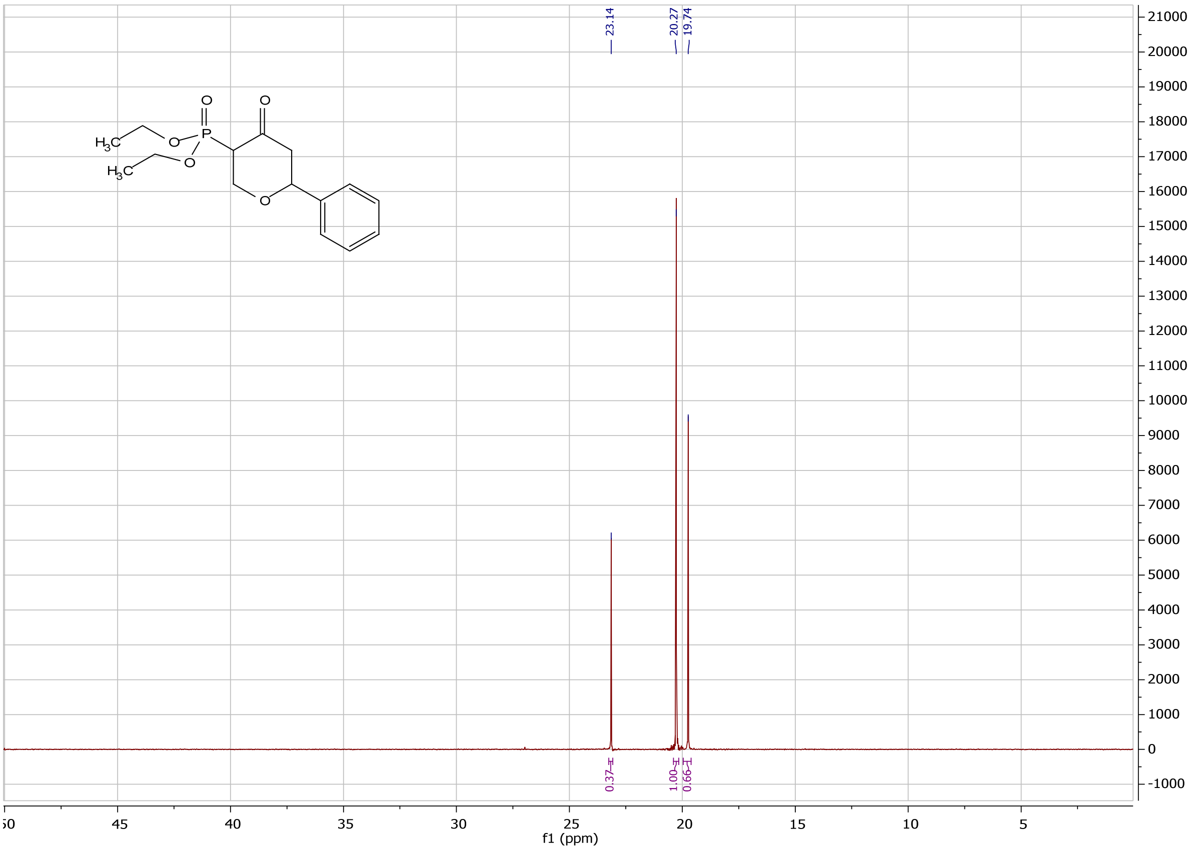

5-Diethoxyphosphoryl-2-(4-methoxyphenyl)tetrahydropyran-4-one (**14d**)

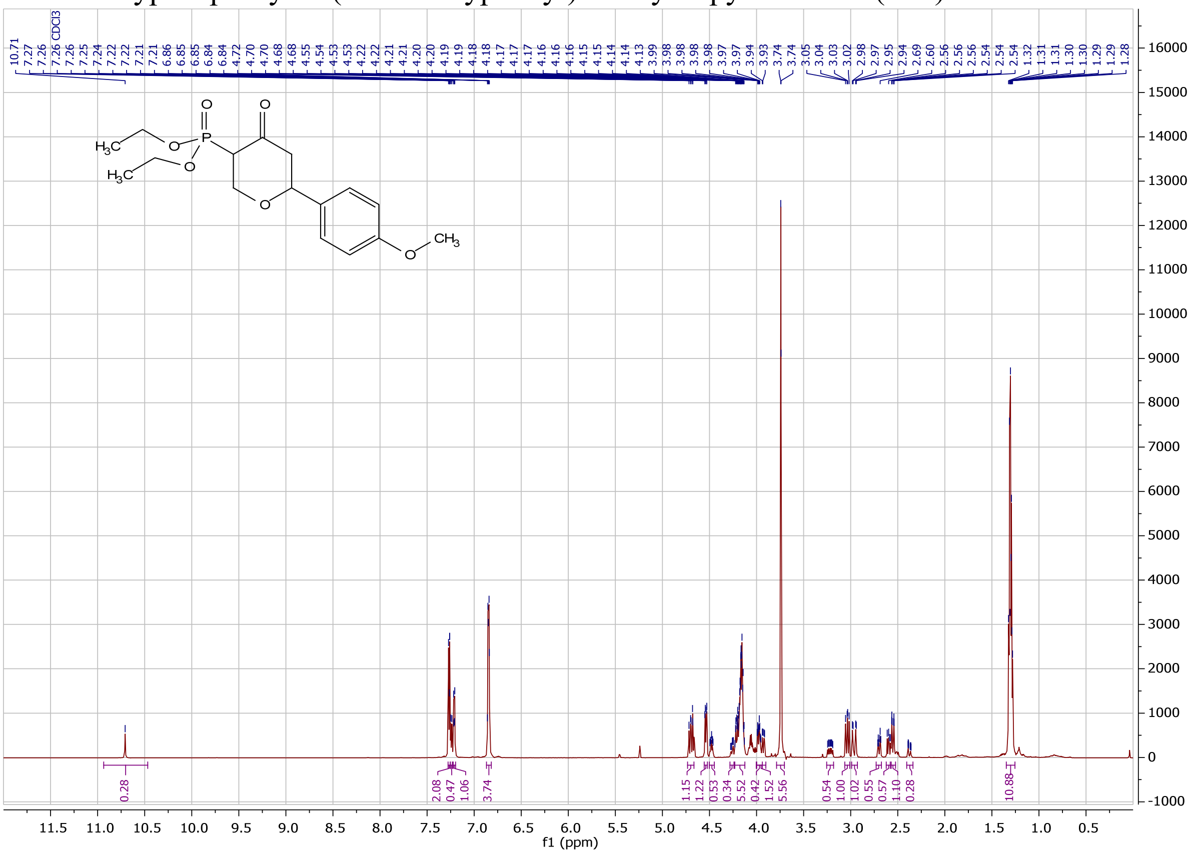

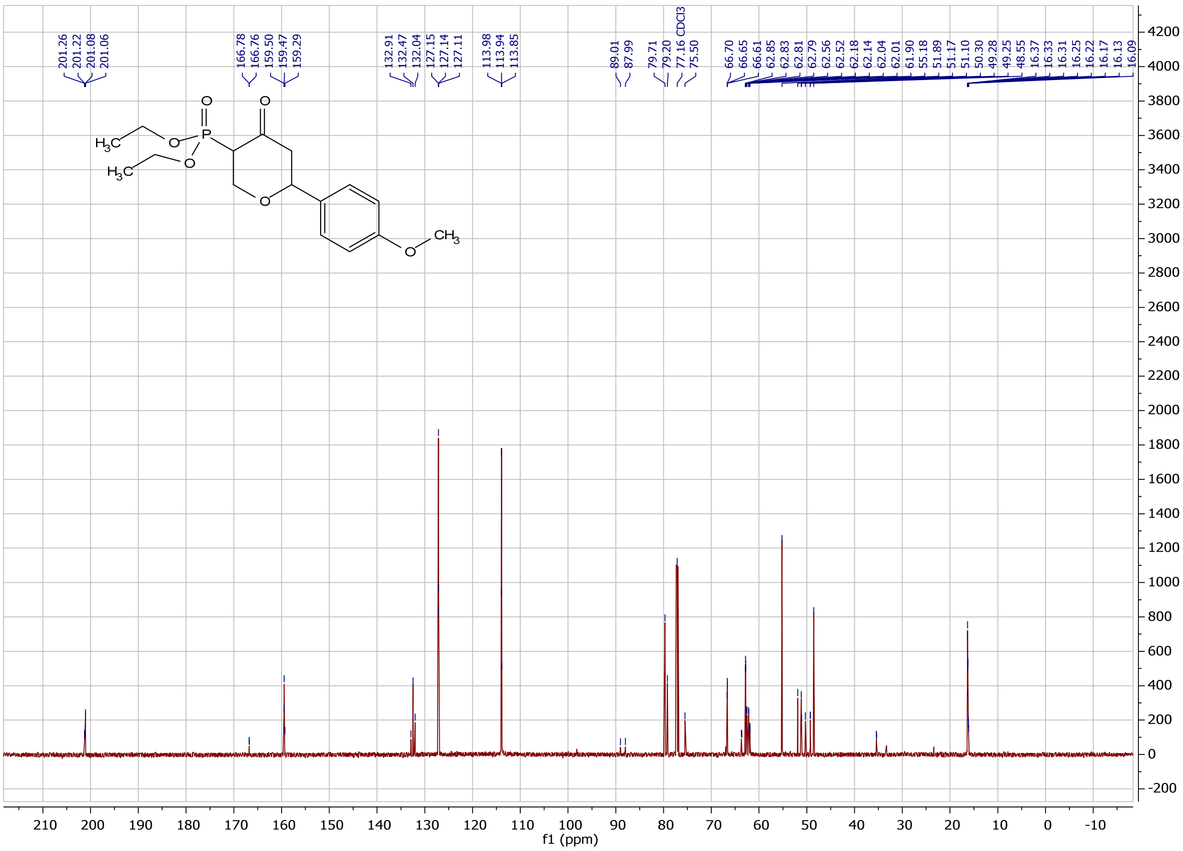

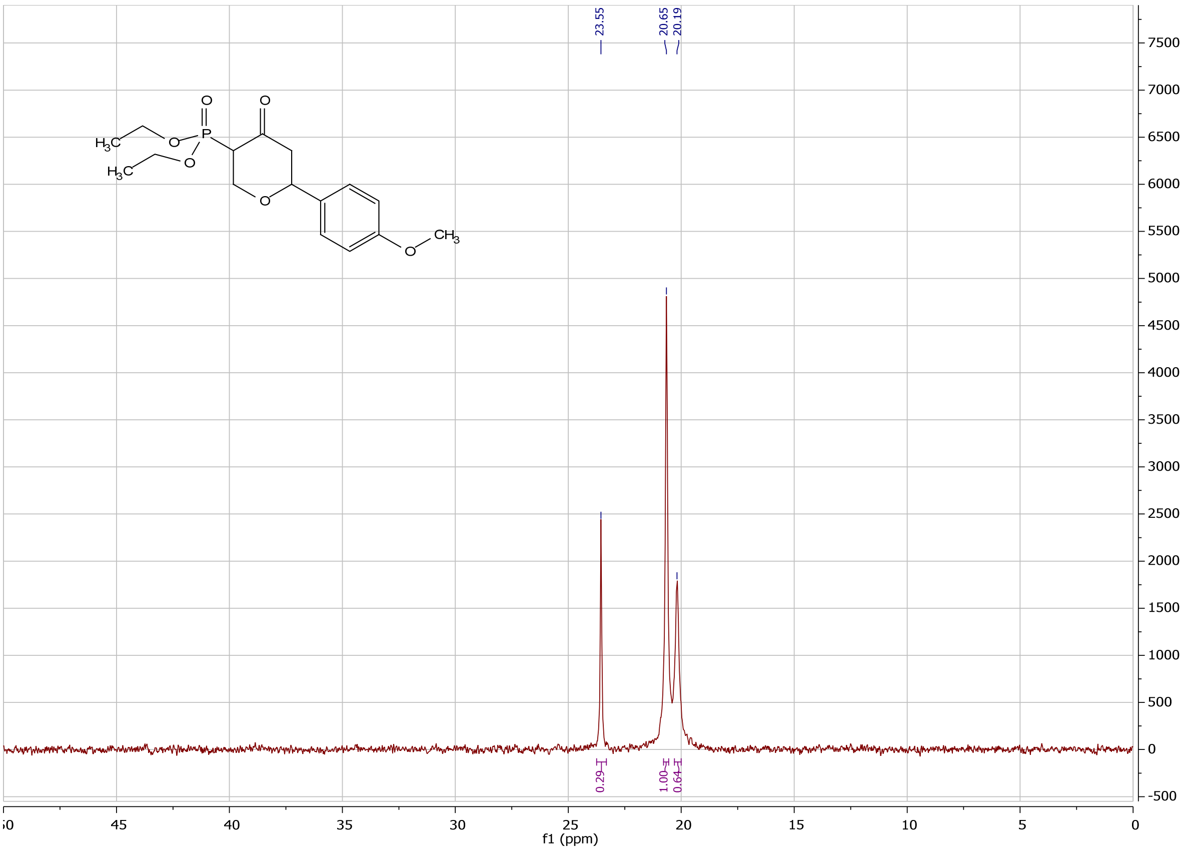

6-Ethyl-2-isopropyl-3-methylidenetetrahydro-4H-pyran-4-one (**13a**)

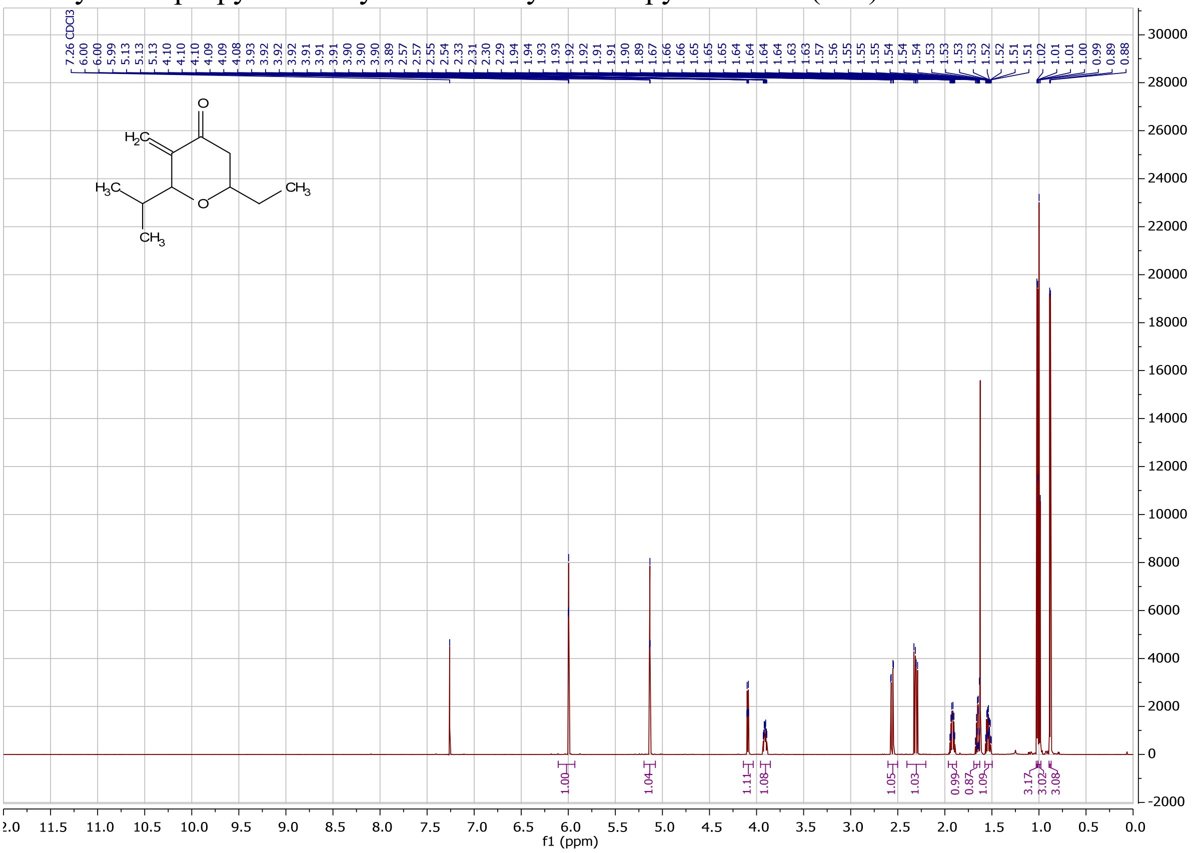

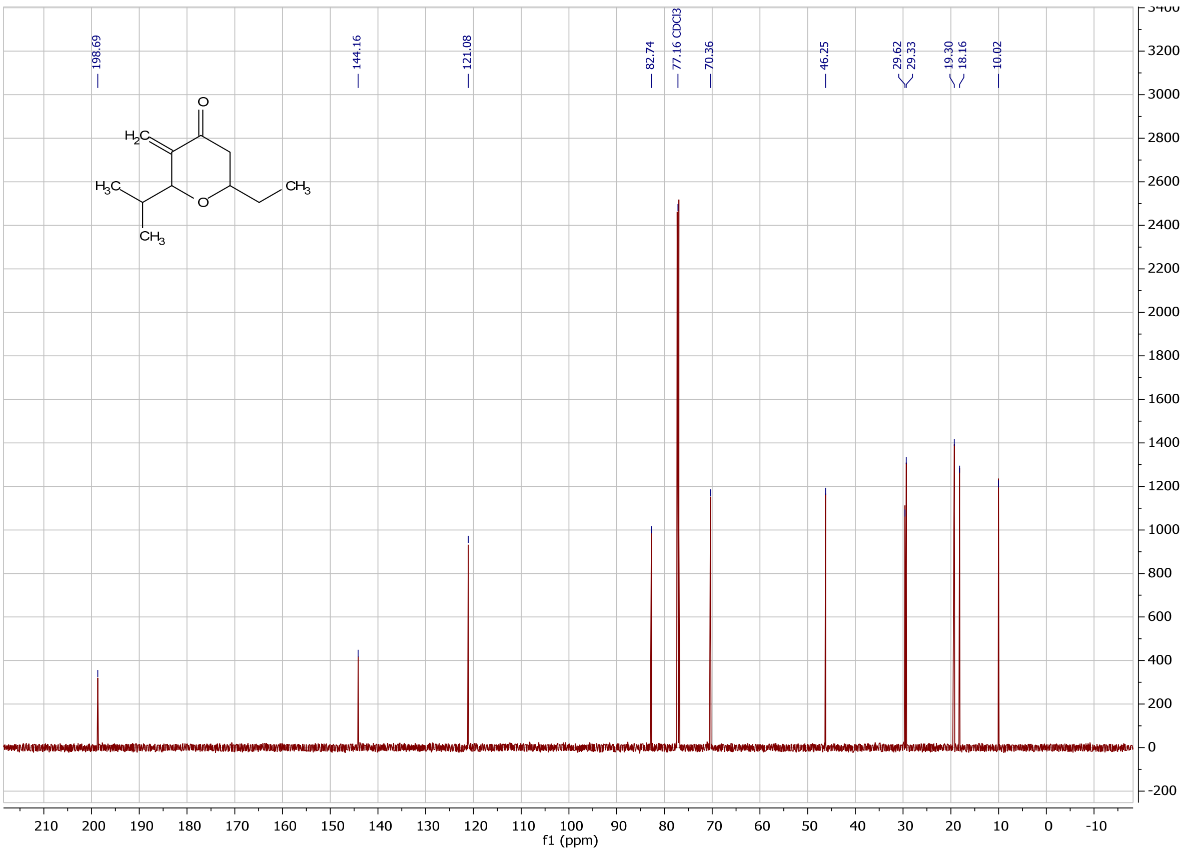

2-Buthyl-6-ethyl-3-methylidenetetrahydro-4H-pyran-4-one (**13b**)

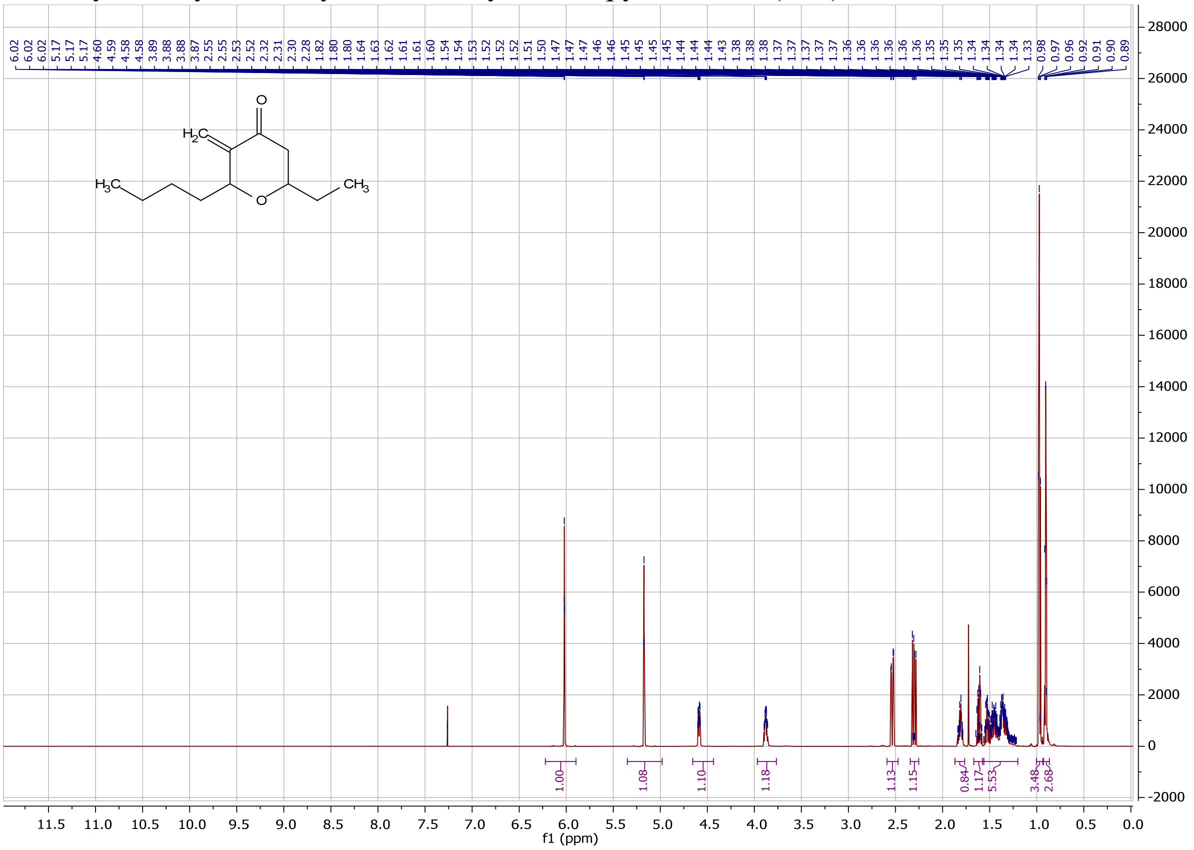

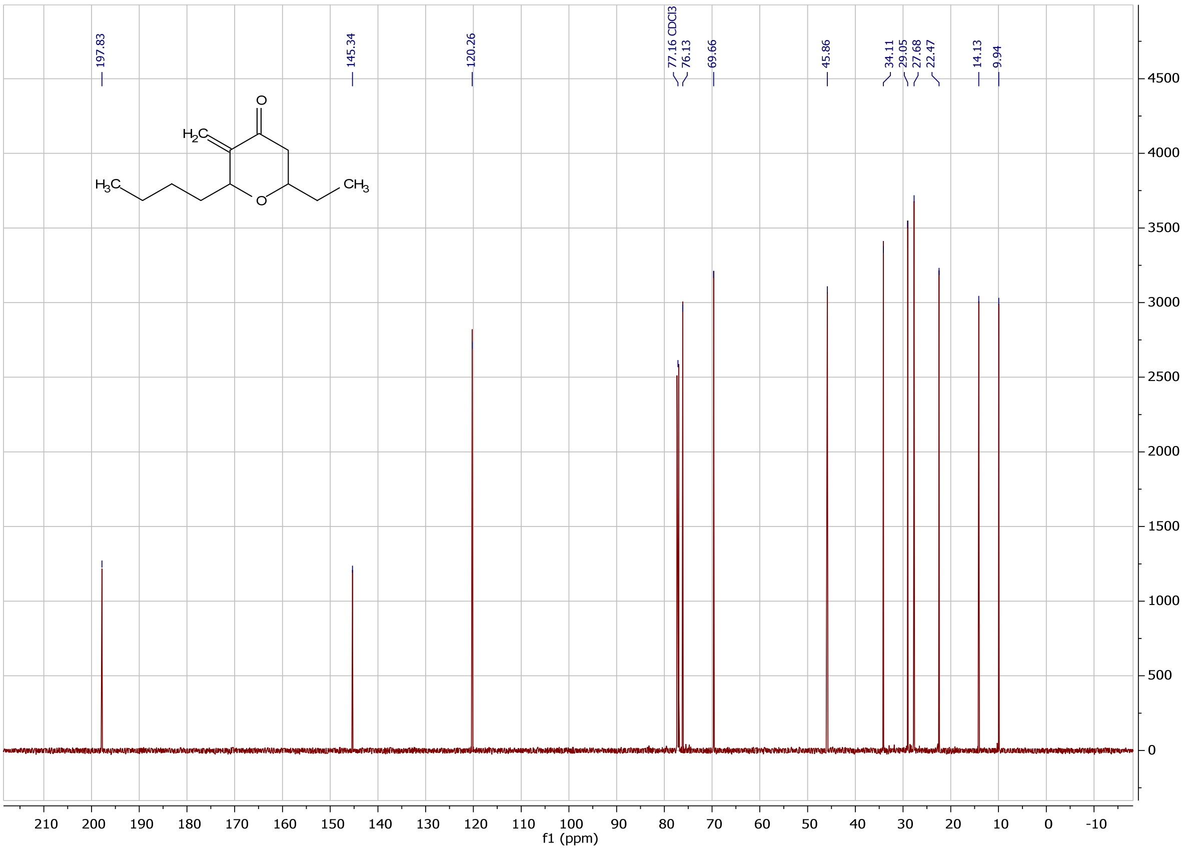

6-Ethyl -3-methylidene-2-phenyltetrahydro-4H-pyran-4-one (**13c**)

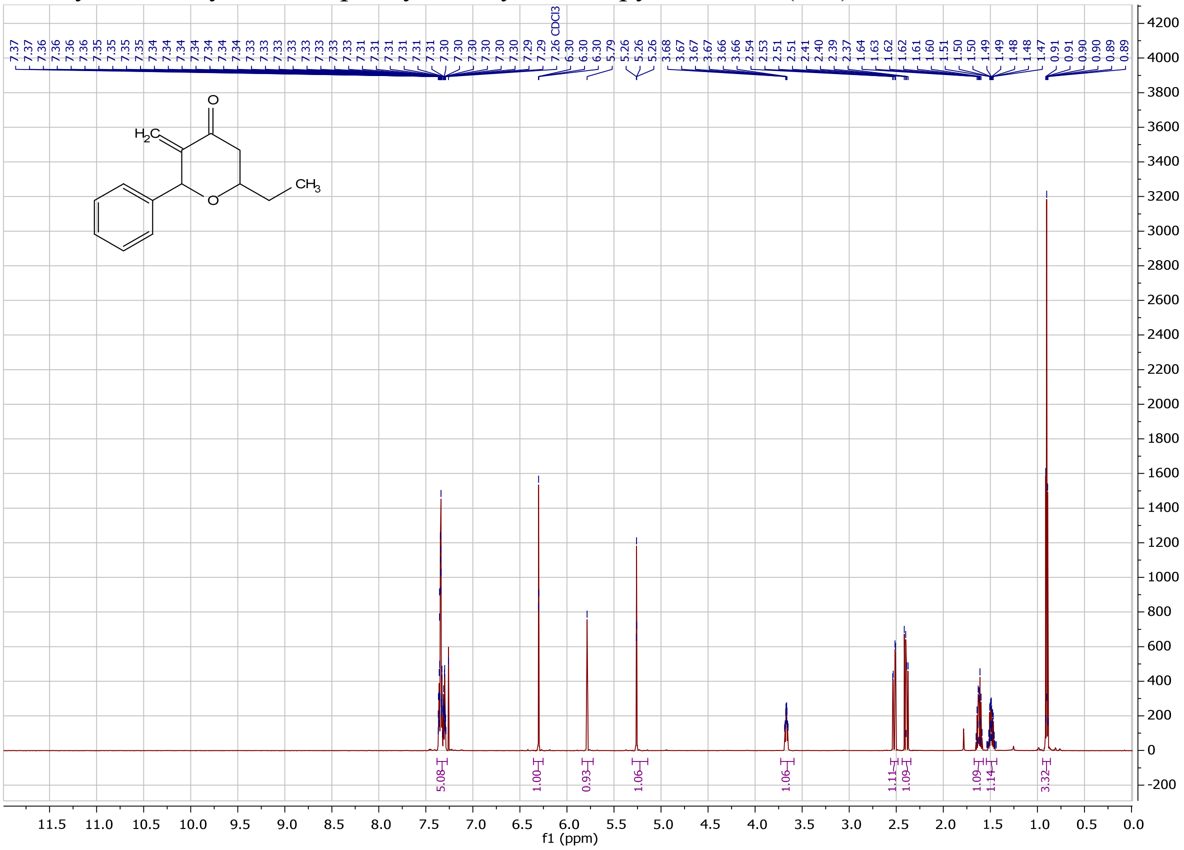

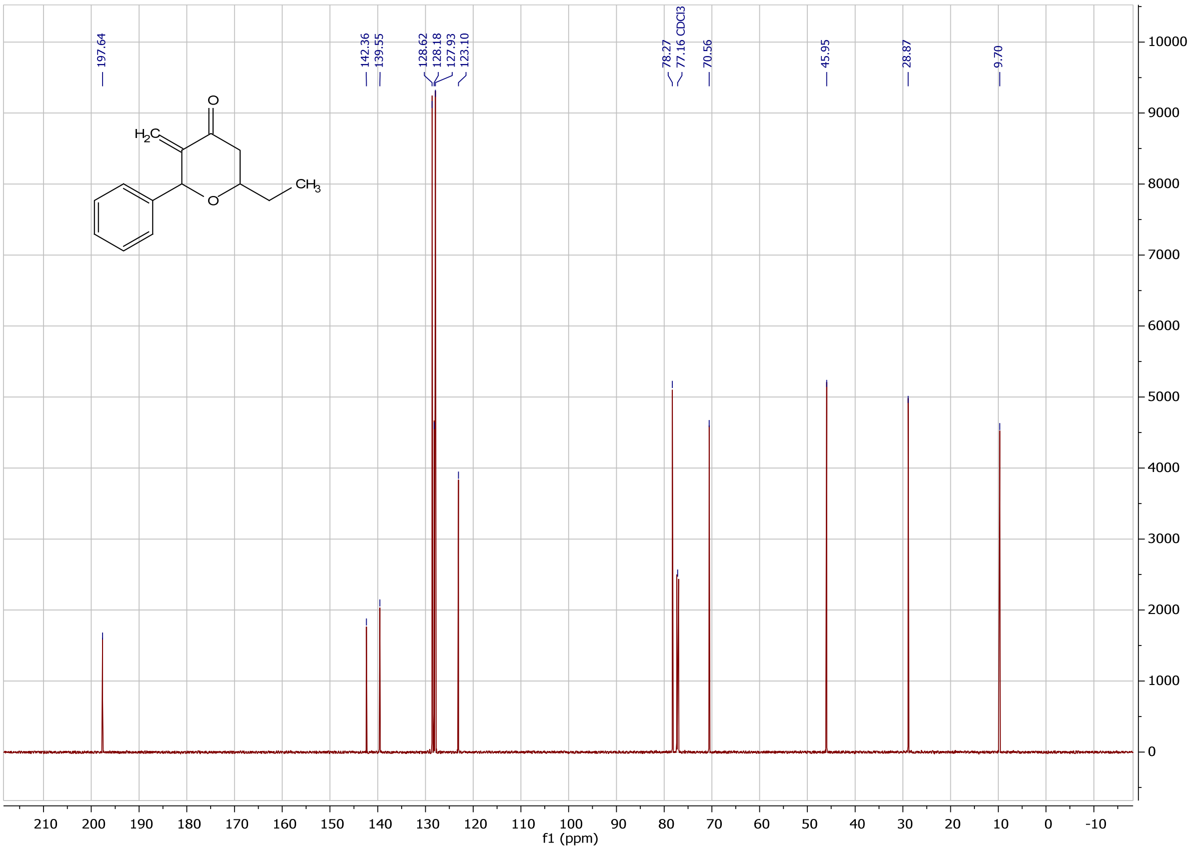

2,6-Diisopropyl-3-methylidenetetrahydro-4H-pyran-4-one (**13d**)

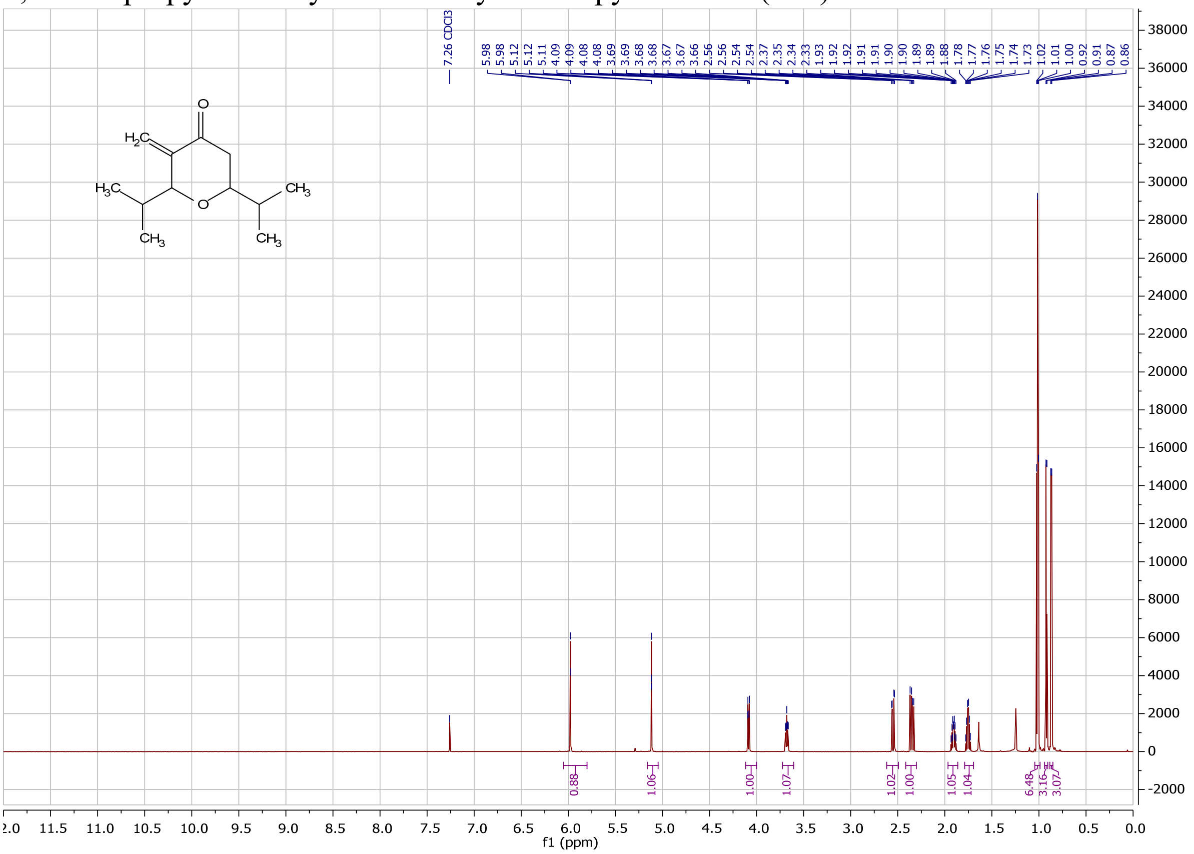

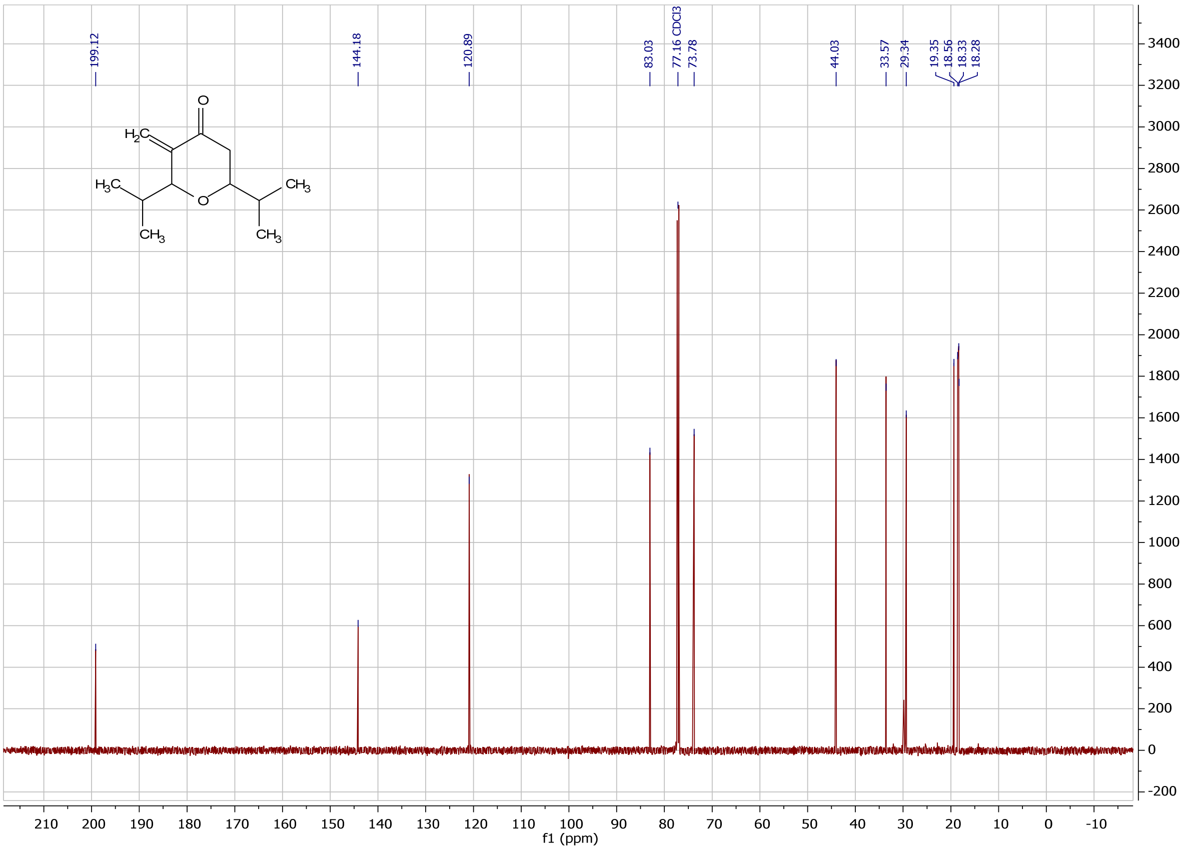

2-Buthyl-6-isopropyl-3-methylidenetetrahydro-4H-pyran-4-one (**13e**)

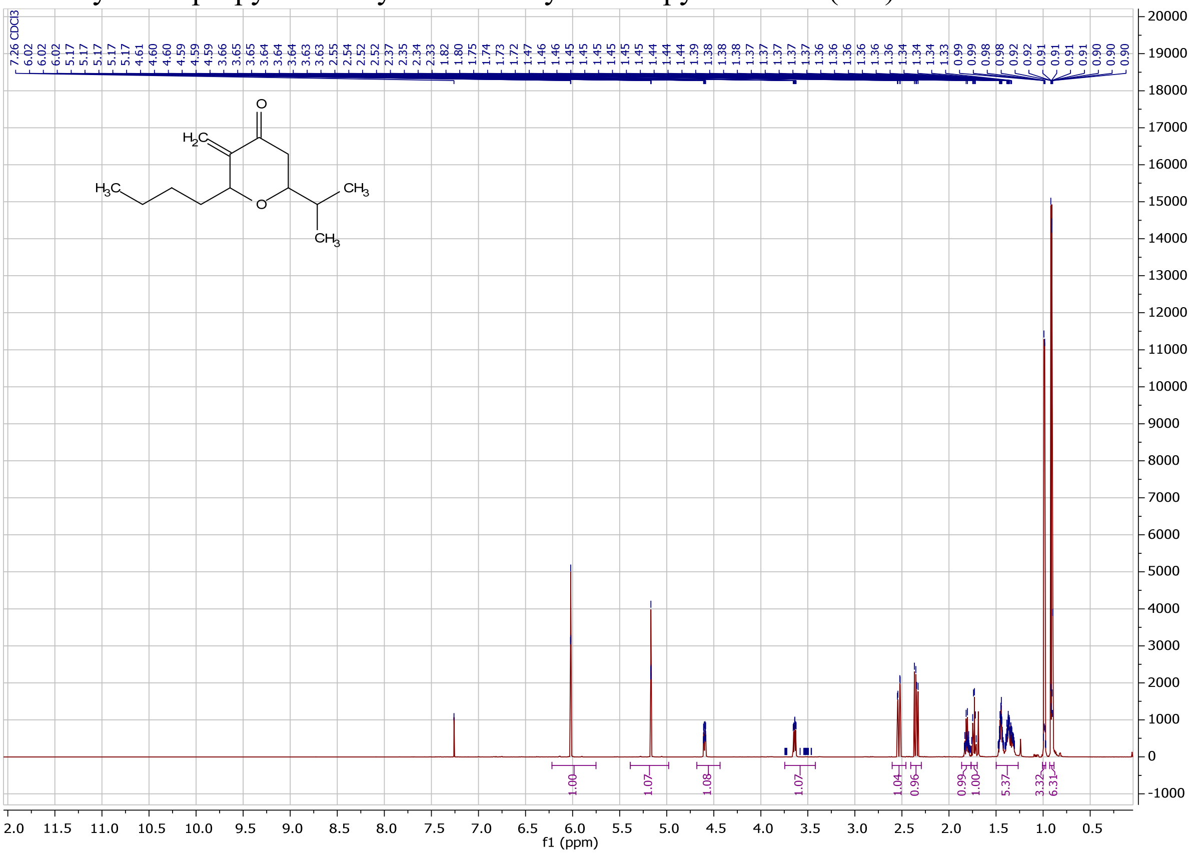

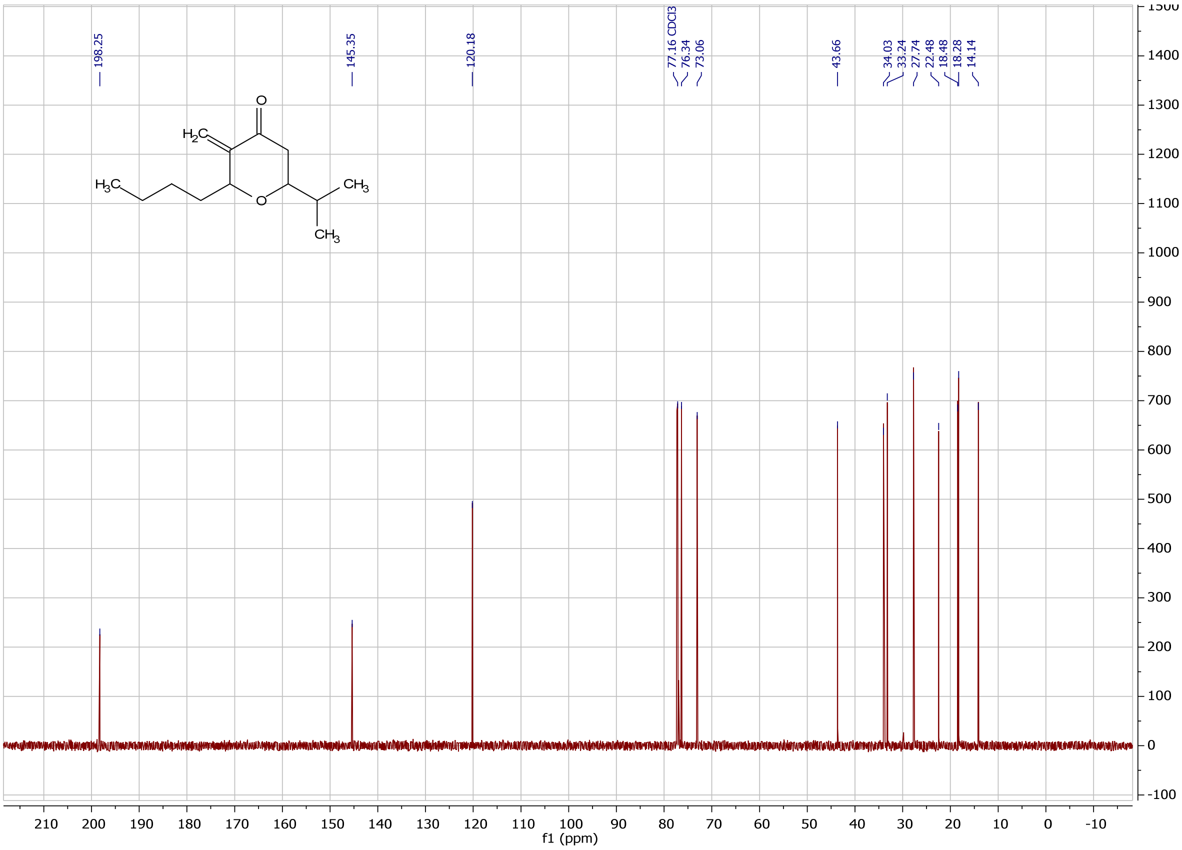

6-Isopropyl-3-methylidene-2-phenyltetrahydro-4H-pyran-4-one (**13f**)

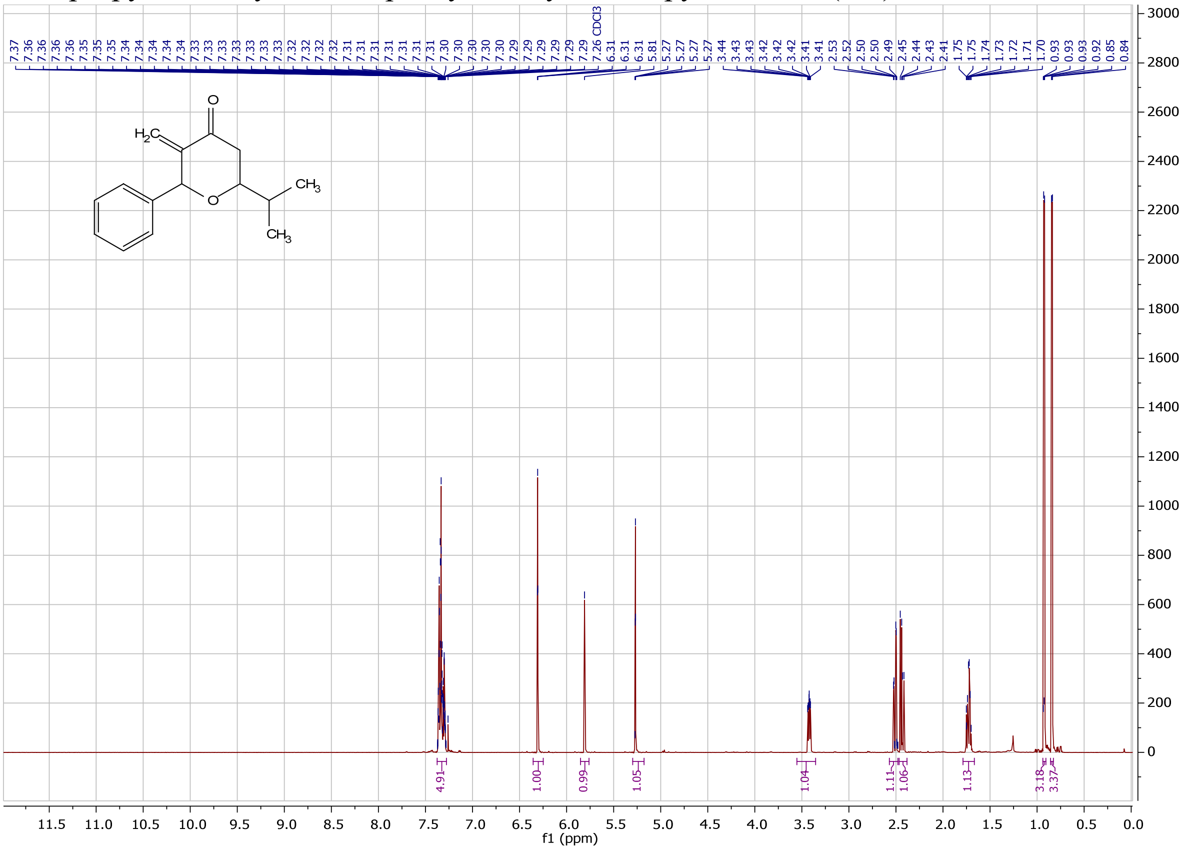

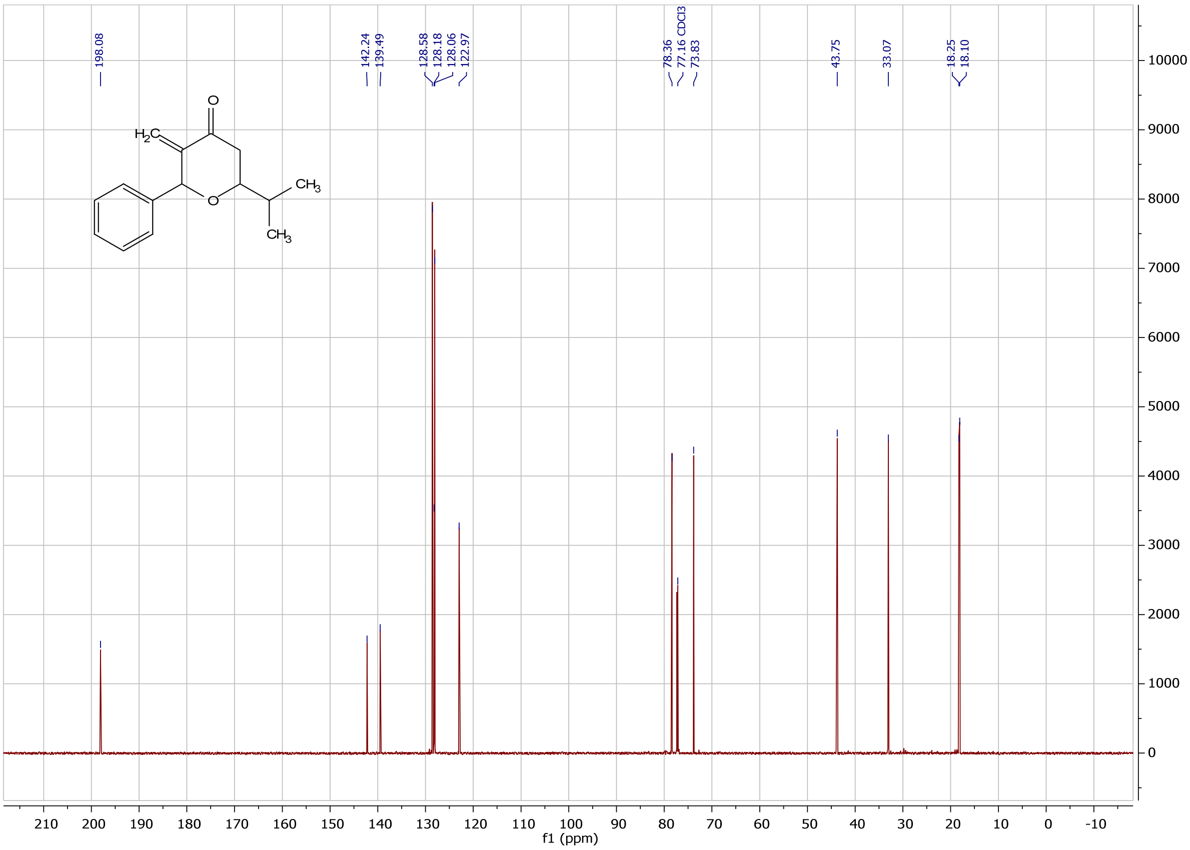

2-Isopropyl-3-methylidene-6-phenyltetrahydro-4H-pyran-4-one (**13g**)

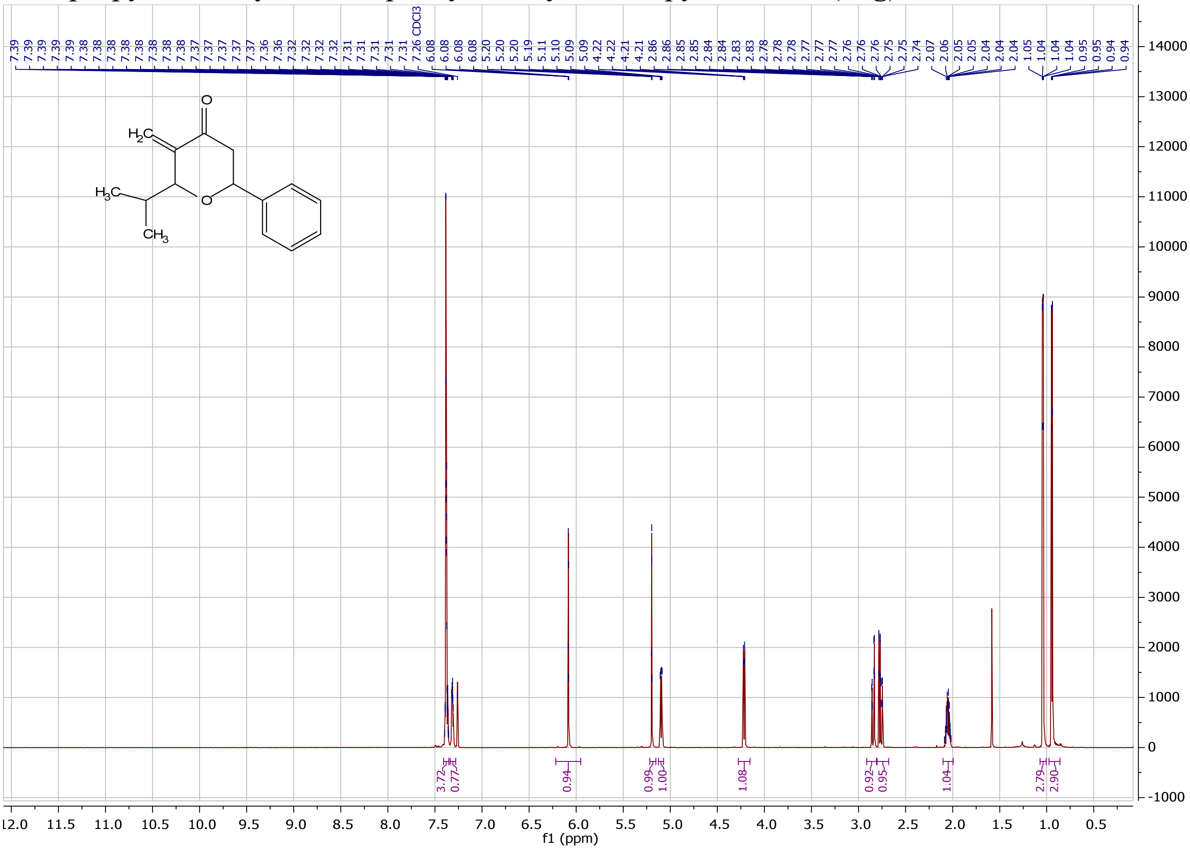

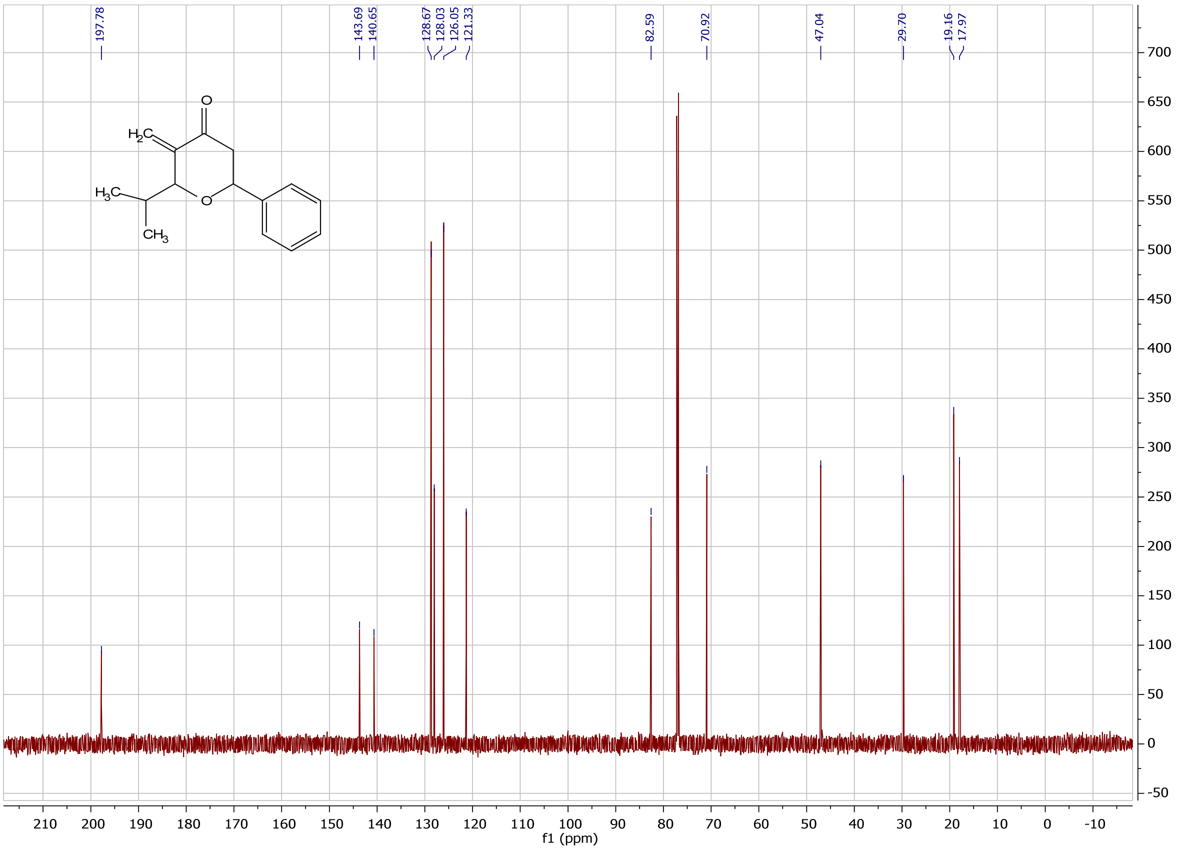

2-Buthyl-3-methylidene-6-phenyltetrahydro-4H-pyran-4-one (**13h**)

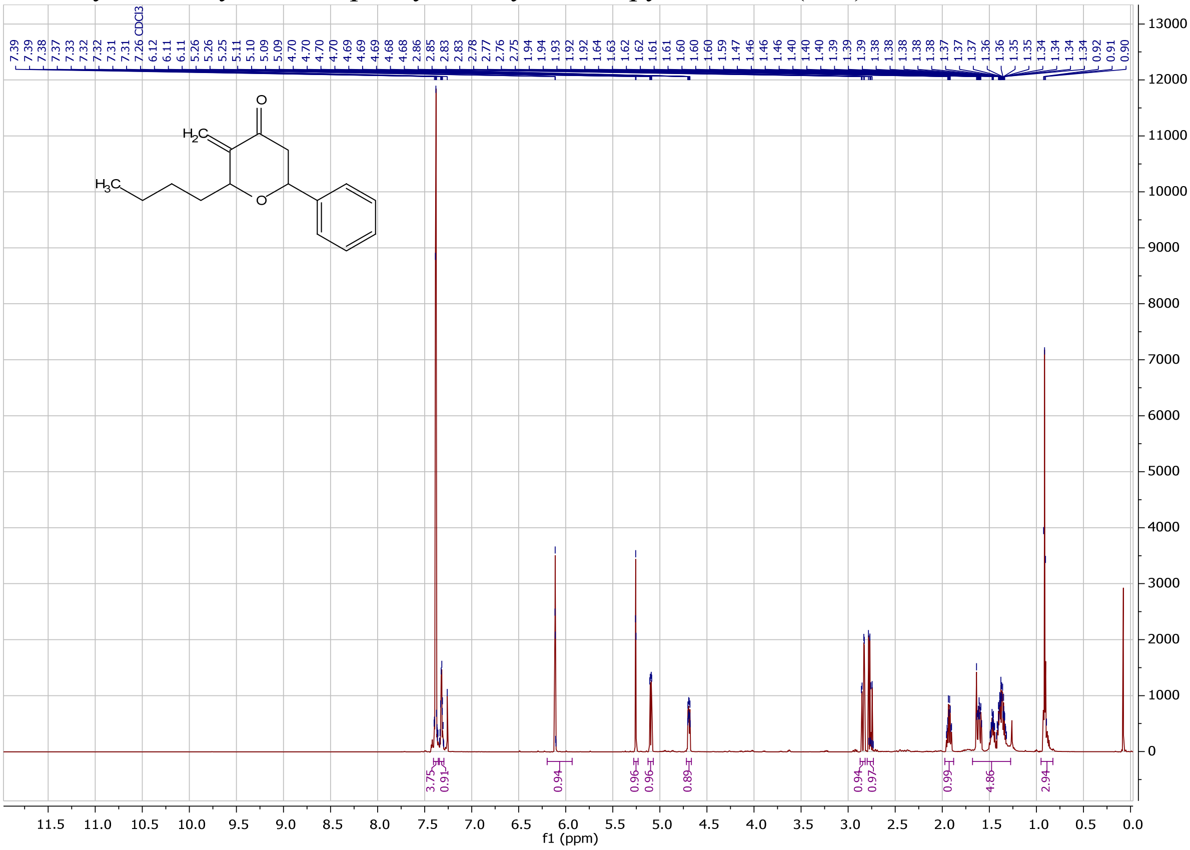

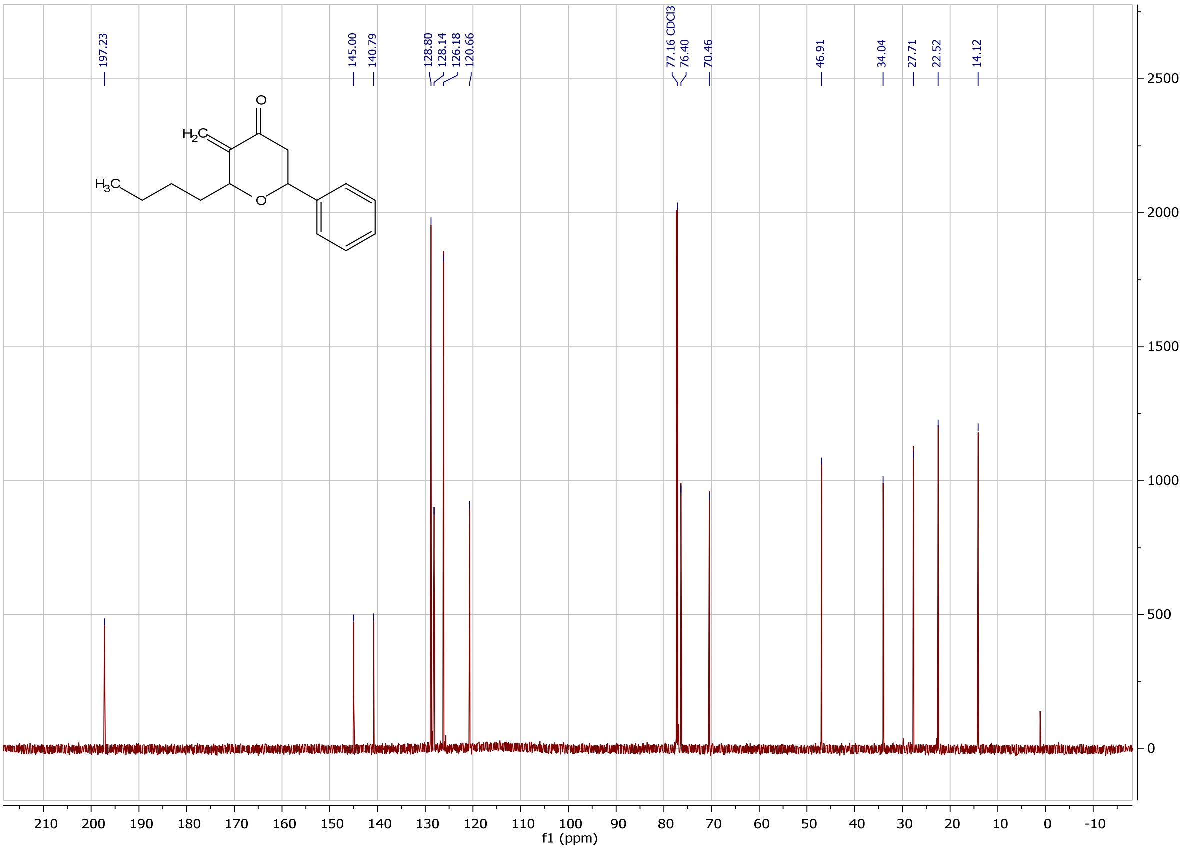

3-Methylidene-2,6-diphenyltetrahydro-4H-pyran-4-one (**13i**)

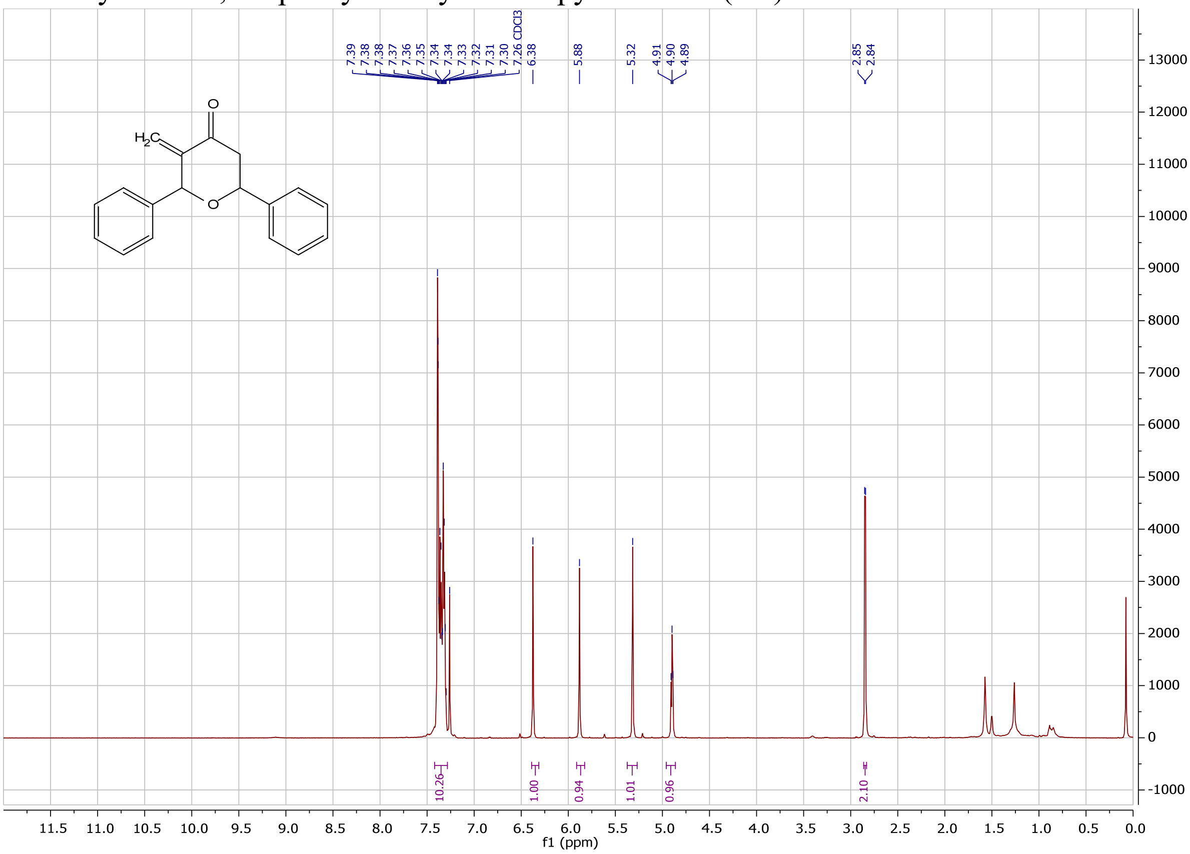

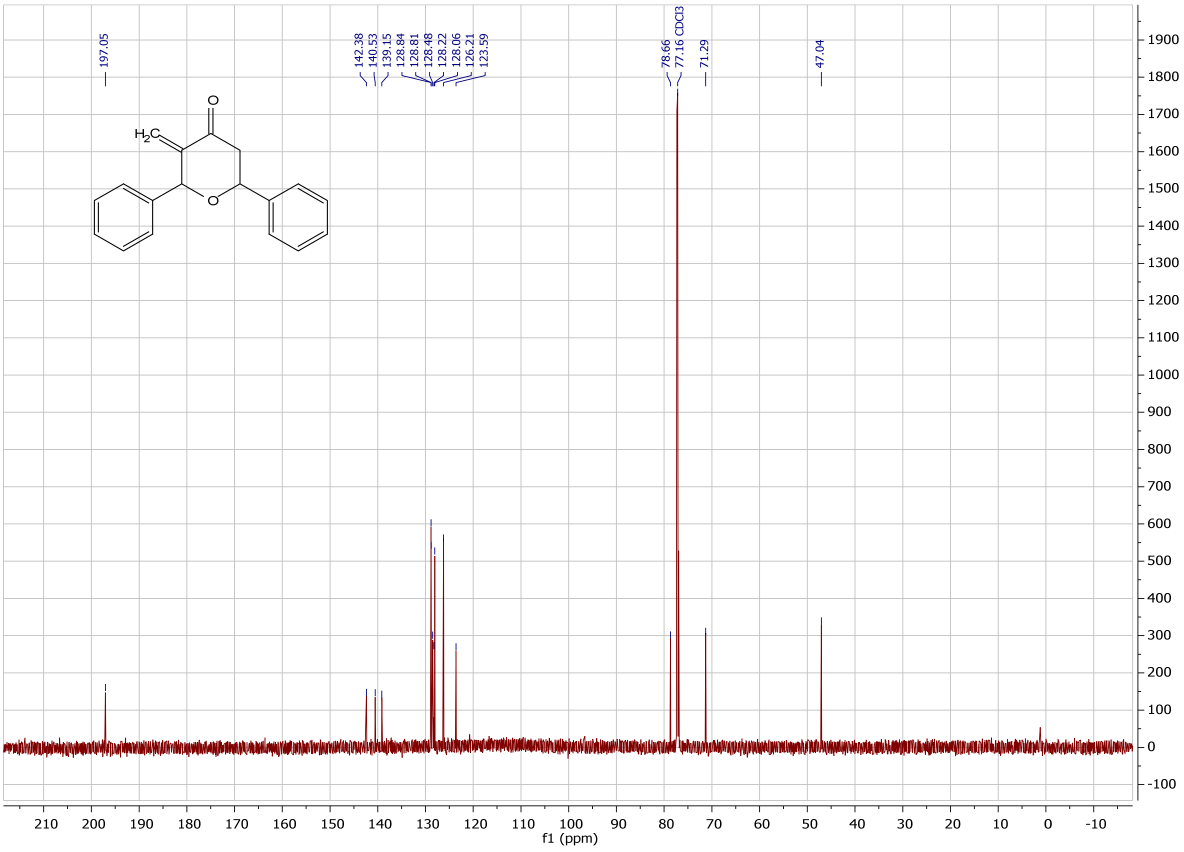

2-Isopropyl-3-methylidene-6-(4-methoxyphenyl)tetrahydro-4H-pyran-4-one (**13j**)

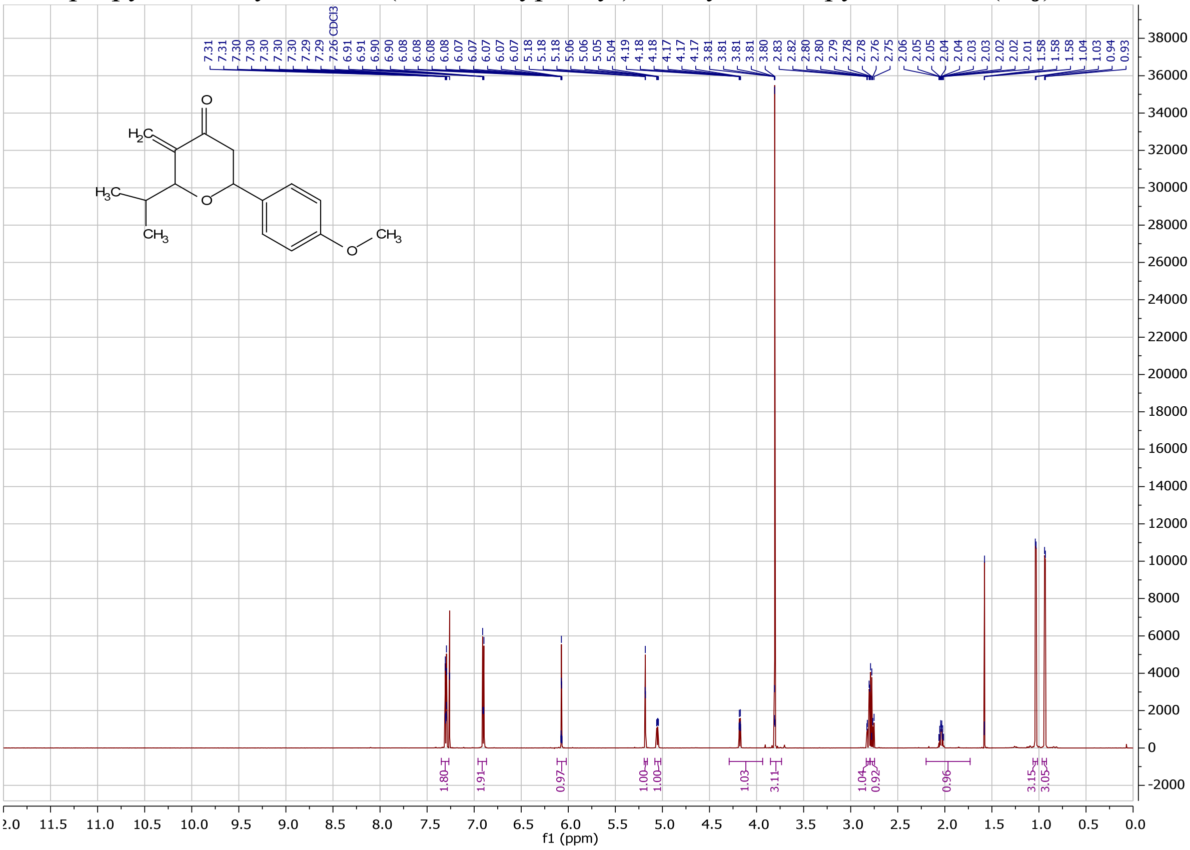

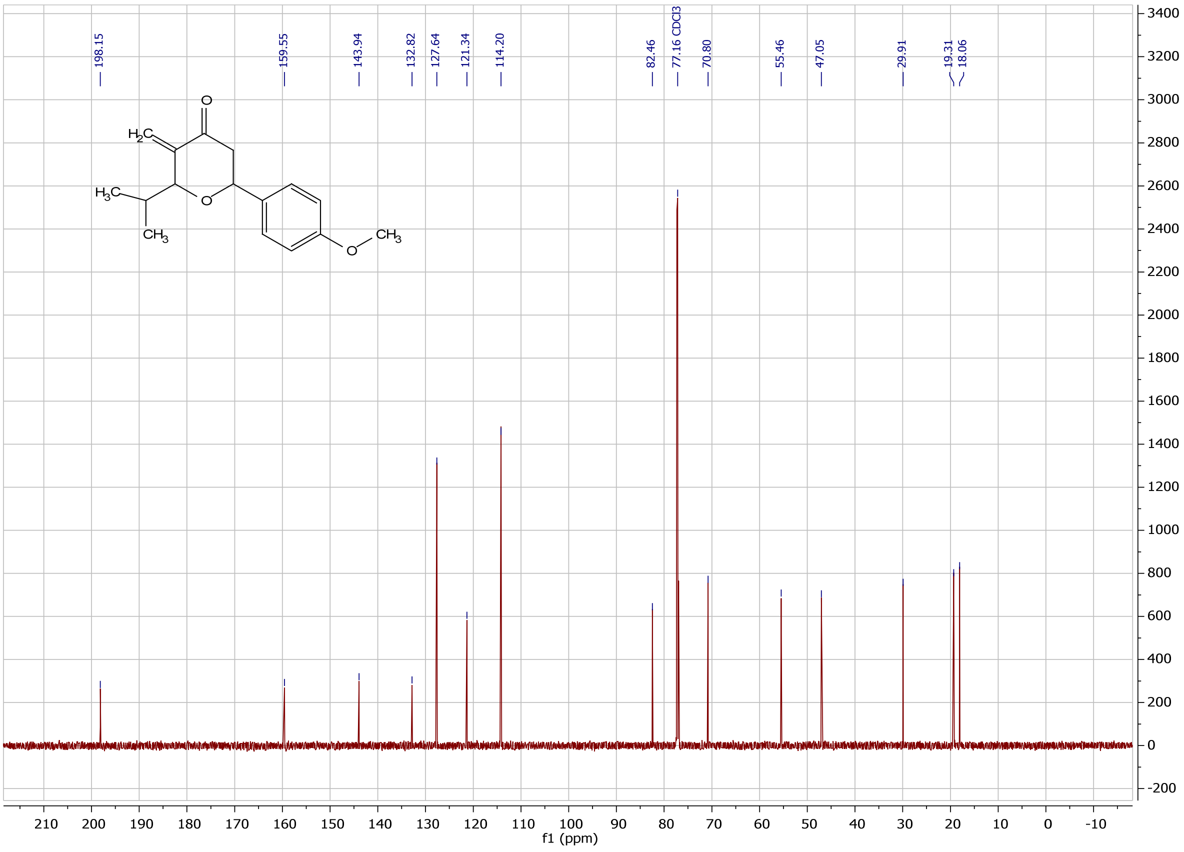

2-Buthyl-3-methylidene-6-(4-methoxyphenyl)tetrahydro-4H-pyran-4-one (**13k**)

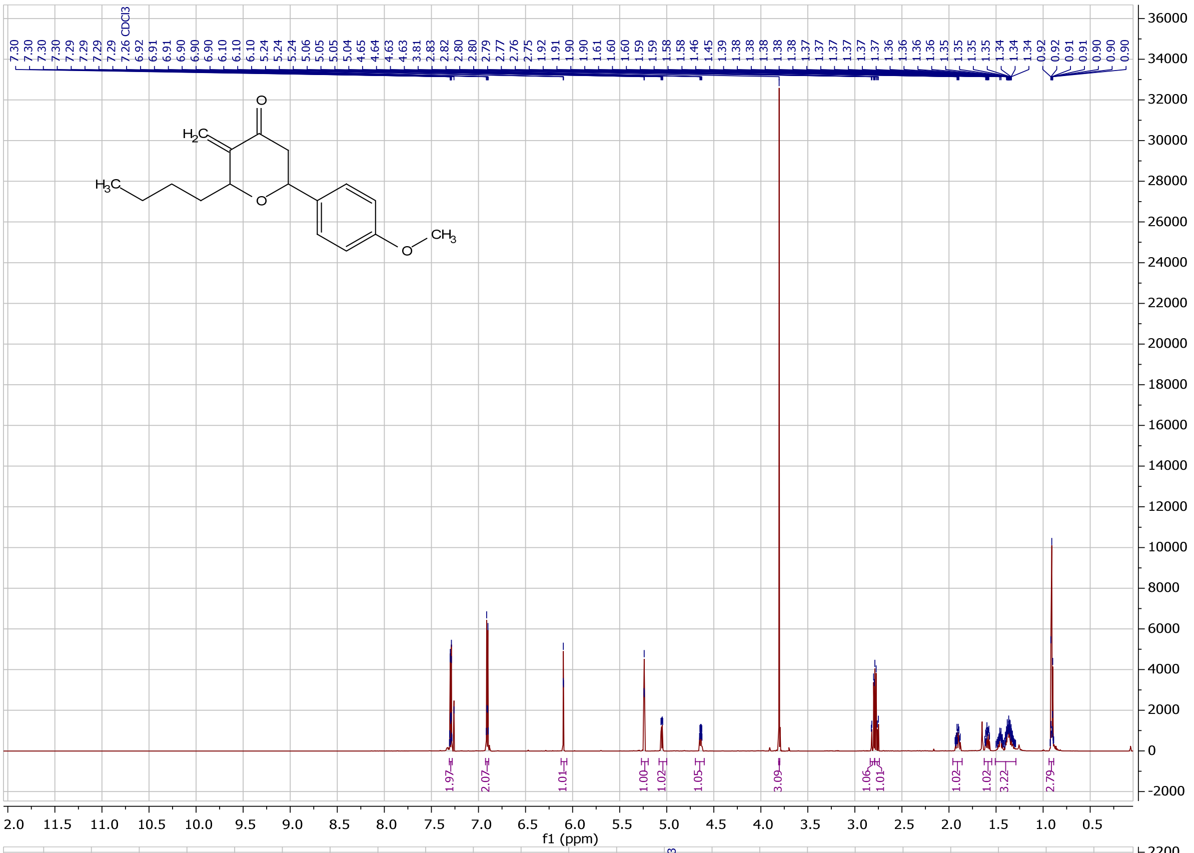
f1 (ppm)

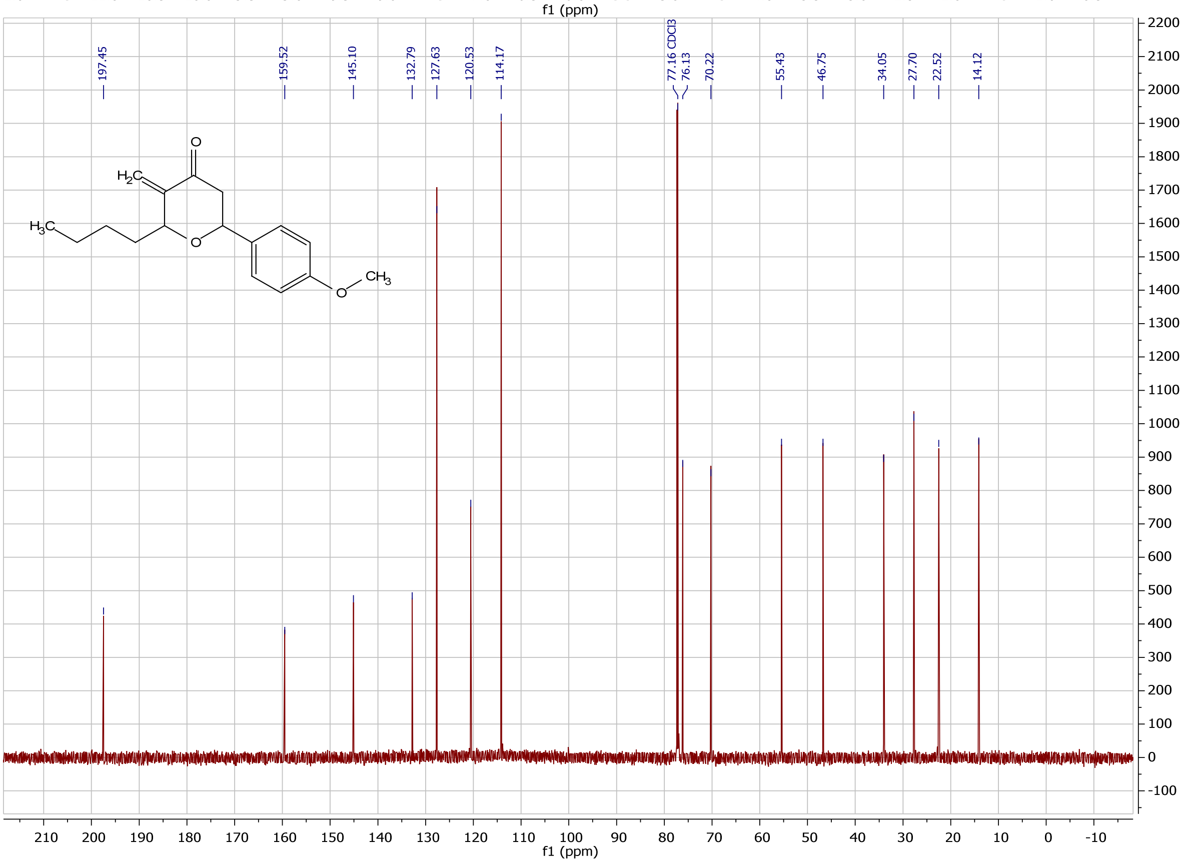
f1 (ppm)

3-methylidene-6-(4-methoxyphenyl)-2-phenyltetrahydro-4H-pyran-4-one (**13l**)

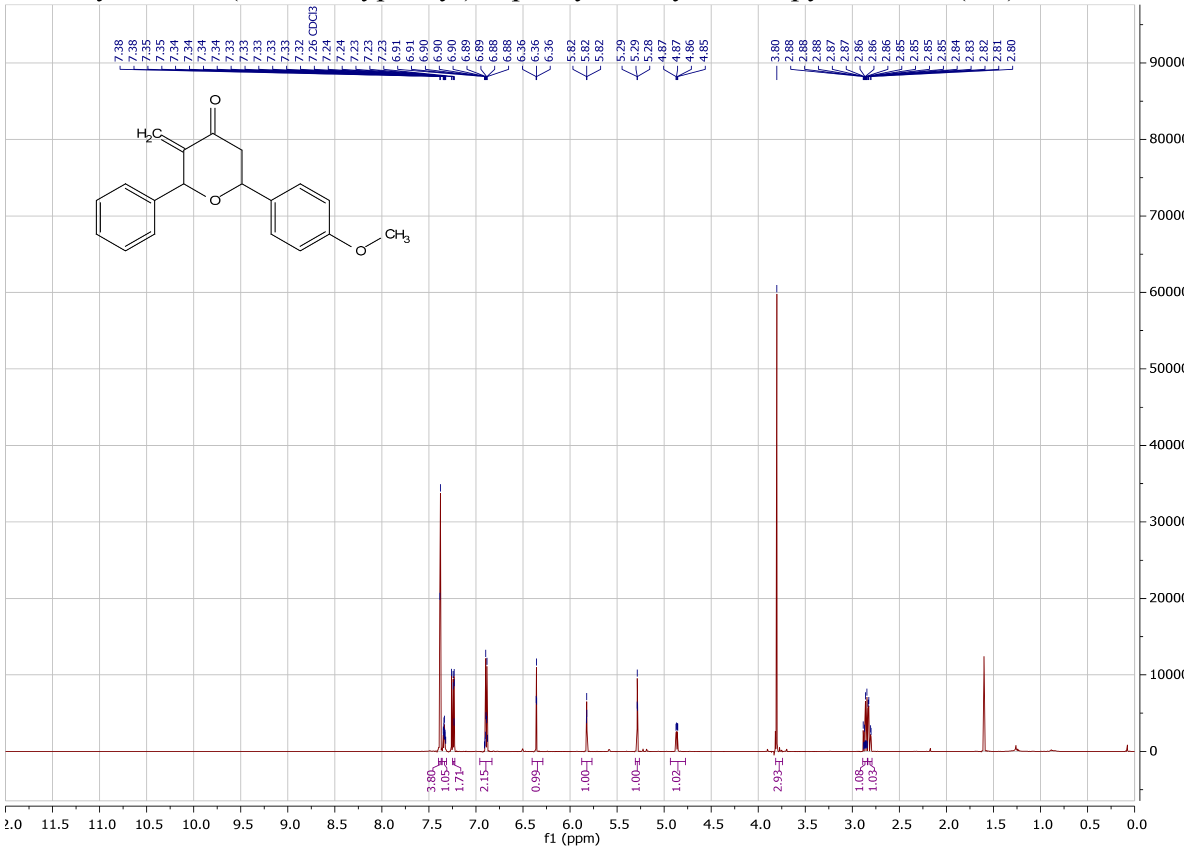

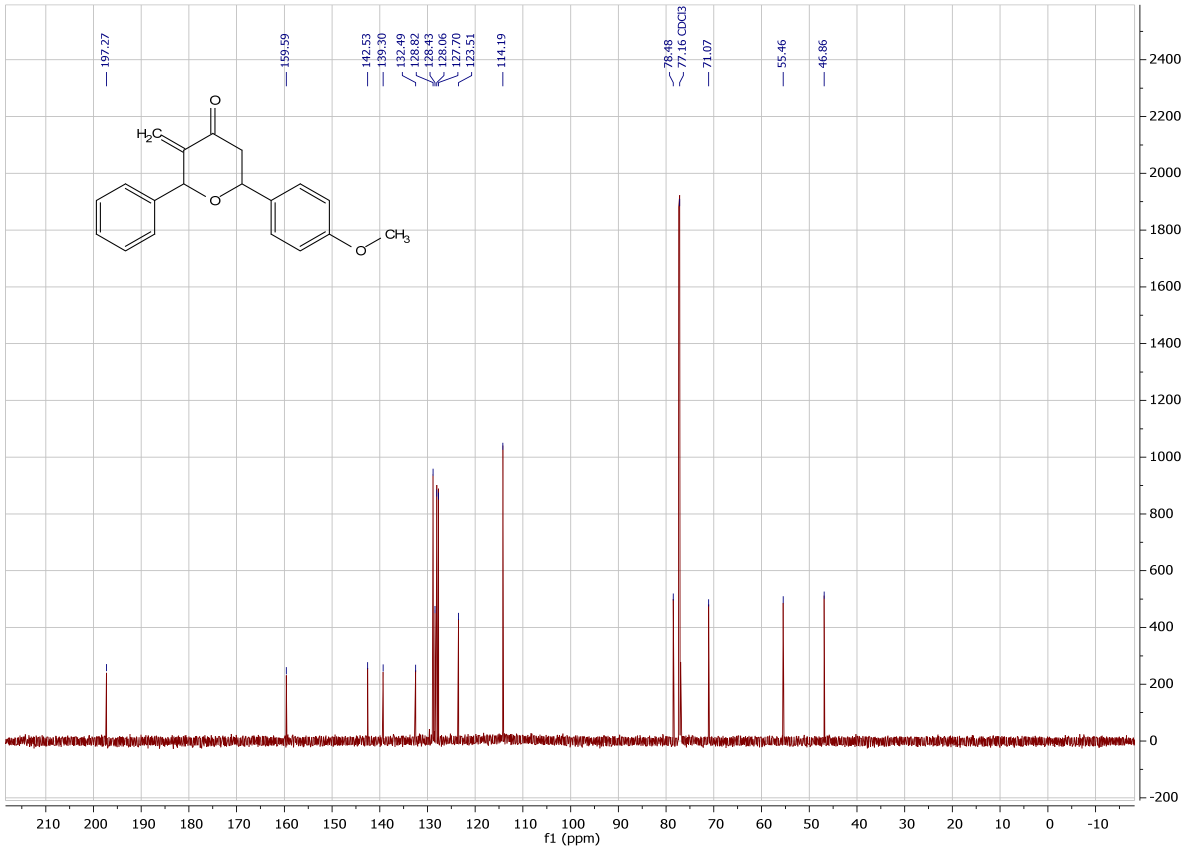

6-Ferrocenyl-2-isopropyl-3-methylidenetetrahydro-4H-pyran-4-one (**13m**)

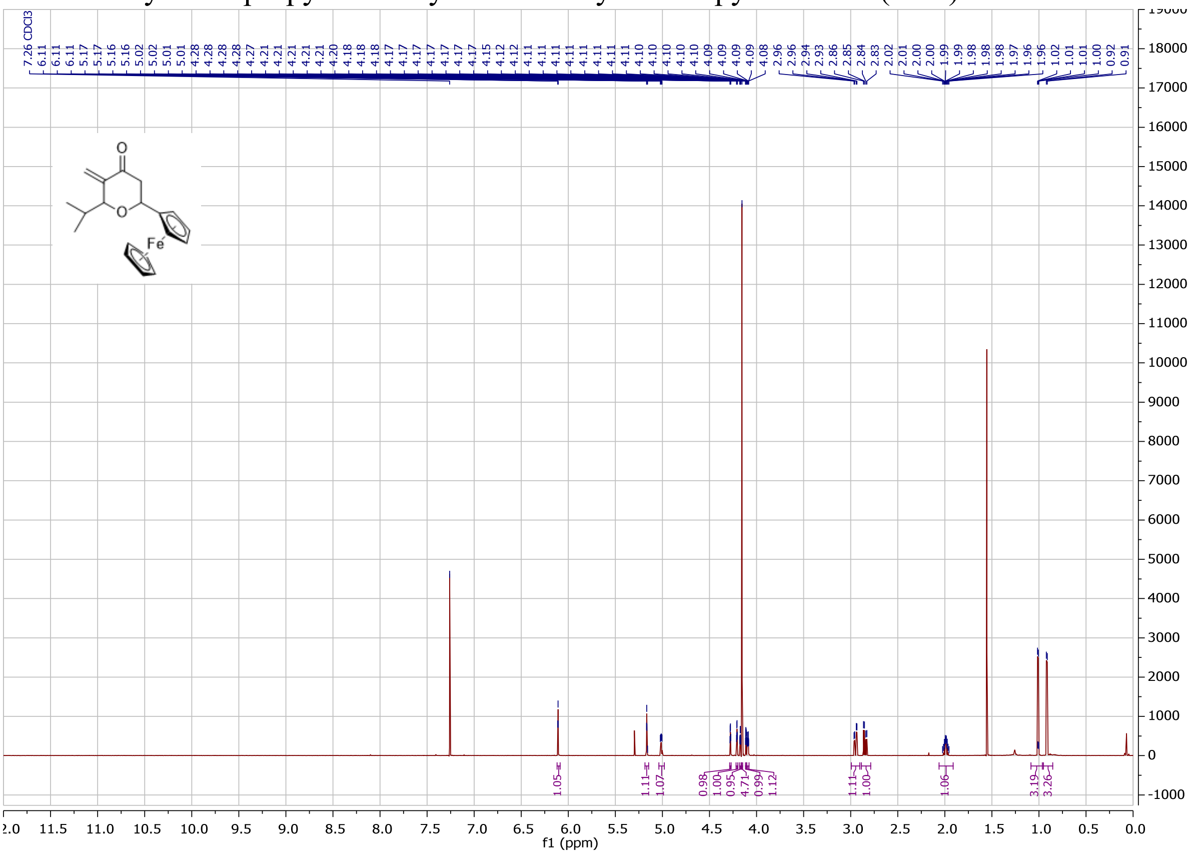

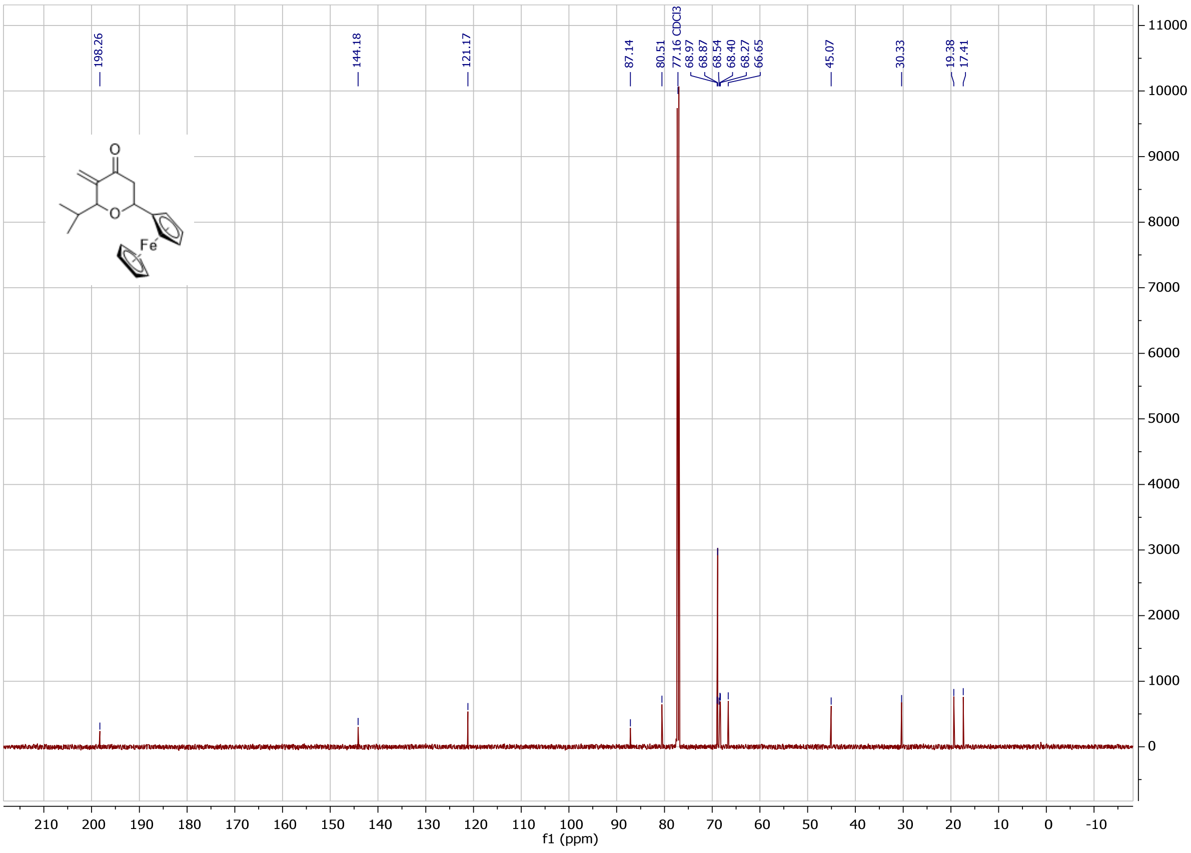

2-Buthyl-6-ferrocenyl-3-methylidenetetrahydro-4H-pyran-4-one (**13n**)

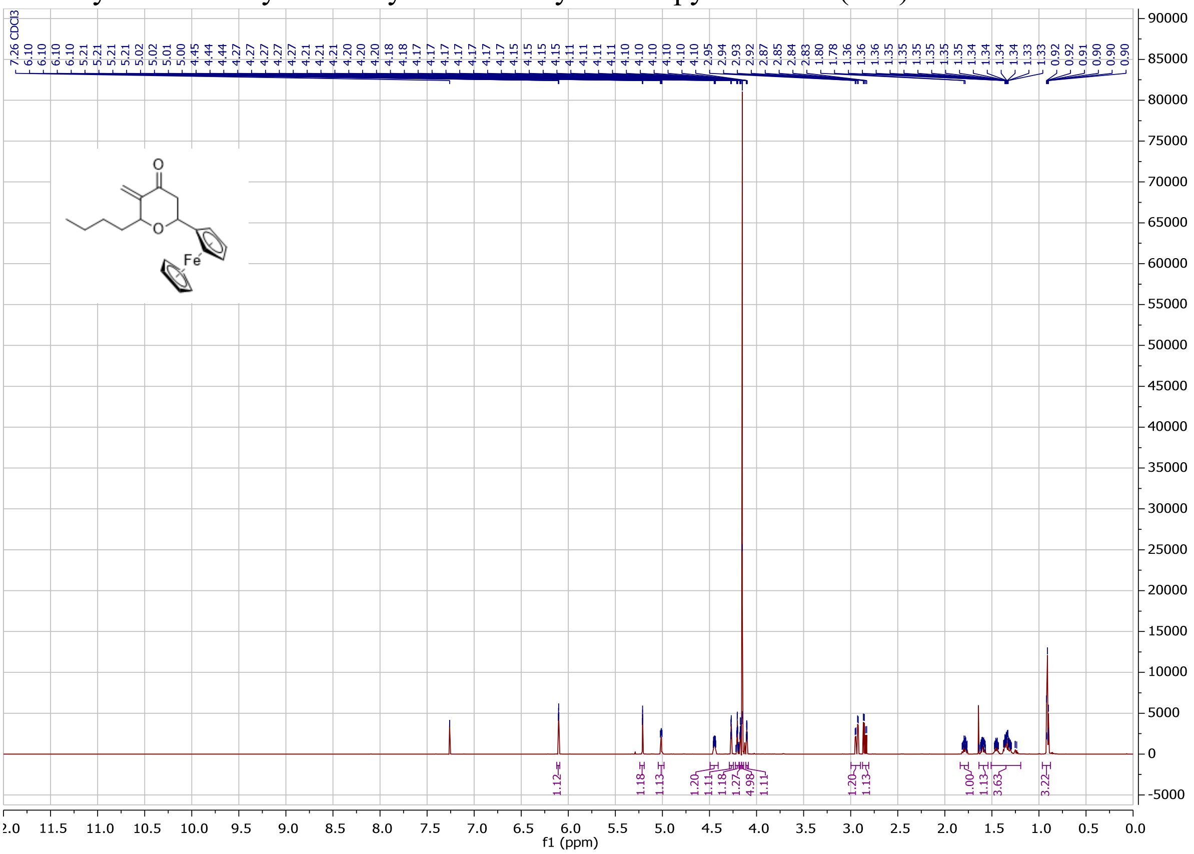

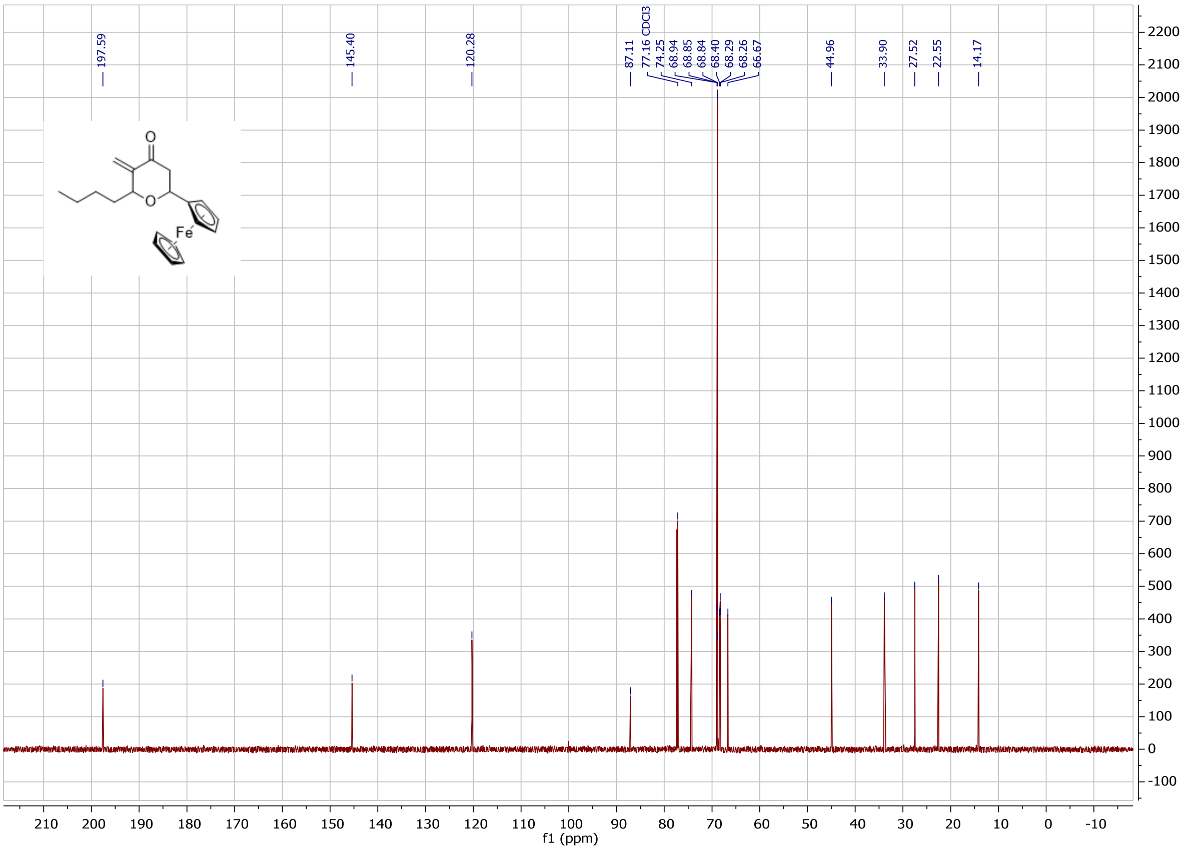

6-ferrocenyl-3-methylidene-2-phenyltetrahydro-4H-pyran-4-one (**13o**)

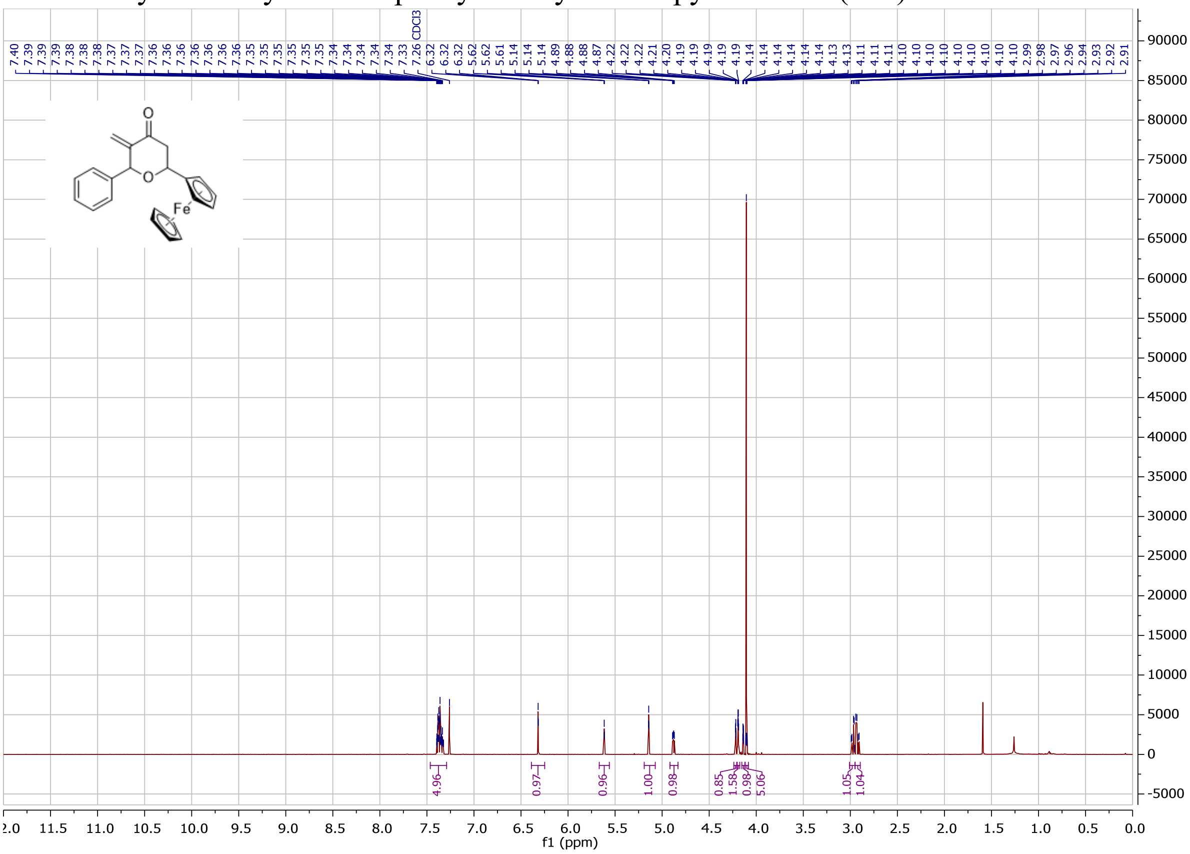

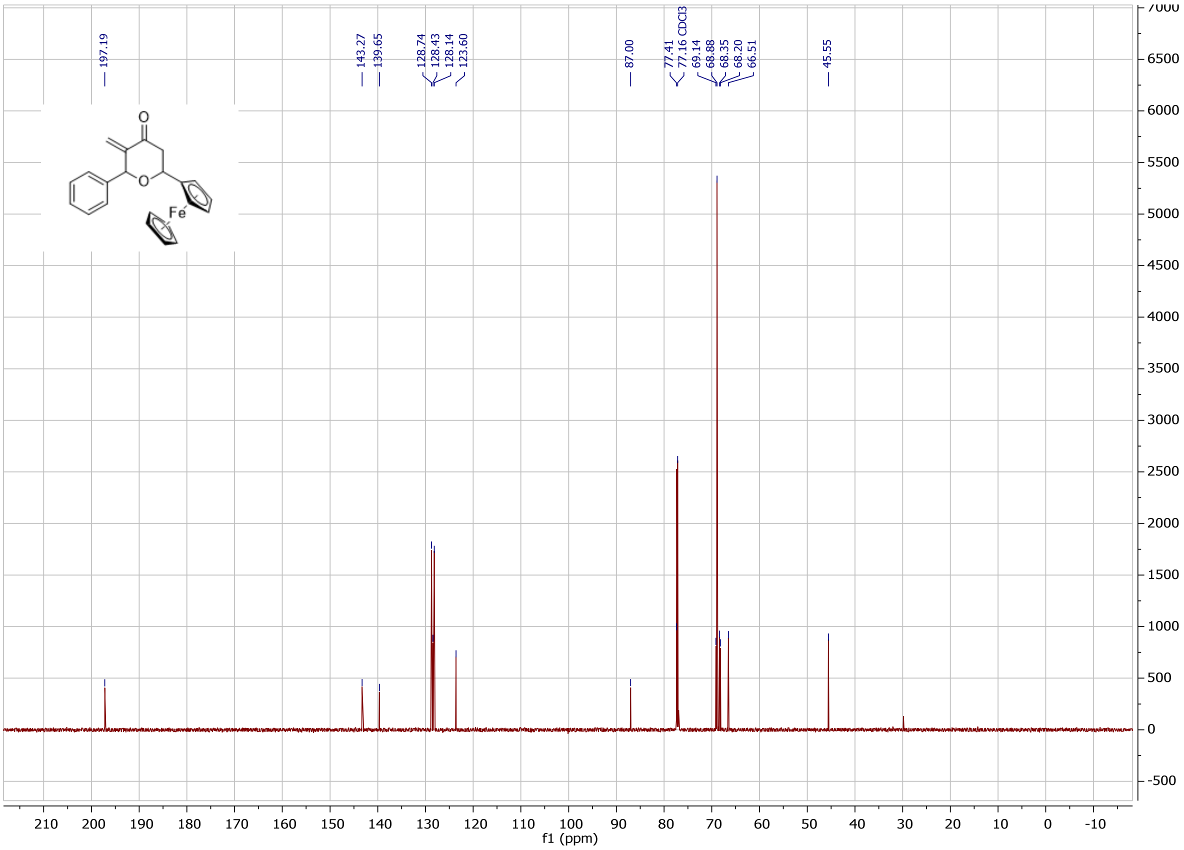

3-methylidene-6-(4-methoxyphenyl)tetrahydro-4H-pyran-4-one (**15d**)

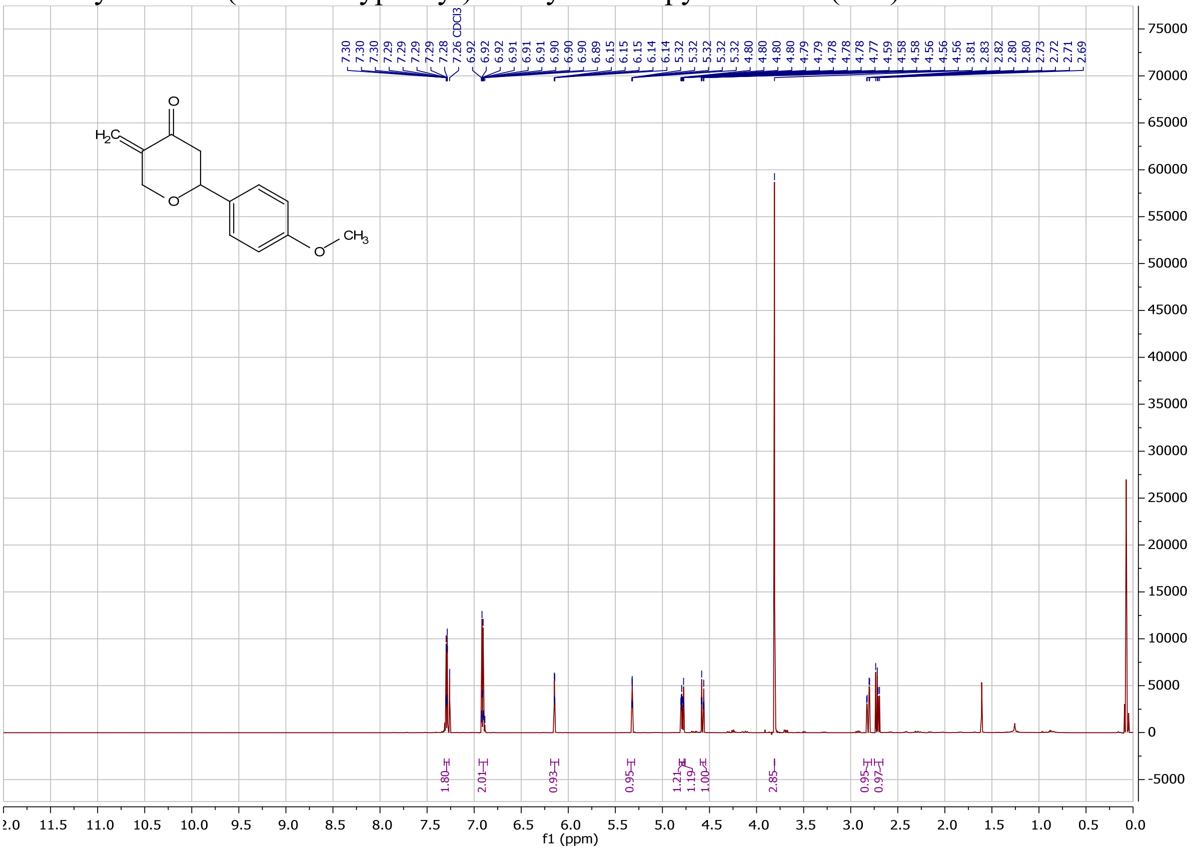

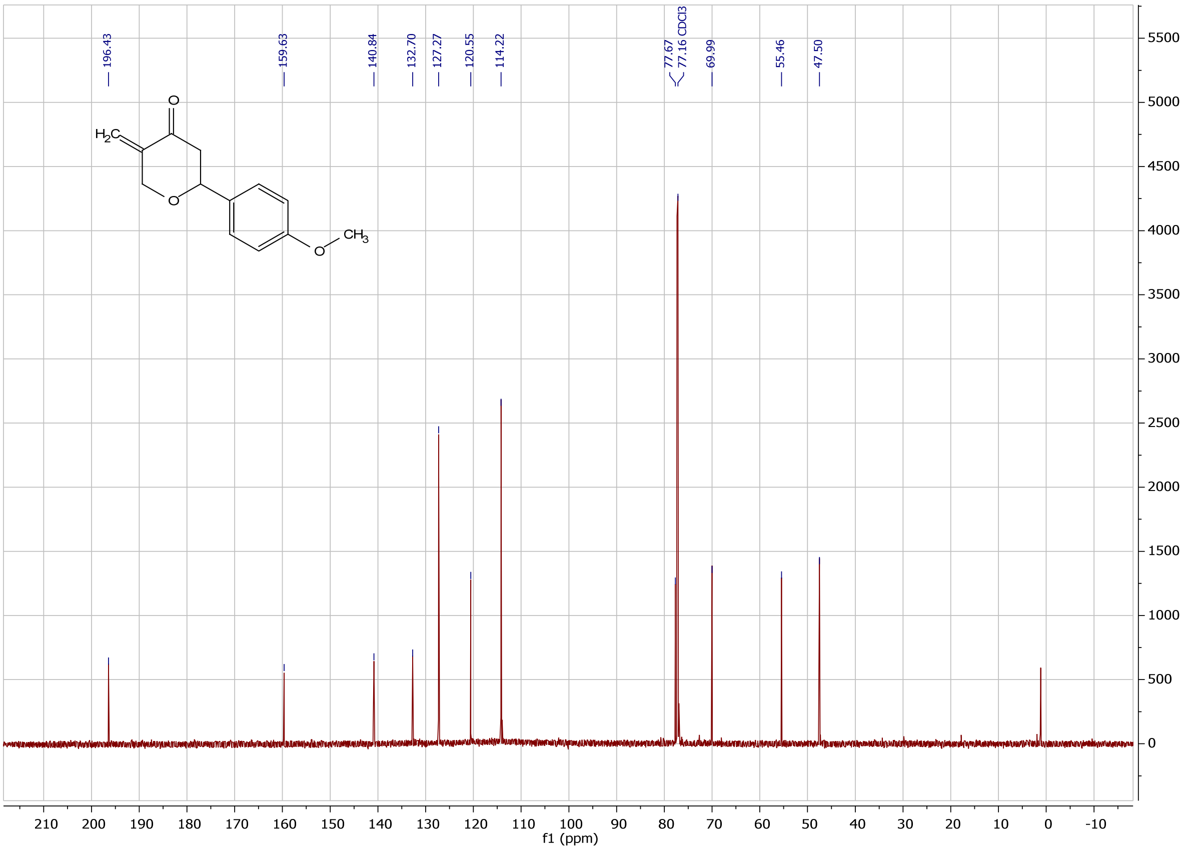

Supplement: Supplementary file 1 [file materials-15-03030-s001.zip › materials-1653665-supplementary.pdf]
